# Modulating Luminescence Thermometry of the Eu^III^/Tb^III^ Pair in Coordination Polymers through Ligand Structure and Lanthanide(III) Molar Ratio

**DOI:** 10.1021/acsomega.6c03259

**Published:** 2026-07-10

**Authors:** Gabriel J. S. Araujo, Talita C. Souza, Stefano A. de Andrade, Jéssica F. Rodrigues, Sergio F. N. Coelho, Pedro H. O. Santiago, Javier Ellena, Italo O. Mazali, Leonardo F. Saraiva, Airton G. Bispo-Jr, Fernando A. Sigoli

**Affiliations:** † Institute of Chemistry, State University of Campinas, Campinas, São Paulo 13083-970, Brazil; ‡ Institute of Physics, 28133 University of São Paulo, São Carlos, São Paulo 13566-590, Brazil; § Department of Chemistry and Biochemistry, 28108 São Paulo State University (UNESP), School of Science and Technology, São Paulo, São Paulo 19060-900, Brazil; ∥ Institute of Chemistry, University of São Paulo, São Paulo, São Paulo 05508-000, Brazil

## Abstract

Accurate temperature sensing at the nanoscale has become a powerful tool to monitor heat dissipation in miniaturized systems, driving the development of noncontact luminescent thermometers with high sensitivity. Eu^III^/Tb^III^-based coordination polymers are particularly attractive for thermometry due to their structural tunability, which allows the correlation of structural and electronic parameters with thermometric performance. Herein, we compare the luminescence thermometry of a series of one-dimensional coordination polymers, [Ln­(tfa)_3_(μ-dppeo)]_n_, [Ln­(tfa)_3_(μ-dppbo)]_n_, [Ln­(hfa)_3_(μ-dppeo)]_n_, and [Ln­(hfa)_3_(μ-dppbo)]_n_ (Ln = Eu^III^, Tb^III^; tfa^–^: trifluoroacetylacetonate; hfa^–^: hexafluoroacetylacetonate; L: [(diphenylphosphoryl)*R*]­(diphenyl)­phosphine oxide, *R* = ethyl - dppeo - or butyl - dppbo), focusing on the combined effects of bridge and terminal ligand scaffold as well as lanthanide (Ln^III^) molar ratio. For each system, the Tb^III^/Eu^III^ ratio was adjusted to {Tb_0.25_Eu_0.75_}_n_ or {Tb_0.75_Eu_0.25_}_n_, enabling systematic evaluation of ratiometric thermometry based on the Tb^III 5^D_4_ → ^7^F_5_ and Eu^III 5^D_0_ → ^7^F_2_ emissions. Structural variations induced by the terminal and bridge ligands modulate crystal packing, local coordination microsymmetry, and Ln^III^–Ln^III^ distances. Coordination polymers with higher Tb^III^ content exhibit enhanced thermometric performance, reaching a maximum relative thermal sensitivity of 5.20% K^–1^ at 305 K for [Tb_0.75_Eu_0.25_(hfa)_3_(μ-dppbo)]_n_. The increase of the Tb^III^ amount potentializes the Tb^III^-to-Eu^III^ energy transfer and enhances the activation energy of the luminescence thermal quenching. These results demonstrate that controlling the ligand scaffold and Ln^III^ molar ratio enables effective modulation of thermal sensitivity and operating range in Eu^III^/Tb^III^ coordination polymers.

## Graphical abstract

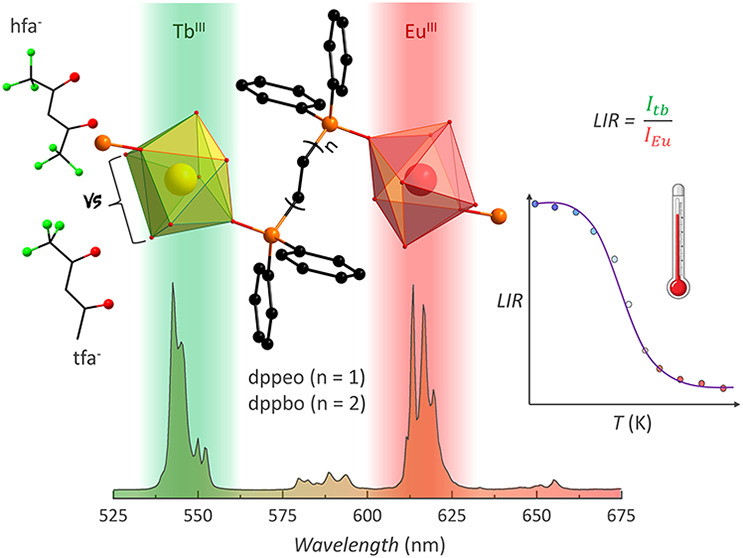

## Introduction

Accurate temperature measurement at the nanoscale is essential for understanding and controlling heat dissipation in miniaturized systems, where local thermal gradients can influence physical, chemical, and biological processes.^1–4^ For biomedical hyperthermia applications, for instance, high-resolution, real-time temperature monitoring could enable precise thermal control, thereby minimizing damage to healthy tissues during the thermal treatment of tumors.^5^ On the other hand, in microelectronics, thermal maps could be helpful for identifying operational hot spots in integrated circuits, guiding device design optimization.^6–8^ Lanthanide (Ln^III^)-based luminescence thermometry has emerged as a powerful approach for noncontact temperature sensing at the nanoscale due to the satisfactory thermal resolution (δ*T* < 0.1 K) and high (>1% K^–1^) relative thermal sensitivity (*S*_R_).^9,10^ These features arise from the bright luminescence offered by Ln^III^ compounds, long-lived excited levels (∼10^–3^ s), and temperature-dependent luminescence described by well-established models.^11–13^ Therefore, research in luminescent thermometry has increasingly focused on elucidating how structural and electronic parameters can be precisely tuned to control and enhance thermometric performance.^14^

Coordination polymers containing Eu^III^ and Tb^III^ constitute a versatile platform for lanthanide­(III) luminescence thermometry.^15^ These compounds have also been explored for a range of sensing applications, including the detection of benzaldehyde vapor,^16^ the recognition of arginine and lysine in living cells,^17^ and the identification of tryptophan and cancer biomarkers in biological environments.^18^ In these systems, the ligands absorb excitation energy and transfer it to both lanthanide ions, while Tb^III^ can also partially transfer energy to Eu^III^.^19^ This sensitization pathway, which is dependent on the temperature, enables ratiometric thermometry based on the intensity ratio of the emission bands associated with the Tb^III 5^D_4_→^7^F_5_ and Eu^III 5^D_0_→^7^F_2_ transitions.^20^ Intramolecular energy transfer (IET) between ligand and Ln^III^ excited levels, as well as between Ln^III^ centers, depends on the donor–acceptor distance, since the process occurs via nonradiative exchange or multipolar mechanisms.^21^ Exchange interactions require short donor–acceptor distances (up to ∼4 Å) due to orbital overlap, whereas multipolar interactions rely on resonant spectral overlap and can operate over much longer distances, with efficiencies that decrease with increasing separation.^22^ Considering these mechanisms, coordination polymers constitute well-suited platforms for investigating luminescent thermometric behavior, as their crystalline arrangement and Ln^III^ – Ln^III^ distances can be tuned through the choice of terminal and bridge ligands.^23–27^ For example, Hatanaka used the [Tb_0.99_Eu_0.01_(hfa)_3_(linker)]_n_ coordination polymer (linker is dpb: 1,4-bis­(diphenylphosphoryl)­benzene, dppcz: 3,6-bis­(diphenylphosphoryl)-9-phenylcarbazole, or dpbt: 4,40 -bis­(diphenylphosphoryl)­bithiophene; hfa^–^: hexafluoroacetylacetonate) to demonstrate that Tb^III^-to-Eu^III^ energy transfer (ET) occurs up to distances as high as 13.6 Å, directly impacting the luminescence thermometry.^28^

Phosphine oxides are widely employed as bridge ligands, while β-diketonates are commonly explored as terminal ligands, acting as efficient luminescent antennas to sensitize the Ln^III^ luminescence.^29^ Among these compositions, numerous studies have focused on elucidating how Tb^III^-to-Eu^III^ ET and crystal structure affect the thermometric performance. For example, Miyata and co-workers reported a luminescent thermometer based on [Ln­(hfa)_3_(dpbp)]_n_ (dpbp: 4,4′-bis­(diphenylphosphoryl)­biphenyl; Ln: Eu^III^ and Tb^III^) coordination polymer by taking advantage of the temperature dependence of the Tb^III^-to-Eu^III^ ET on the Eu^III^ and Tb^III^ emission intensities.^30^ Apart from the ratiometric thermometry, Kitagawa and co-workers employed the Eu^III 5^D_0_ lifetime as a thermometric parameter in [Eu_m_Gd_1–m_(hfa)_3_(bdpc)]_n_ (bdpc = 6,12-bis­(diphenylphosphoryl)­chrysene), which presents a zigzag-oriented single polymeric chain, offering maximum relative thermal sensitivity of 3.73% K^–1^ at 450 K.^31^

We recently reported two 1D-coordination polymers based on [Eu_0.5_Tb_0.5_(tfa)_3_(μ-L)]_n_ (tfa^–^: trifluoroacetylacetonate and L: [(diphenylphosphoryl)*R*]­(diphenyl)­phosphine oxide, *R* = ethyl - dppeo - or butyl - dppbo) exhibiting luminescence thermometry.^32^ In these systems, dppeo leads to an almost linear 1D chain while dppbo induces a zigzag structure, imparting shorter Ln^III^–Ln^III^ distances and boosting the maximum relative thermal sensitivity from 2.6% K^–1^ (dppeo as bridge) to 3.8% K^–1^ (dppbo as bridge).^32^ Calculations of the luminescence dynamics provide evidence that reducing the Eu^III 7^F_0_–^7^F_1_ energy splitting, which can be tuned by the Eu^III^ coordination sphere, enhances the thermal dependence of both ligand-to-Ln^III^ sensitization and Tb^III^-to-Eu^III^ ET.^33^ The backbone architectures of these coordination polymers were also employed to achieve quantum cutting emission in the [Ln­(tfa)_3_(μ-L)]_n_ (Ln: Tb^III^, Yb^III^) compositions.^34^ The same phosphine oxide ligands were combined with hfa^–^ in the terminal positions to synthesize the compositions [Ln­(hfa)_3_(μ-L)]_n_ (Ln: Er^III^, Yb^III^), presenting molecular upconversion emission.^35^ In this case, both coordination polymers are based on 1D chains with an almost linear conformation, although the Ln^III^–Ln^III^ distances vary systematically.^35^ In these coordination polymers, the Ln^III^ concentration is expected to strongly influence luminescent thermometry, as it governs the Tb^III^–Eu^III^ separation and, consequently, the ET dynamics.^27,34^ Nevertheless, the impact of Ln^III^ amount on the thermometric behavior of such coordination polymers remains relatively underexplored.

Considering these aspects, coordination polymers incorporating dppeo or dppbo as bridge ligands and hfa^–^ or tfa^–^ as terminal ligands provide a versatile platform to investigate the tunability of Eu^III^/Tb^III^ ratiometric luminescence thermometry. Built on this, herein, we compare the luminescence thermometry performance of the coordination polymers [Ln­(tfa)_3_(μ-dppeo)]_n_, [Ln­(tfa)_3_(μ-dppbo)]_n_, [Ln­(hfa)_3_(μ-dppeo)]_n_, and [Ln­(hfa)_3_(μ-dppbo)]_n_ (Ln: Eu^III^, Tb^III^), focusing on the effect of molar Ln^III^ ratios—{Tb_0.75_Eu_0.25_}_n_ or {Tb_0.25_Eu_0.75_}_n_—and bridge and terminal ligand nature. Both the ligand scaffold and the Ln^III^ molar ratio govern the maximum relative thermal sensitivity and the operational temperature range by modulating the crystal structure and Ln^III^–Ln^III^ distances, which, in turn, affect the dynamics of luminescence thermal quenching.

## Results and Discussion

### Structural Characterization

The coordination polymers were synthesized by mixing the [Ln­(L^1^)_3_(H_2_O)_2_] (L^1^ = tfa^–^ or hfa^–^, Ln = Tb^III^, Eu^III^) precursor with the bridge ligand (dppeo or dppbo) in ethanol at 300 K, following a procedure previously reported by us.^32,35^ Needle-like crystals were obtained after 3 days, which were filtered and used in all the analyses. For each combination of bridge and terminal ligands—[Ln­(tfa)_3_(μ-dppeo)]_n_, [Ln­(hfa)_3_(μ-dppeo)]_n_, [Ln­(tfa)_3_(μ-dppbo)]_n_, and [Ln­(hfa)_3_(μ-dppbo)]_n_—the Tb^III^-to-Eu^III^ molar ratio was adjusted to either {Tb_0.25_Eu_0.75_}_n_ or {Tb_0.75_Eu_0.25_}_n_, resulting in a total of eight samples (Table S1). Further details regarding the synthesis and characterization apparatus are described in Supporting Information note S1.

The crystalline structures of [Ln­(tfa)_3_(μ-dppeo)]_n_, [Ln­(hfa)_3_(μ-dppeo)]_n_, [Ln­(tfa)_3_(μ-dppbo)]_n_, and [Ln­(hfa)_3_(μ-dppbo)]_n_ (Ln = Er_0.5_Yb_0.5_) coordination polymers were previously reported and discussed in detail by us.^35^ The four systems crystallize in the *P*1̅ space group and form 1D-coordination polymers. Except for [Ln­(tfa)_3_(μ-dppbo)]_n_, which presents a zigzag topology, the others organize into almost linear 1D chains, Figure 1A–D. The coordination number of the Ln^III^ sites is eight owing to three bidentate β-diketonate ligands and two bidentate nonchelating phosphine oxide coordinated by oxygen atoms, which bridge two Ln^III^ centers. The first coordination environment is described by a distorted D_4d_ point group, except for [Ln­(tfa)_3_(μ-dppbo)]_n_, whose coordination sphere is described by a D_2d_ pseudo symmetry.

**Figure 1:**
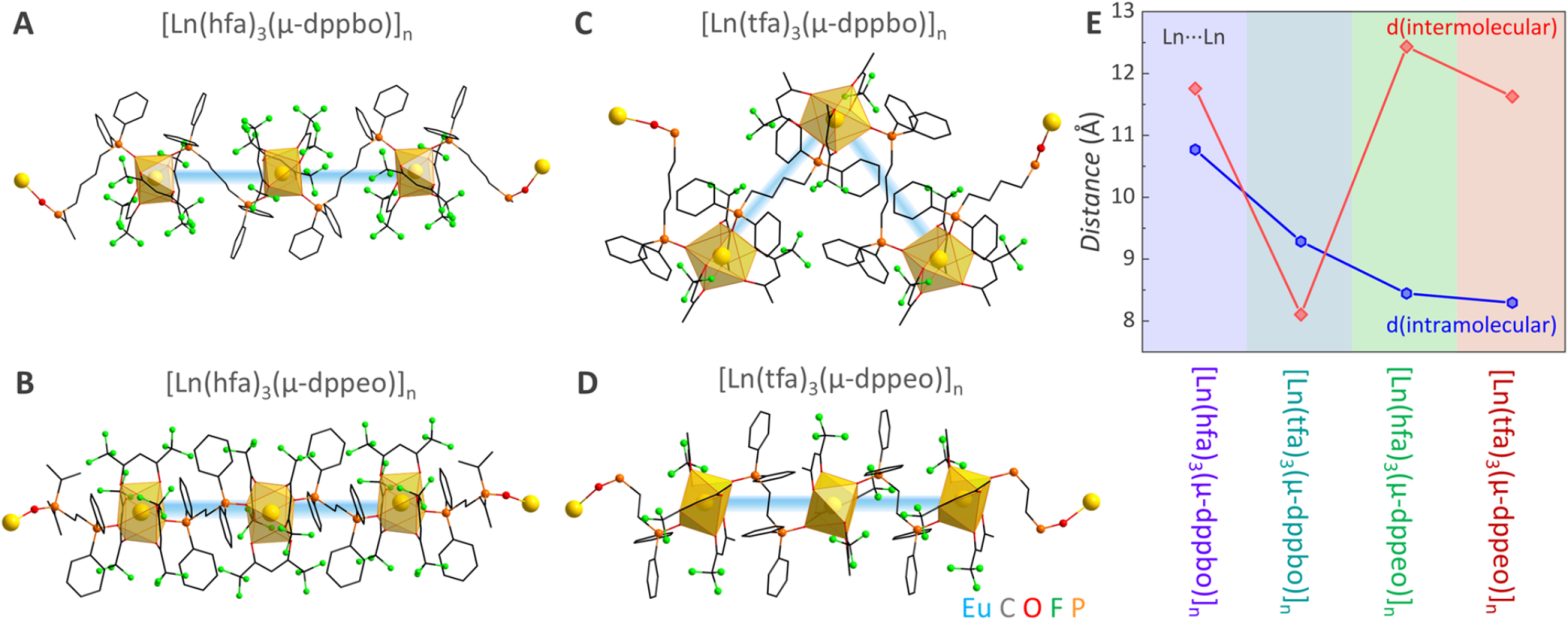
Crystal structures of (A) [Ln­(hfa)_3_(μ-dppbo)]_n_, (B) [Ln­(hfa)_3_(μ-dppeo)]_n_, (C) [Ln­(tfa)_3_(μ-dppbo)]_n_, and (D) [Ln­(tfa)_3_(μ-dppeo)]_n_ (Ln = Eu^III^, Tb^III^). H atoms were removed for the sake of clarity. The crystal structures of [Ln­(tfa)_3_(μ-dppeo)]_n_ and [Ln­(hfa)_3_(μ-dppeo)]_n_ were first reported by us elsewhere (CCDC code 2381906 and 2381911).^32,35^ (E) Ln^III^···Ln^III^ inter/intramolecular distances in the coordination polymers.

The nature of the terminal and bridge ligands induces variations in the inter- and intramolecular Ln^III^···Ln^III^ separations within the crystal structure; in particular, the [Ln­(tfa)_3_(μ-dppbo)]_n_ framework exhibits shorter intermolecular Ln^III^···Ln^III^ distances (Figure 1E and Table S2) because of its zigzag arrangement. Such structural variations are expected to affect the Tb^III^→Eu^III^ ET process, which has an important role in the thermometric performance of the coordination polymers.^33^

To confirm the successful synthesis of the coordination polymers, single-crystal X-ray diffraction (SC-XRD) was collected for the [Tb_0.75_Eu_0.25_(tfa)_3_(μ-dppbo)]_n_ and [Tb_0.75_Eu_0.25_(hfa)_3_(μ-dppeo)]_n_ compositions (Figure 1B,C and Table S3). The collected SC-XRD data match those already reported by us for [Er_0.5_Yb_0.5_(tfa)_3_(μ-dppbo)]_n_ and [Er_0.5_Yb_0.5_(hfa)_3_(μ-dppeo)]_n_,^35^ confirming that the samples are isostructural and isomorphic. Moreover, the experimental powder X-ray diffraction (PXRD) patterns of all samples (Figure S1) are consistent with those simulated from the SC-XRD data measured for [Tb_0.75_Eu_0.25_(tfa)_3_(μ-dppbo)]_n_ and [Tb_0.75_Eu_0.25_(hfa)_3_(μ-dppeo)]_n_ and those previously reported by us (CCDC 2381906, 2381909, 2381910, 2381911) for the {Er_0.5_Yb_0.5_}_n_ analogous.^35^ It should be noted that, for the [Ln­(hfa)_3_(μ-dppbo)]_n_ compositions (Figure S1A), two peaks near 9.6 and 10.4° are absent in the simulated PXRD patterns generated from the SC-XRD data, which is likely due to the presence of unreacted bridge ligand. Moreover, FTIR data agree with those expected for the coordination polymers (Figure S2). These results confirm the successful synthesis of the intended structures.^32,35^

### Steady-State and Time-Resolved Photoluminescence Spectroscopy

To understand the photophysical dynamics of the coordination polymers, the photoluminescence features were first investigated by excitation spectroscopy, monitoring the Eu^III^ emission. The excitation spectra at 300 K (Figure 2A,B) are dominated by two broad excitation bands peaking at 275 and 340 nm, which are assigned to ligand-centered π→π* (S_0_→S_n_) transitions. Besides the ligand-centered excitation, Eu^III^ *f–f* electronic transitions are also observed, yet the weaker intensity of these bands highlights the effective antenna effect played by the ligands to sensitize the Eu^III^ luminescence. By monitoring the Tb^III^ emission (Figure S3), only the ligand-centered excitation bands are detected, also confirming that the ligands sensitize the Tb^III^ luminescence in all of the coordination compounds.

**Figure 2:**
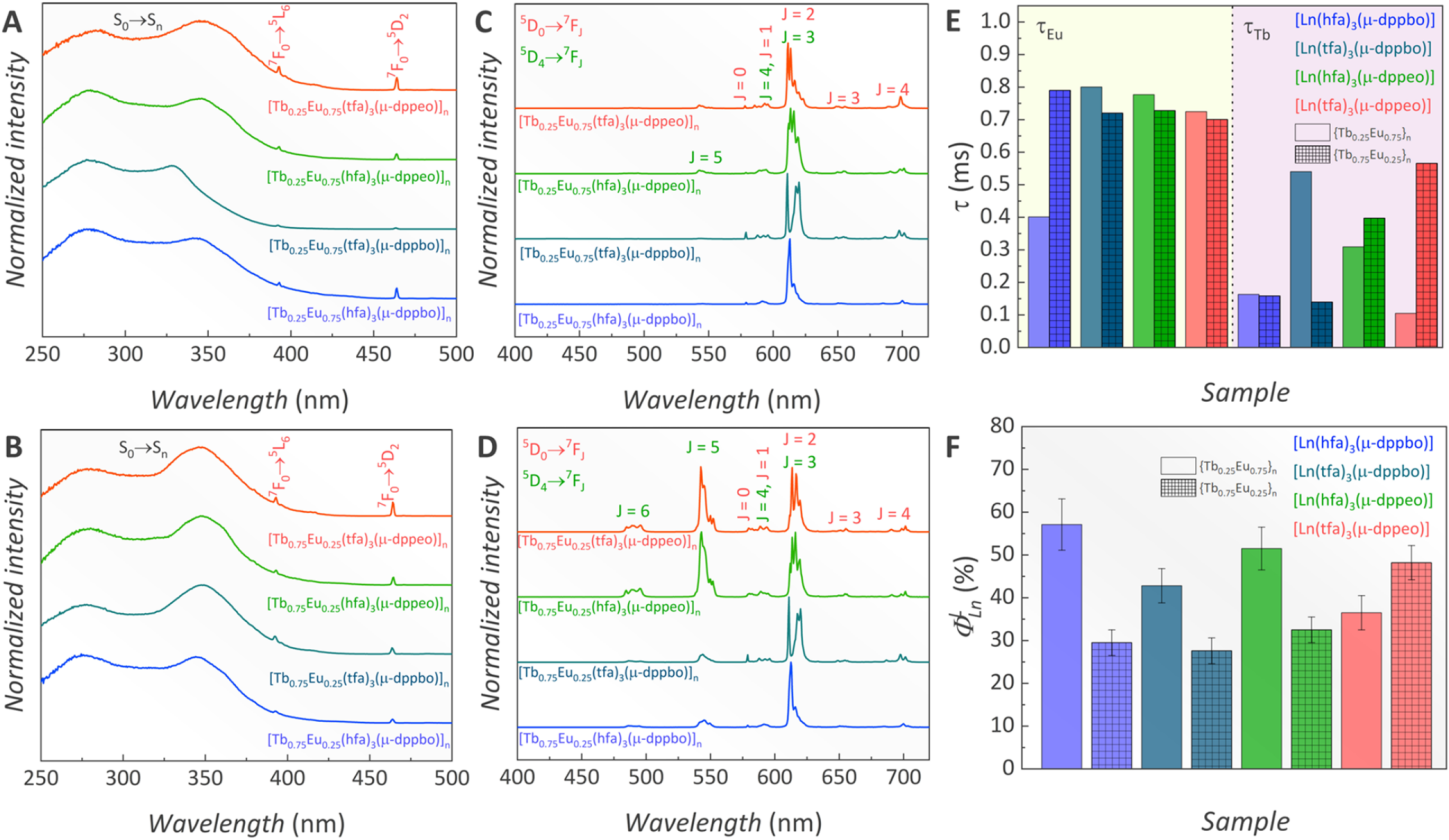
Excitation spectra (300 K) monitoring the Eu^III^ emission at 612 nm for the coordination compounds with composition (A) {Tb_0.25_Eu_0.75_}_n_ and (B) {Tb_0.75_Eu_0.25_}_n_. Emission spectra (300 K) upon 340 nm for the coordination compounds with composition (C) {Tb_0.25_Eu_0.75_}_n_ and (D) {Tb_0.75_Eu_0.25_}_n_; Eu^III^ and Tb^III^ transitions are represented in red and green, respectively. (E) Eu^III 5^D_0_ (τ_Eu_) and Tb^III 5^D_4_ (τ_Tb_) level lifetimes measured at 300 K. (F) Emission quantum yields (Φ_Ln_^L^) measured at 300 K.

Upon 340 nm excitation, both Eu^III^ and Tb^III^ emission bands are detected within the 450–720 nm spectral range (Figure 2C,D). The Eu^III^ emission bands are assigned to ^5^D_0_→^7^F_0–4_ transitions, with the hypersensitive ^5^D_0_→^7^F_2_ transition dominating the spectrum. This result indicates that Eu^III^ is inserted in low symmetry coordination environments lacking an inversion center, in accordance with SC-XRD data. The Tb^III^ emission bands, in turn, are assigned to ^5^D_4_→^7^F_6–0_ transitions, with the dominant band at 542 nm attributed to the ^5^D_4_→^7^F_5_ transition. As expected, increasing the Tb^III^ molar content leads to a higher relative intensity of the Tb^III^ emission bands (Figure 2C,D). As a consequence, the emission color shifts from red at lower Tb^III^ contents to yellow as the Tb^III^ fraction increases in most of the coordination polymers, as evidenced by the CIE 1931 chromaticity diagrams (Figure S4 and Table S4).

Our previous computational and experimental studies indicate that the luminescence dynamics of these coordination polymers is based on the contribution of an IET from the β-diketonate ligands to the Ln^III^ centers as well as Tb^III^-to-Eu^III^ ET, leading to Ln^III^ emission.^32,33^ To confirm that Tb^III^-to-Eu^III^ ET is indeed contributing to the process, the excitation spectra were undertaken by monitoring the Eu^III^ emission at 700 nm (Figure S5), where the Eu^III 5^D_0_→^7^F_4_ transition occurs with no overlap of any Tb^III^ process. Upon this condition, a weak band at 484 nm is detected and assigned to the Tb^III 7^F_6_→^5^D_4_ transition, indicating that Tb^III^-to-Eu^III^ ET is indeed occurring at 300 K.

Further details regarding the luminescence dynamics at 300 K were provided by time-resolved spectroscopy monitoring the ligand excitation (340 nm) and Tb^III^ (542 nm) or Eu^III^ (612 nm) emissions. The emission decay curves were fitted by a monoexponential function (Figures S6 and S7) to determine the Eu^III 5^D_0_ (τ_Eu_) and Tb^III 5^D_4_ (τ_Tb_) lifetimes (Table S5 and Figure 2E). First, we investigated the influence of the Ln^III^ content on the emitting-level lifetimes. When tfa^–^ is used as a terminal ligand, as the Tb^III^ content increases, the τ_Eu_ gets shorter while τ_Tb_ gets longer when dppeo is the bridge, but decreases when dppbo is employed. The shortening of τ_Tb_ suggests a prominent Tb^III^-to-Eu^III^ ET as the Tb^III^ loading increases, which is expected considering that [Ln­(tfa)_3_(μ-dppbo)]_n_ presents a zigzag structure with shorter Tb^III^–Eu^III^ intramolecular distances (Table S2 and Figure 1E), favoring the ET process. For the [Ln­(hfa)_3_(μ-dppeo)]_n_ series, as the Tb^III^ content increases, τ_Eu_ becomes slightly shorter while τ_Tb_ gets longer. On the other hand, in the [Ln­(hfa)_3_(μ-dppbo)]_n_ compositions, the τ_Tb_ barely changes, and τ_Eu_ becomes longer as the Tb^III^ content increases (Table S5 and Figure 2E). The enhancement of τ_Eu_ even though the Eu^III^ amount decreases is explained by an additional pumping to the emitting level stemming from Tb^III^ ET, implying a more prominent Tb^III^–Eu^III^ ET. Therefore, analyses of the emitting-level lifetime suggest that Tb^III^→Eu^III^ ET tends to become more prominent as the Tb^III^ content increases in the [Ln­(tfa)_3_(μ-dppbo)]_n_ and [Ln­(hfa)_3_(μ-dppbo)]_n_ systems, which is in accordance with other studies discussing the dynamics of the Eu^III^/Tb^III^ pair in similar systems.^26,36^

For dppeo-based systems, the dependence of Ln^III^ lifetime on the Ln^III^ content is more intricate, making it more challenging to draw definitive conclusions regarding the contribution of the Tb^III^→Eu^III^ ET process. In general, as the Ln^III^ concentration increases, the emitting-level lifetime tends to increase (up to a concentration quenching); however, if the ion acts as an energy donor to another center, a shortening of its lifetime is expected. Moreover, the excitation spectra (Figures 2A,B and S5) indicate that the ligand-to-Ln^III^ IET is expected to be more efficient than the Tb^III^→Eu^III^ ET. Consequently, in certain cases, despite the occurrence of Tb^III^→Eu^III^ ET, an increase in Tb^III^ concentration may result in longer τ_Tb_ values owing to a more efficient ligand-to-Tb^III^ IET, even though Tb^III^ still acts as an energy donor from the ^5^D_4_ level. Further details regarding Tb^III^→Eu^III^ ET are provided through the analysis of the temperature dependence of the emitting level lifetimes and the numerical calculations.

Changes in the emitting-level lifetimes were also evaluated as a function of the bridge and terminal ligands. For the dppbo-containing compounds, replacing hfa^–^ with tfa^–^ leads to longer τ_Eu_ and τ_Tb_ in the {Tb_0.25_Eu_0.75_}_n_ composition, whereas the opposite trend is observed for the systems with higher Tb^III^ content (Table S5 and Figure 2E). In contrast, for the dppeo-containing compounds, substitution of hfa^–^ by tfa^–^ results in shorter τ_Eu_ and τ_Tb_ in the {Tb_0.25_Eu_0.75_}_n_ composition, while in the {Tb_0.75_Eu_0.25_}_n_ composition, τ_Tb_ lengthens upon replacing hfa^–^ with tfa^–^ (Table S5 and Figure 3E). These variations are likely associated with changes in the triplet-state energies induced by the terminal and bridge ligands, which can alter the ligand-to-Ln^III^ energy-transfer rates and, consequently, the emitting-level lifetimes. In addition, the larger dppbo bridge ligand is expected to promote stronger nonradiative deactivation of the Eu^III 5^D_0_ level through coupling with C–H oscillators,^37^ whereas the higher degree of fluorination in hfa^–^ ligands should reduce nonradiative losses relative to tfa^–^ owing to the replacement of C–H by C–F bonds.^38^ A detailed comparison of the energy-transfer dynamics is provided through computational and numerical analysis, as discussed in the next sections.

**Figure 3:**
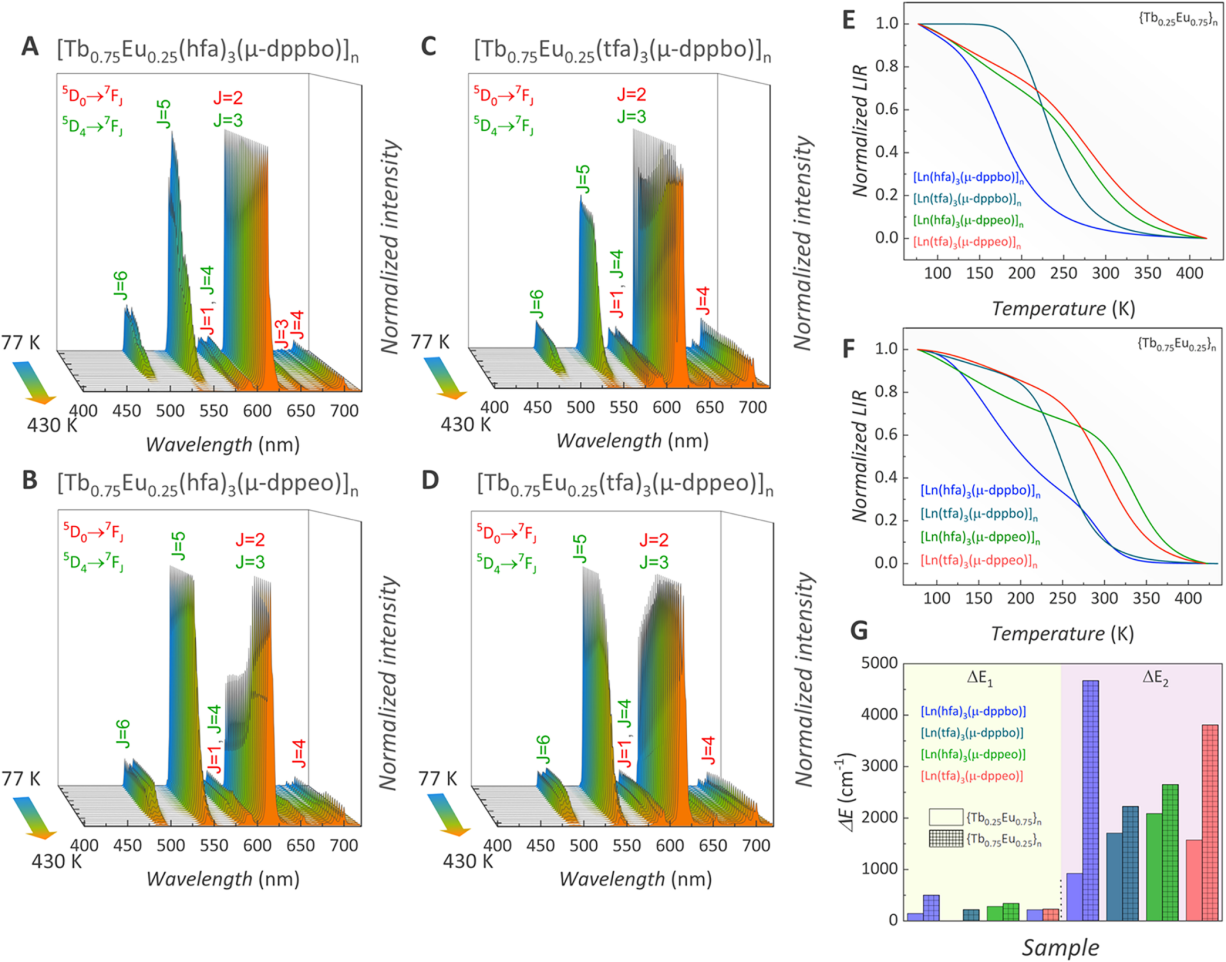
Temperature-dependent (77–430 K) emission spectra (λ_exc_ = 340 nm) for (A) [Tb_0.75_Eu_0.25_(hfa)_3_(μ-dppbo)]_n_, (B) [Tb_0.75_Eu_0.25_(hfa)_3_(μ-dppeo)]_n_, (C) [Tb_0.75_Eu_0.25_(tfa)_3_(μ-dppbo)]_n_, and (D) [Tb_0.75_Eu_0.25_(tfa)_3_(μ-dppeo)]_n_; the emission spectra for the compositions {Tb_0.25_Eu_0.75_}_n_ are shown in Figure S8. Eu^III^ and Tb^III^ transitions are represented in red and green, respectively. Dependence of the normalized luminescence intensity ratio (LIR) on the temperature for the coordination polymers with composition (E) {Tb_0.25_Eu_0.75_} and (F) {Tb_0.75_Eu_0.25_}. (G) Activation energy of thermal quenching processes 1 (AE_1_) and 2 (AE_2_) calculated from the temperature dependence of LIR fitted by eq 1.

The emission quantum yields (Φ_Ln_^L^) of the coordination polymers under 340 nm ligand excitation were evaluated at 300 K (Figure 2F and Table S5). Among the investigated compositions, [Tb_0.25_Eu_0.75_(hfa)_3_(μ-dppbo)]_n_ exhibited the highest emission quantum yield (57.1 ± 6%). With the exception of the [Ln­(tfa)_3_(μ-dppeo)]_n_ samples, the emission quantum yields generally increase with increasing Eu^III^ content. Regarding the influence of the bridging ligand, replacing dppbo with dppeo leads to a modest decrease in the emission quantum yield for the {Tb_0.25_Eu_0.75_}_n_ compositions, whereas a slight increase is observed for the {Tb_0.75_Eu_0.25_}_n_ analogues (Figure 2F and Table S5). On the other hand, changing the terminal ligand from hfa^–^ to tfa^–^ leads to a decrease in the emission quantum yield, except for the [Ln­(tfa)_3_(μ-dppeo)]_n_ compositions (Figure 2F and Table S5).

### Experimental Temperature Dependence of Luminescence

To evaluate the influence of Ln^III^ molar ratio as well as terminal and bridge ligands on the temperature dependence of luminescence of the coordination polymers, temperature-dependent emission spectra upon 340 nm excitation were collected from 77 to 430 K. The emission spectra of the {Tb_0.75_Eu_0.25_}_n_ compositions are shown in Figure 3A–D, while for the {Tb_0.25_Eu_0.75_}_n_ samples, they are presented in Figure S8. For the comparison proposal, the normalized spectra are present in Figures S9 and S10. Across all samples, the band emission intensity associated with the Tb^III 5^D_4_→^7^F_5_ transition shows a pronounced thermal quenching in lower temperature ranges, whereas the Eu^III^ band exhibits an increase in intensity and then a strong suppression in higher temperatures (Figures S11 and S12). For the {Tb_0.25_Eu_0.75_}_n_ compositions, the emission color dependence on the temperature is much less pronounced (Figure S13) than in the {Tb_0.75_Eu_0.25_}_n_ coordination polymers (Figure S14), which contain a higher Tb^III^ content and exhibit a clear emission color shift from yellowish-green to red as the temperature increases.

Upon heating, the emission intensity generally decreases owing to the activation of several thermally assisted quenching mechanisms. These include enhanced nonradiative deactivation through vibrational coupling,^39^ thermally promoted back-IET from the Ln^III^ center to the ligand,^40–42^ or the involvement of ligand-to-metal charge-transfer states.^43,44^ To gain deeper insight into the mechanisms responsible for the thermal quenching, the temperature dependence of τ_Eu_ and τ_Tb_ was investigated over the 77–420 K temperature range for the coordination polymers containing hfa^–^ terminal ligand (Figure 4). As expected, τ_Tb_ of the [Ln­(hfa)_3_(μ-dppeo)]_n_ samples shortens with temperature following an approximately sigmoidal behavior (Figure 4B,D). In contrast, for the [Ln­(hfa)_3_(μ-dppbo)]_n_ compositions, τ_Tb_ remains nearly constant between 77 and 130 K, decreases slightly up to 180 K, remains relatively constant up to 250 K, and then progressively shortens following a sigmoidal trend as the temperature increases to 420 K (Figure 4A,C). In turn, τ_Eu_ remains nearly constant in the low-temperature region, similarly to τ_Tb_, but subsequently becomes longer up to 200 or 300 K for [Ln­(hfa)_3_(μ-dppbo)]_n_ or [Ln­(hfa)_3_(μ-dppeo)]_n_, respectively (Figure 4). These changes are in accordance with the temperature dependence of the Eu^III^ and Tb^III^ emission intensities on the temperature (Figures S11 and S12).

**Figure 4:**
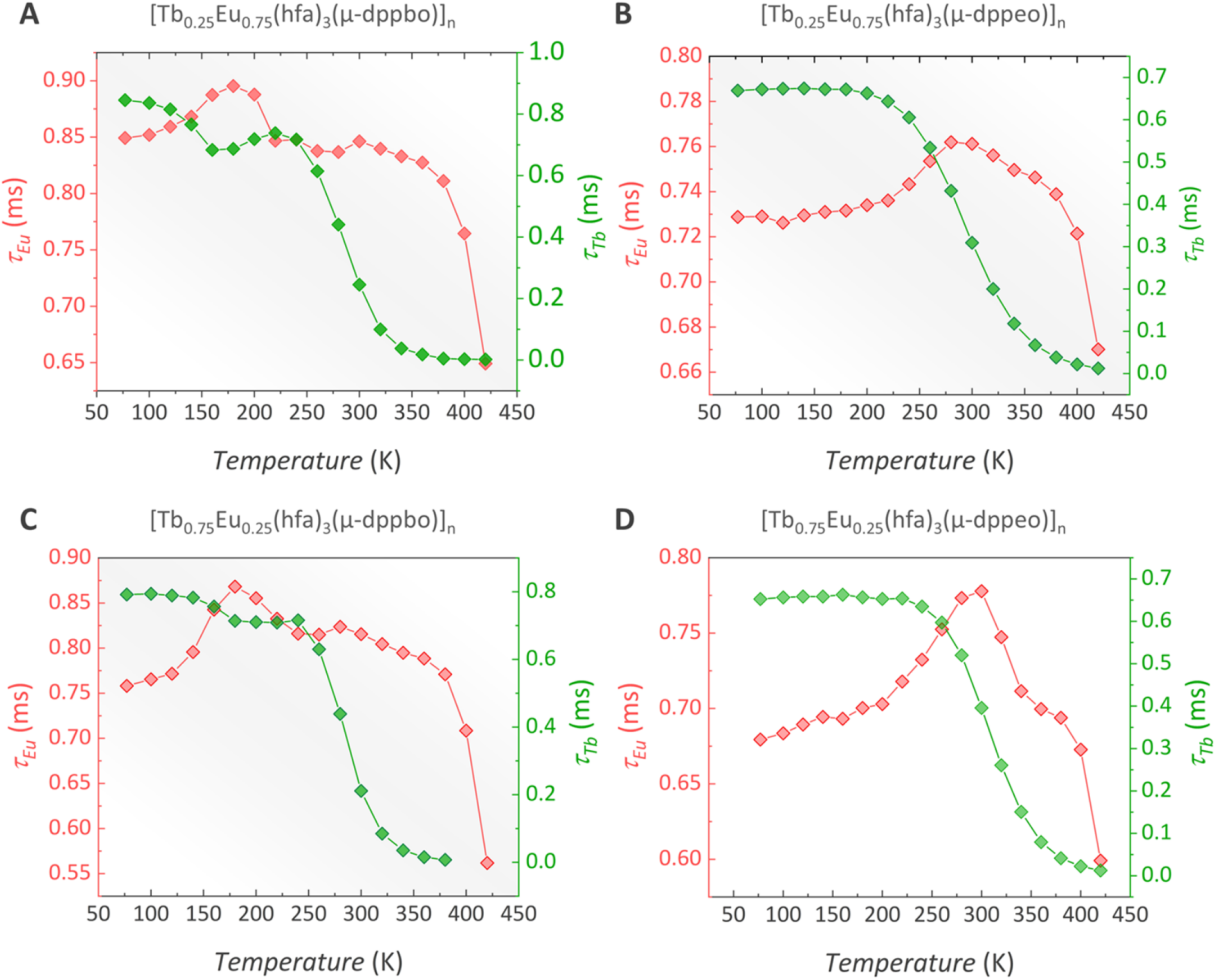
Temperature-dependent (77–420 K) Eu^III 5^D_0_ (τ_Eu_) and Tb^III 5^D_4_ (τ_Tb_) lifetimes for (A) [Tb_0.25_Eu_0.75_(hfa)_3_(μ-dppbo)]_n_, (B) [Tb_0.25_Eu_0.75_(hfa)_3_(μ-dppeo)]_n_, (C) [Tb_0.75_Eu_0.25_(hfa)_3_(μ-dppbo)]_n_, and (D) [Tb_0.75_Eu_0.25_(hfa)_3_(μ-dppeo)]_n_.

The trend observed in the lifetime dependence on the temperature can be attributed to the onset of Tb^III^→Eu^III^ ET activated by temperature. As Tb^III^ acts as a donor and transfers part of its excited-state population to the Eu^III^ acceptor, a shortening in τ_Tb_ is expected, whereas τ_Eu_ tends to get longer, consistent with the trend observed up to 200 K for the [Ln­(hfa)_3_(μ-dppbo)]_n_ samples or 300 K for the [Ln­(hfa)_3_(μ-dppeo)]_n_ compositions.^45^ It is important to note that increasing temperature should also activate nonradiative deactivation pathways, such as back-IET to the ligand states.^42^ This thermally assisted process increases the probability of nonradiative decay, leading to shorter excited-level lifetimes. In this case, the τ_Eu_ and τ_Tb_ shortening observed in the higher temperature regimes indicates that this nonradiative mechanism becomes a dominant contributor to the emitting-level dynamics. The analysis of the temperature dependence of the lifetime therefore suggests that Tb^III^→Eu^III^ ET is more prominent with increasing temperatures up to a critical limit. After this critical temperature, the Ln^III^→ligand back-IET processes become predominant, thereby deactivating the emitting level. These mechanisms are deeply elucidated through calculations of the luminescence dynamics.

To further evaluate the thermal dependence of luminescence in the coordination polymers, the luminescence intensity ratio (LIR = *I*_Tb_/*I*_Eu_) between the bands associated with the Tb^III 5^D_4_ → ^7^F_5_ transition (*I*_Tb_, 535–565 nm) and Eu^III 5^D_0_ → ^7^F_2_ transition (*I*_Eu_, 605–635 nm, with some contribution of overlapped Tb^III 5^D_4_ → ^7^F_3_ band) was plotted as a function of temperature (Figures S15 and S16). As the temperature increases, the LIR starts to decrease, following an almost sigmoidal dependence. This behavior is characteristic of luminescent ratiometric probes built by combining two Ln^III^ emitting centers and can be fitted according to eq 1, which considers the temperature-dependent competition between radiative and nonradiative decays.^46,47^ In eq 1, Δ*E_i_* is the activation energy of each thermal quenching process, α is the ratio between the radiative and nonradiative decay rates of the i-th deactivation channels associated with transitions of intensities *I*_1_ and *I*_2_, *k*_B_ is the Boltzmann constant, and Δ_0_ is the LIR at 0 K.

1 $$\mathrm{LIR}=\frac{\Delta_{0}}{1+\sum_{i}\alpha_{1i}\;\exp\left(-{\Delta E}_{1i}/k_{b}T\right)}$$

The individual fittings of LIR to eq 1 are plotted in Figures S15 and S16, while a comparison between them is provided in Figures 3E,F; the fitting parameters are listed in Table S6. Except for [Tb_0.25_Eu_0.75_(tfa)_3_(μ-dppbo)]_n_, all of the others present at least two thermally induced deactivation processes (Δ*E*_1_ and Δ*E*_2_). This is not surprising considering the different deactivation pathways that have been reported for this kind of material, such as Ln^III^-to-ligand back-IET or Tb^III^-to-Eu^III^ ET.^33,39,40,43^ The activation barrier (Δ*E*, Figure 3G) and ratio among the radiative and nonradiative decay rates (α, Table S6) of both processes tend to increase as the Tb^III^ amount increases. Consequently, the range where the luminescence quenching occurs tends to shift toward higher temperatures, as compared in Figures 3E,F.

The nature of the bridge and terminal ligands also influences the thermal deactivation processes, modulating the activation energy (Figure 3G) and the onset temperature of quenching (Figures 3E,F). This behavior is correlated with differences in crystal packing, singlet and triplet-state energies, and Ln^III^–Ln^III^ distances, which influence the IET dynamics. Moreover, we recently discussed that reducing the energy gap between ^7^F_0_ and ^7^F_1_ levels of Eu^III^ yields a heightened thermal dependency of both ligand-to-Ln^III^ and Tb^III^-to-Eu^III^ ET.^33^ The energy gap between the Eu^III 7^F_0_ and ^7^F_1_ levels depends on the local microsymmetry of the coordination polyhedron. As previously discussed by SC-XRD, the coordination polyhedron of the complexes is assigned to D_2d_ and D_4d_ point groups, where the first belongs to the tetragonal-scalenohedral class,^48^ which splits the ^7^F_1_ level into two components.^49^ On the other hand, the D_4d_ point group belongs to a cubic structural family, which splits the ^7^F_1_ into only one component.^49^ Because of these differences, the energetic barycenter of the ^7^F_1_ level shifts, altering the energy difference between the ^7^F_0_ and ^7^F_1_ levels and, consequently, the ET dynamics.^32,35^ Computational calculations of the ligand-to-Ln^III^ IET and Tb^III^→Eu^III^ ET provide further insights into the mechanisms responsible for the thermal quenching of luminescence, as discussed below.

### Calculations of the Thermal Dependence of Luminescence

The luminescence dynamics of the coordination polymers were investigated through time-dependent density functional theory (TD-DFT) calculations based on the crystal structures obtained from SC-XRD measurements, allowing the determination of the S_1_ and T_1_ excited-state energies. Additional computational details are presented in Supporting Information Note S6. The calculated S_1_ and T_1_ energies (Table S8) for the tfa^–^ complexes are consistent with our previously reported values extracted from the analogous Gd^III^ complexes.^32^ To estimate the ligand-to-Ln^III^ IET rates, parameters such as the distances between the Ln^III^ ions and the centroid of the S_1_ and/or T_1_ states (*R*_*L*_) were determined (Tables S7 and S8), following the procedures described in Supporting Information Notes S6.^50^ The calculations conducted in the JoySpectra^51^ considered both ligand-to-Ln^III^ forward IET pathways from the S_1_ and T_1_ states to the Ln^III 2S+1^L_J_ levels, the Tb^III^-to-Eu^III^ ET, and the corresponding back-ET processes (Figure 5A). The individual contribution of each pathway is shown as an example for [Eu­(tfa)_3_(μ-dppeo)]_n_ in Tables S9–S12.

**Figure 5:**
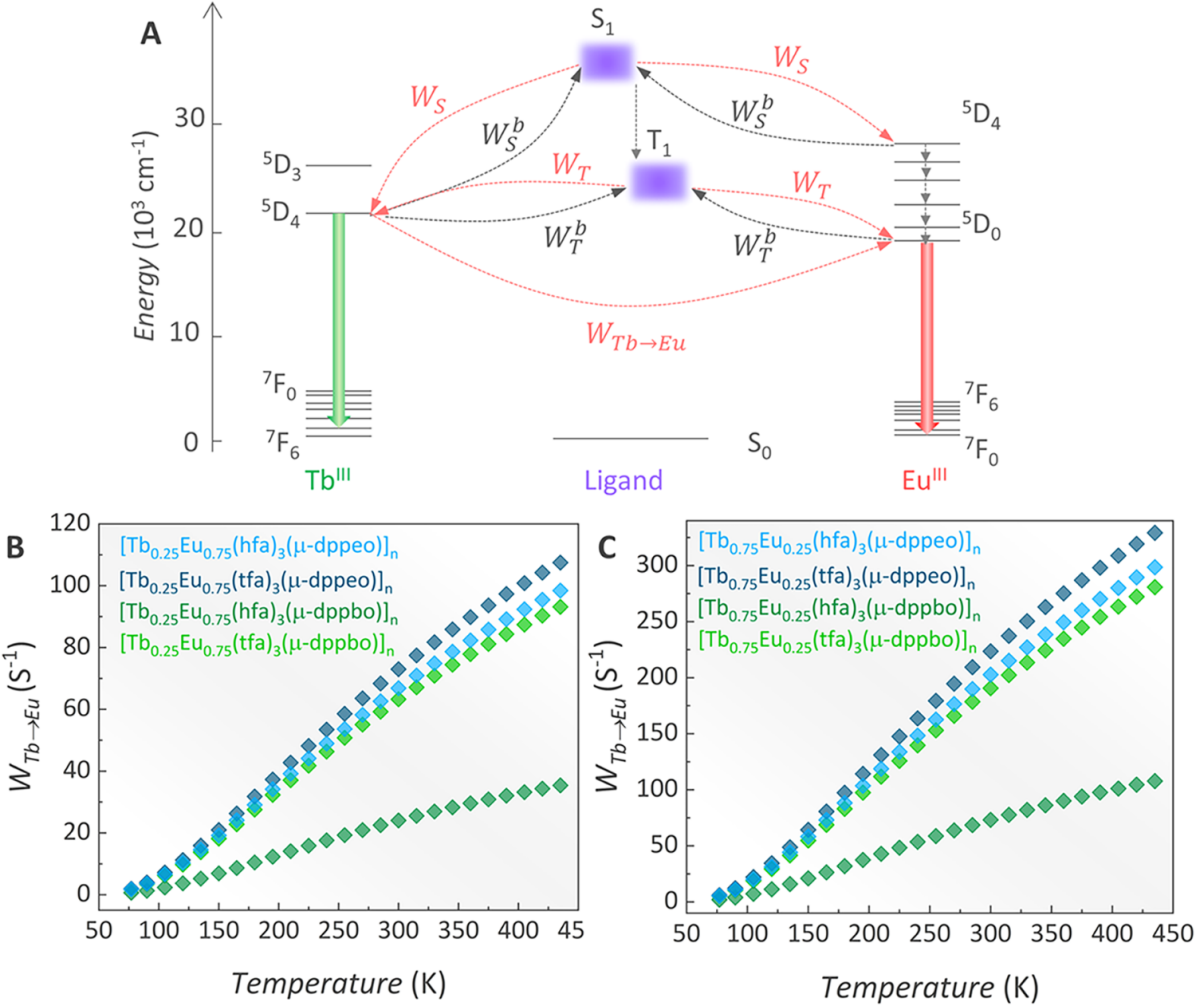
(A) Partial energy diagram highlighting the ligand-centered singlet (S_n_) and triplet (T_1_) states and the Tb^III^ and Eu^III^ levels. The diagram represents the *S_n_* absorption centered in the ligand, ligand-to-Ln^III^ energy transfer, Tb^III^-to-Eu^III^ energy transfer, and Eu^III^ and Tb^III^ emissions. *W*_S_: S_1_→Ln^III^ forward IET rate; *W*_*T*_: T_1_→Ln^III^ forward IET rate; W_S_^b^: Ln^III^→S_1_ back-IET rate; *W*_T_^b^: Ln^III^→T_1_ back-IET; *W*_Tb→Eu_: Tb^III^→Eu^III^ ET rate. Temperature dependence of the Tb^III^→Eu^III^ ET rates for (B) {Tb_0.25_Eu_0.75_}_n_ and (C) {Tb_0.75_Eu_0.25_}_n_ compositions.

At 298 K, the ligand-to-Eu^III^ IET rates for the coordination polymers are on the order of 10^4^–10^5^ s^–1^ from the S_1_ state and 10^8^ s^–1^ from the T_1_ state (Table 1), which indicates that the T_1_ state of the ligands is the main contribution to populate the Eu^III^ excited levels. On the other hand, the ligand-to-Tb^III^ IET rates are on the order of 10^9^ s^–1^ from the S_1_ state and 10^7^ s^–1^ from the T_1_ state, indicating that the S_1_ state of the ligands is the main contribution to populate the Tb^III^ excited levels (Table 1). The nature of the terminal and bridge ligands slightly modulates the IET rates. Replacing tfa^–^ by hfa^–^ systematically decreases both ligand-to-Eu^III^ and ligand-to-Tb^III^ forward IET rates (Table 1), suggesting that the hfa^–^ ligand slightly disfavors ligand-to-Ln^III^ energy transfer. This is due to the slightly larger S_1_ and T_1_ energies as well as a longer distance between the centroid donor and the acceptor (Table S8). Replacing dppeo by dppbo decreases the S_1_→Eu^III^ and T_1_→Tb^III^ IET rates while increasing the T_1_→Eu^III^ and S_1_→Tb^III^ IET rates (Table 1) due to slight shifts in the singlet and triplet energy levels induced by structural modifications.

**Table 1:** Total Rates of IET from the Singlet (S_1_) or Triplet (T_1_) States to Eu^III^ and Tb^III^ as well as Tb^III^-to-Eu^III^ ET at 298.15 K. All rates are in s^–1^ ^a^

	***W***_**S**_ **(s** ^**–1**^ **)**	***W***_**S**_^**b**^ **(s** ^**–1**^ **)**	***W***_**T**_ **(s** ^**–1**^ **)**	***W***_**T**_^**b**^ **(s** ^**–1**^ **)**
**ligand-to-Eu** ^**III**^				
**[Eu(tfa)**_**3**_ **(μ-dppeo)]**_**n**_	2.89 × 10^5^	1.22 × 10^–16^	3.55 × 10^8^	6.96 × 10^7^
**[Eu(hfa)**_**3**_ **(μ-dppeo)]**_**n**_	2.06 × 10^5^	1.71 × 10^–16^	2.54 × 10^8^	9.74 × 10^7^
**[Eu(tfa)**_**3**_ **(μ-dppbo)]**_**n**_	1.28 × 10^4^	1.81 × 10^–17^	4.35 × 10^8^	7.65 × 10^7^
**[Eu(hfa)**_**3**_ **(μ-dppbo)]**_**n**_	9.16 × 10^3^	2.53 × 10^–17^	3.11 × 10^8^	1.07 × 10^8^
**ligand-to-Tb** ^**III**^				
**[Tb(tfa)**_**3**_ **(μ-dppeo)]**_**n**_	4.35 × 10^9^	5.56 × 10^8^	6.50 × 10^7^	2.53 × 10^9^
**[Tb(hfa)**_**3**_ **(μ-dppeo)]**_**n**_	3.10 × 10^9^	7.79 × 10^8^	4.64 × 10^7^	3.54 × 10^9^
**[Tb(tfa)**_**3**_ **(μ-dppbo)]**_**n**_	5.00 × 10^9^	9.05 × 10^8^	4.32 × 10^7^	2.68 × 10^9^
**[Tb(hfa)**_**3**_ **(μ-dppbo)]**_**n**_	3.57 × 10^9^	1.27 × 10^9^	3.09 × 10^7^	3.75 × 10^9^
**Tb** ^**III**^ **-to-Eu** ^**III**^ **(s** ^**–1**^ **)**				
	{Tb_0.25_Eu_0.75_}_n_	{Tb_0.75_Eu_0.25_}_n_		
**[Ln(tfa)**_**3**_ **(μ-dppeo)]**_**n**_	7.365 × 10^1^	2.256 × 10^2^		
**[Ln(hfa)**_**3**_ **(μ-dppeo)]**_**n**_	6.746 × 10^1^	2.045 × 10^2^		
**[Ln(tfa)**_**3**_ **(μ-dppbo)]**_**n**_	6.384 × 10^1^	1.924 × 10^2^		
**[Ln(hfa)**_**3**_ **(μ-dppbo)]**_**n**_	2.426 × 10^1^	7.388 × 10^1^		

a W_S_: S_1_→Ln^III^ forward IET rate; *W*_T_: T_1_→Ln^III^ forward IET rate; *W*_S_^b^: Ln^III^→S_1_ back-IET rate; *W*_T_^b^: Ln^III^→T_1_ back-IET; *W*_Tb→Eu_: Tb^III^→Tb^III^ ET rate.

The back-IET rates from the Ln^III^ ions to the ligand excited states were also evaluated (Table 1). The Eu^III^→S_1_ back-IET rates are markedly lower (10^–16^–10^–16^ s^–1^) than the corresponding S_1_→Eu^III^ forward transfer rates (10^4^–10^5^ s^–1^), indicating a negligible contribution of the singlet-induced back-energy transfer process. In contrast, the Eu^III^→T_1_ back-IET rates are of the same order of magnitude as the respective forward process (10^7^–10^8^ s^–1^), pointing to a non-negligible contribution to the deactivation of the Eu^III^ emitting level. Regarding the Tb^III^-to-ligand back IET, the S_1_→Tb^III^ back-IET rates are of the same order of magnitude as the forward process (10^7^–10^8^ s^–1^), whereas the T_1_→Tb^III^ back-IET rates are considerably faster (10^9^ s^–1^) than the corresponding forward transfer rates (10^7^ s^–1^) (Table 1). Accordingly, at 298 K, the T_1_→Tb^III^ back-IET constitutes an active deactivation pathway for the Tb^III 5^D_4_ level, while the T_1_→Eu^III^ back-IET process contributes to deactivate the Eu^III 5^D_0_ emitting level.

To investigate the influence of temperature on the IET processes, the temperature dependence of the forward and back-IET rates was evaluated, as described in Supporting Information Note S6. The increase in the S_1_→Eu^III^ ET rates with temperature was only marginal for the dppbo compared to the dppeo analogue due to the difference in the S_1_ energy between the two compounds (Figure S17). In contrast, the Eu^III^→S_1_ back-IET rates remain essentially unchanged up to 350 K and then increase (Figure S18). Regarding the T_1_→Eu^III^ pathway, the opposite behavior is observed: the forward IET rates decrease with increasing temperature (Figure S17) while the back-IET rates remain constant (Figure S18). For Tb^III^, both the S_1_→Tb^III^ and T_1_→Tb^III^ forward IET rates increase as the temperature rises from 77 to 420 K, indicating that, although higher temperatures tend to reduce the T_1_→Eu^III^ IET efficiency, it favors the ligand-to-Tb^III^ IET processes (Figure S17). Regarding the back-IET pathways, the Tb^III^ →S_1_ process increases as the temperature rises, while Tb^III^ →T_1_ back-IET rates remain constant (Figure S18).

The Tb^III^→Eu^III^ ET rates, as a function of the relative Tb^III^/Eu^III^ content, were evaluated over the investigated temperature range, as described in the Supporting Information Note S6. At 300 K, the Tb^III^→Eu^III^ ET rates are on the order of 10^1^–10^2^ s^–1^ (Table 1), considerably lower than the ligand→Ln^III^ IET rates (Table 1). This result confirms that ligand-to-Ln^III^ IET constitutes the main sensitization pathway for both Eu^III^ and Tb^III^ luminescence. Nonetheless, for all compositions, the Tb^III^→Eu^III^ ET rates increase as the Tb^III^ content rises from 0.25 to 0.75 mol % (Table 1), confirming that higher Tb^III^ concentrations favor the ET process. Notably, the Tb^III^→Eu^III^ ET rates are relatively similar among samples with the same Ln^III^ contents, with the exception of [Ln­(hfa)_3_(μ-dppbo)_3_]_n_ compositions, which exhibit lower rates. This can be attributed to the larger Tb^III^ ··· Eu^III^ intramolecular distance (10.7704 Å, Figure 1E) combined with the considerable intermolecular distance (11.7562 Å, Figure 1E), both of which reduce the probability of Tb^III^→Eu^III^ ET in [Ln­(hfa)_3_(μ-dppbo)_3_]_n_ relative to the other systems.

As the temperature increases, the Tb^III^→Eu^III^ ET rates increase for all samples, indicating that the process is thermally assisted (Figure 5B,C). This behavior is consistent with the temperature dependence observed for the Eu^III 5^D_0_ and Tb^III 5^D_4_ lifetimes: the ^5^D_4_ lifetime decreases with increasing temperature, while the ^5^D_0_ lifetime lengthens at lower temperatures owing to the enhanced ET contribution (Figure 5B,C). The pathway (^5^D_4_→^7^F_5_)­(^7^F_0_→^5^D_0_) contributes the most for the Tb^III^→Eu^III^ ET (pathway 1021, Table S13). Since this pathway involves the ^7^F_0_ level, its rate is expected to decrease with temperature. Conversely, the (^5^D_4_→^7^F_5_)­(^7^F_1_→^5^D_1_) pathway, which contributes comparably (pathway 1037, Table S13), increases with temperature due to the thermal population of the ^7^F_1_ level. The interplay between these two pathways results in a nonmonotonic temperature dependence to the overall Tb^III^→Eu^III^ ET rate. A clear contrast to the triplet-mediated forward IET is also apparent: the relative contribution of the Tb^III^→Eu^III^ ET grows at higher temperatures, whereas the weight of the T_1_→Eu^III^ sensitization diminishes (Figure S17). Taken together, the temperature dependence of the Tb^III^→Eu^III^ ET rates provides additional evidence that this process is thermally assisted and is directly responsible for both the lengthening of the Eu^III 5^D_0_ lifetime at lower temperatures and the shortening of the ^5^D_4_ lifetime with increasing temperature.

The analysis of the ET rates provides a framework to rationalize and explain the observed trend whereby increasing the Eu^III^ content tends to enhance the emission quantum yield (Figure 2F). Although at 298 K, the S_1_→Tb^III^ IET rates are higher than the S_1_→Eu^III^ IET rates, the Tb^III^→T_1_ back-IET is more pronounced than the corresponding ligand-to-Ln^III^ forward IET (Table 1). Consequently, the stronger back-energy transfer from Tb^III^ to the ligand states contributes to a reduction in the emission quantum yield. Reducing the Tb^III^ content while increasing the Eu^III^ proportion diminishes the overall contribution of the Tb^III^-to-ligand back-IET pathways, favoring Eu^III^ emission and, in turn, enhancing the emission quantum yield. The coordination polymers [Ln­(tfa)_3_(μ-dppeo)_3_]_n_ deviate from this trend, with a higher emission quantum yield observed for {Tb_0.75_Eu_0.25_}_n_ (48.2 ± 6%) compared with {Tb_0.25_Eu_0.75_}_n_ (36.5 ± 4%). The [Ln­(tfa)_3_(μ-dppeo)_3_]_n_ system exhibits the highest S_1_→Eu^III^ and T_1_→Tb^III^ IET rates (Table 1) along with the lowest Eu^III^→S_1_ and Eu^III^→T_1_ back-IET rates, while the [Tb_0.75_Eu_0.25_(tfa)_3_(μ-dppeo)_3_]_n_ composition displays the highest Tb^III^→Eu^III^ ET rates at 300 K (Table 1). In this case, increasing the Tb^III^ content enhances both the T_1_→Tb^III^ IET and the Tb^III^→Eu^III^ ET, thereby boosting the emission quantum yields across the series. It should be noted, however, that a direct comparison of quantum yields among samples containing different terminal and bridge ligands based solely on the ET rates is not feasible, since the emission quantum yield depends not only on the ET dynamics but also on the ligand absorption cross section, which is expected to vary with the ligand framework.

Analysis of the IET rates also provides insight into the trends observed for the emitting-level lifetimes. Although the Tb^III^→Eu^III^ ET process is expected to lengthen the ^5^D_0_ lifetime and shorten the ^5^D_4_ lifetime as the Tb^III^ content increases, its contribution is considerably smaller than that of the ligand-to-Ln^III^ IET rates (Table 1). The population dynamics of the Ln^III^ emitting levels is therefore governed predominantly by the ligand-to-Ln^III^ ET processes rather than by Tb^III^→Eu^III^ ET. For the dppbo-based compositions, the decrease in τ_Tb_ with increasing Tb^III^ content (Figure 2E) is consistent with the enhancement of the Tb^III^→Eu^III^ energy-transfer process. In contrast, for the dppeo-based systems (Figure 2E), the increase in τ_Tb_ suggests that the ligand-to-Tb^III^ ET processes outweigh the Tb^III^→Eu^III^ ET process.

### Luminescence Quenching Mechanism

The combined computational analysis and temperature-dependent steady-state and time-resolved luminescence measurements allow the proposal of a mechanism to explain the thermal behavior of luminescence in the coordination polymers. Upon excitation at 340 nm, the ligands initially populate the S_1_ excited state, followed by intersystem crossing to the T_1_ state (Figure 5A). The sensitization of the Ln^III^ centers then occurs through distinct pathways depending on the emitting ion: Eu^III^ emission is populated predominantly through the T_1_→Eu^III^ IET pathway, whereas Tb^III^ emission is mainly sensitized through the S_1_→Tb^III^ IET process. As temperature increases, the Tb^III^→Eu^III^ ET process becomes more relevant due to thermal population of the Eu^III 7^F_1_ level, which enhances the contribution of quasi-resonant pathways, such as (^5^D_4_→^7^F_5_)­(^7^F_1_→^5^D_1_). This thermally assisted ET event depopulates Tb^III 5^D_4_ level while feeding Eu^III^ excited manifolds, which shortens Tb^III 5^D_4_ lifetime and lengthens the lifetime of Eu^III 5^D_0_ level experimentally up to approximately 200 and 300 K for the dppbo- and dppeo-based compositions, respectively, as shown in Figure 4A,C and 4B,D. The thermal quenching observed in the lower temperature region can therefore be reasonably attributed to the Tb^III^→Eu^III^ ET process, since the activation energy associated with this route (Δ*E*_1_, Figure 3G) closely matches the energy gap between the Tb^III^ and Eu^III^ excited manifolds (Table S13).

At higher temperatures, however, thermally activated back-transfer pathways become dominant. The calculations reveal that the Eu^III^→T_1_ and Tb^III^→T_1_ back-IET rates are comparable to, or even exceed, the corresponding forward ligand-to-Ln^III^ transfer rates (Table 1). These processes lead to progressive shortening of both the Tb^III 5^D_4_ and Eu^III 5^D_0_ lifetimes above the intermediate-temperature regime (Figure 4). The higher activation energy for this process (Δ*E*_2_, Figure 3G) relative to that of the Tb^III^→Eu^III^ ET (Δ*E*_1_) is expected, given the larger energy gap between the ligand S_1_ and T_1_ states and the Ln^III^ excited levels (Tables S10 and S11). Thus, although Tb^III^→Eu^III^ ET dominates the luminescence dynamics at lower temperatures, Ln^III^→ligand back-IET processes become the main deactivation mechanism at higher temperatures, ultimately governing the thermal quenching behavior of the coordination polymers. It is noteworthy that the nature of the bridge and terminal ligands modulates these processes by altering the ligand excited-state energies, Ln^III^–ligand distances, crystal packing, and Ln^III^···Ln^III^ separations.

### Luminescence Thermometry

After evaluating the impacts of the composition on the thermal quenching of luminescence, LIR = *I*_Tb_/*I*_Eu_ was employed as a thermometric parameter to shed light on the thermometric capabilities of the coordination polymers. The dependence of the thermometric parameter on the temperature was used to calculate the relative thermal sensitivity (*S*_r_, Figures S19 and S20) and temperature uncertainty (δT, Figures S19 and S20). Further details regarding the thermometric analysis are shown in the Supporting Information note S1. The temperature range where the coordination polymers operate as a temperature probe is strongly dependent on their composition (Figures 6A,B). This is related to the impact of Ln^III^ amount and ligand scaffold on the ligand-to-Ln^III^ and Tb^III^→Eu^III^ ET dynamics, which affects the activation energy of the quenching process (Figure 3G).

**Figure 6:**
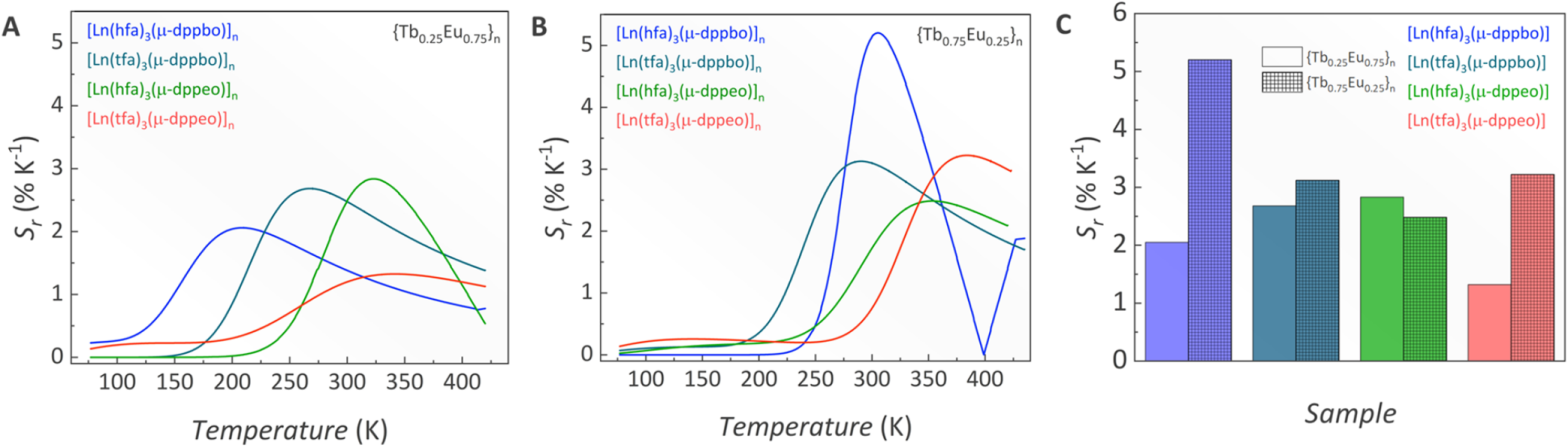
Dependency of the relative thermal sensitivity on the temperature for the coordination polymers with composition (A) {Tb_0.25_Eu_0.75_} and (B) {Tb_0.75_Eu_0.25_}. (C) Maximum relative thermal sensitivity (*S*_m_) calculated for the coordination polymers.

The maximum relative thermal sensitivity (*S*_m_) depends on the composition, as it directly impacts the IET and the thermal quenching of luminescence. Interestingly, the coordination polymers with larger Tb^III^ content tend to present larger *S*_m_ (Figure 6C) in accordance with the higher activation barrier to the quenching process (Figure 3G) and α value (Table S6) as well as more prominent Tb^III^-to-Eu^III^ ET (Figure 5B,C). Among the investigated samples, [Tb_0.75_Eu_0.25_(hfa)_3_(μ-dppbo)]_n_ exhibits the highest *S*_m_, reaching 5.20% K^–1^ at 305 K. This compound presents the largest activation barrier for thermal quenching (4670 cm^–1^, Figure 3G), along with the highest ratio of radiative to nonradiative decay rates (2.69 × 10^10^, Table S6). It also displays the lowest Tb^III^→Eu^III^ ET rates (Figure 5C), coupled with the highest Tb^III^→S_1_ back-IET rates and the most pronounced temperature dependence among all ET pathways, while the remaining ones are comparatively less temperature dependent (Figure S18).

Notably, although the Tb^III^→Eu^III^ ET is favored with increasing temperature, the contribution of the inter-Ln^III^ ET pathway remains dominant only up to a threshold temperature (ca. 200 and 300 K for the dppbo- and dppeo-based compositions, respectively, Figure 4). On the other hand, at higher temperatures, back-IET to the ligand excited states becomes predominant. Below 200 K, the relative thermal sensitivity remains low, particularly for the {Tb_0.75_Eu_0.25_}_n_ compositions (Figure 6A,B). This behavior indicates that the thermal sensitivity is predominantly governed by back-IET processes involving the ligand excited states, as this mechanism becomes significant in the temperature range where the materials exhibit higher *S*_r_ values (above 200 K).

Overall, a general tendency toward higher S_m_ values is observed with increasing Tb^III^ content, except for the [Ln­(hfa)_3_(μ-dppeo)]_n_ composition. This particular system, which deviates from the overall trend, exhibited negligible temperature dependence of the Eu^III^→T_1_ and Tb^III^→T_1_ back-energy-transfer rates, together with only moderate temperature dependence of the Eu^III^→S_1_ and Tb^III^→S_1_ processes (Figure S18). These results suggest that increasing the concentration of the luminescence sensitizer is a promising strategy to enhance the relative thermal sensitivity for most of the coordination polymers.

For the dppbo-based systems, increasing the Tb^III^ content from {Tb_0.25_Eu_0.75_}_n_ to {Tb_0.75_Eu_0.25_}_n_ significantly enhances the maximum relative thermal sensitivity, raising it from 2.05 to 5.20% K^–1^ (Figure 6C). In contrast, for the dppeo-based systems, the relative sensitivities remain nearly unchanged despite variations in the Tb^III^/Eu^III^ ratio (Figure 6C). This behavior is linked with the higher backward-IET rates and the stronger temperature dependence of the Tb^III^→S_1_ back-IET process in the dppbo-based systems (Figure S18B), whereas the remaining pathways are either nearly temperature independent or contribute only negligibly (Figure S18A,C,D). These differences, which are modulated by the nature of the bridge and terminal ligands, originate from variations in the ligand excited-state energies, Ln^III^–ligand distances, and crystal packing.

The *S*_m_ was used as a figure of merit to compare the performance of the luminescent temperature probes (Table 2). The *S*_m_ values are average compared with those reported for other coordination polymers and metal–organic frameworks combining Eu^III^ and Tb^III^. The temperature uncertainties were calculated from eq S2 (Supporting Information note S1), achieving values lower than 0.1 K within the temperature range where the systems operate as a temperature probe (Figures S19 and S20). These low values are expected considering that a highly sensitive photomultiplier module (PMT) was employed as a detector in the measurements.^52^ Finally, the reproducibility of the thermometric response based on the LIR was confirmed over the full temperature range by performing 10 successive heating–cooling cycles for the [Tb_0.75_Eu_0.25_(hfa)_3_(μ-dppbo)]_n_ sample (Figure S21).

**Table 2:** Maximum Relative Thermal Sensitivity (*S*_m_) and Temperature Uncertainty (δT) Achieved at the Temperature *T*_m_ for Selected Coordination Polymers Combining Eu^III^ and Tb^III^ ^a^

**sample**	**monitored temperature range (K)**	**S**_**r**_ **(% K** ^**–1**^ **)**	**δT (K)**	**T**_**m**_ **(K)**	**refs**
[Tb_0.25_Eu_0.75_(hfa)_3_(μ-dppbo)]_n_	125–430	2.05 ± 0.1	0.01	208	^b^
[Tb_0.75_Eu_0.25_(hfa)_3_(μ-dppbo)]_n_	250–400	5.20 ± 0.1	0.01	305	^b^
[Tb_0.25_Eu_0.75_(tfa)_3_(μ-dppbo)]_n_	180–430	2.68 ± 0.1	0.01	268	^b^
[Tb_0.75_Eu_0.25_(tfa)_3_(μ-dppbo)]_n_	210–430	3.12 ± 0.1	0.01	291	^b^
[Tb_0.25_Eu_0.75_(hfa)_3_(μ-dppeo)]_n_	220–400	2.83 ± 0.1	0.01	323	^b^
[Tb_0.75_Eu_0.25_(hfa)_3_(μ-dppeo)]_n_	250–400	2.48 ± 0.1	0.06	353	^b^
[Tb_0.25_Eu_0.75_(tfa)_3_(μ-dppeo)]_n_	250–430	1.32 ± 0.1	0.02	340	^b^
[Tb_0.75_Eu_0.25_(tfa)_3_(μ-dppeo)]_n_	300–430	3.22 ± 0.1	0.01	383	^b^
[Me_2_NH_2_][LnL^1^(H_2_O)_2_] (Ln = Eu, Tb, Gd)	77–450	6.1		430	^53^
Tb_0.914_Eu_0.086_-PDA	10–325	5.96	0.02	25	^47^
d-U(600)-Eu_0.25_Tb_0.75_(btfa)_3_(bpeta)	10–330	4.9		150	^54^
Tb_0.99_Eu_0.01_-BDC-DSTP	77–200	3.9		200	^55^
[Eu_0.102_Tb_0.898_(notpH_4_)(NO_3_)(H_2_O)]	18–300	3.9	0.15	38	^56^
Tb_0.9_Eu_0.1_-PIA	100–300	3.27		300	^57^
[Tb_0.088_Eu_0.2188_(phen)(1,3,5-btc)(dmf)]	293–393	2.71	0.5	298	^58^
[Tb_0.99_Eu_0.01_(hfa)_3_(dppb)]_n_	100–450	0.83			^28^
[Tb_0.99_Eu_0.01_(hfa)_3_(dpb)]_n_	100–450	0.82			^28^
[Tb_0.99_Eu_0.01_(hfa)_3_(dppcz)]_n_	100–450	0.45			^28^
[Tb_0.90_Eu_0.1_(1,3-bdc)_3_(H_2_O)_2_]·H_2_O	12–101	3.26	0.07	35.5	^59^
Eu_*x*_Tb_1_@x-BABDC	80–240	3.61	0.50	240	^60^

a The monitored temperature range for each assay is also shown.

b This work. phen: phenantroline; H_4_L^1^: 2,6-di­(20,40-dicarboxylphenyl)­pyridine; 1,3-H2bdc: 1,3-benzene-dicarboxylic acid; H^2^BABDC: 2,5-bis­(allyloxy)­terephthalic acid; dpbp: 4,4′-bis­(diphenylphosphoryl) bipheny; dppcz: 3,6-bis­(diphenylphosphoryl)-9-phenylcarbazole; PDA: 1,4-phenylenediacetic acid; HPIA: 5-(pyridin-4-yl)­isophthalic acid; BDC: 1,4-benzene dicarboxylic acid; H_2_DSTP: 2,4-(2,2′:6′,2″-terpyridin-4′-yl)-benzenedisulfonic acid; bpeta: 1,2-bis­(4-pyridyl)­ethane; Hbtfa: 4,4,4-trifluoro-1-phenyl-1,3-butanedione; notpH_6_: 1,4,7-triazacyclononane-1,4,7-triyl-tris­(methylenephosphonic acid).

In this context, this study demonstrates that in ratiometric luminescent temperature probes based on Eu^III^ and Tb^III^ 1D-coordination polymers, both maximum relative thermal sensitivity and operating range can be tuned by varying the phosphine oxide bridge ligand, the β-diketone terminal ligand, and the Ln^III^ molar proportion. Tb^III^→Eu^III^ ET is thermally assisted and contributes significantly to the temperature dependence of the luminescence intensity ratio at lower temperatures (up to 200 and 300 K for the dppbo- and dppeo-based compositions, respectively), whereas ligand-mediated back-IET processes become dominant at higher temperatures and control the thermal quenching behavior. Moreover, increasing the Tb^III^ content enhances Tb^III^-to-Eu^III^ ET, leading to a larger activation barrier for thermal luminescence quenching and boosting the relative thermal sensitivity.

## Conclusions

Herein, we investigated how ligand architecture and lanthanide­(III) molar ratio govern the luminescent thermometric performance of a family of Eu^III^/Tb^III^-based coordination polymers employing dppeo or dppbo as a bridge ligand and tfa^–^ or hfa^–^ as a terminal ligand. The coordination polymers adopt almost linear one-dimensional chain structures, except for the [Ln­(tfa)_3_(μ-dppbo)]_n_ composition, which exhibits a zigzag conformation. This leads to a coordination environment described by a pseudo-D_2d_ point group for [Ln­(tfa)_3_(μ-dppeo)]_n_ and shorter intermolecular Ln^III^ ··· Ln^III^ distances. In contrast, the LnO_8_ polyhedra in the other compounds are better described by a pseudo-D_4d_ symmetry. Steady-state and time-resolved luminescence spectroscopy, combined with computational calculations, confirm that the ligand-to-Ln^III^ ET governs the luminescence dynamics of the coordination polymers. Yet, higher Tb^III^ content leads to more pronounced Tb^III^-to-Eu^III^ energy transfer. Temperature-dependent ratiometric luminescence based on the Tb^III 5^D_4_ → ^7^F_5_ and Eu^III 5^D_0_ → ^7^F_2_ emissions revealed that at least two pathways control the thermal deactivation of luminescence: (*i*) Tb^III^→Eu^III^ ET, which contributes the most up to 200 and 300 K for the dppbo- and dppeo-based compositions, respectively, and (*ii*) Ln^III^-to-ligand back-IET processes, which are dominant at higher temperatures, thereby controlling the thermometric response. As the Tb^III^ loading increases, the coordination polymers tend to exhibit larger activation barriers for the thermal quenching of luminescence. As a consequence, the maximum relative thermal sensitivity of luminescence tends to enhance as the Tb^III^ amount increases, reaching a maximum value of 5.20% K^–1^ at 305 K for [Tb_0.75_Eu_0.25_(hfa)_3_(μ-dppbo)]_n_. This study demonstrates that higher Tb^III^ contents, combined with stronger temperature dependence of the ligand-to-Ln^III^ back-IET rates, increase the activation barrier for thermal quenching of luminescence, ultimately leading to enhanced thermal sensitivity. These findings offer valuable design strategies for the development of Eu^III^/Tb^III^-based luminescent thermometers with tunableperformance.

## Supplementary Material







## Notes

Synthetic procedure and characterization apparatus, additional structural data, and further steady-state and time-resolved photoluminescence spectroscopy data (PDF)
20230814FS02_auto (CIF)
20230814FS01_auto (CIF)

## References

[1] Brites C. D. S., Fuertes M. C., Angelomé P. C., Martínez E. D., Lima P. P., G Soler-Illia J. A. A., Carlos L. D. (2017). Tethering Luminescent
Thermometry and Plasmonics: Light Manipulation to Assess Real-Time
Thermal Flow in Nanoarchitectures. Nano Lett..

[2] Rodrıguez-Sevilla P., Marin R., Ximendes E., del Rosal B., Benayas A., Jaque D. (2022). Luminescence
Thermometry for Brain
Activity Monitoring: A Perspective. Front. Chem..

[3] Bednarkiewicz A., Drabik J., Trejgis K., Jaque D., Ximendes E., Marciniak L. (2021). Luminescence
based temperature bio-imaging: Status,
challenges, and perspectives. Appl. Phys. Rev..

[4] Vogel R., Groefsema D. W., van den Bulk M. A., Jacobs T. S., Prins P. T., Rabouw F. T., Weckhuysen B. M. (2025). Operando Luminescence Thermometry
for Hydrocarbon Conversion Catalysis: Dealing with Dynamic Changes
in Catalyst Optical Properties. ACS Appl. Mater.
Interfaces.

[5] Okabe K., Inada N., Gota C., Harada Y., Funatsu T., Uchiyama S. (2012). Intracellular temperature mapping with a fluorescent
polymeric thermometer and fluorescence lifetime imaging microscopy. Nat. Commun..

[6] Aigouy L., Tessier G., Mortier M., Charlot B. (2005). Scanning thermal imaging
of microelectronic circuits with a fluorescent nanoprobe. Appl. Phys. Lett..

[7] Samson B., Aigouy L., Löw P., Bergaud C., Kim B. J., Mortier M. (2008). ac thermal imaging
of nanoheaters using a scanning
fluorescent probe. Appl. Phys. Lett..

[8] Xiong J., Zhao M., Han X., Cao Z., Wei X., Chen Y., Duan C., Yin M. (2017). Real-time micro-scale
temperature imaging at low cost based on fluorescent intensity ratio. Sci. Rep..

[9] Bednarkiewicz A., Marciniak L., Carlos L. D., Jaque D. (2020). Standardizing luminescence
nanothermometry for biomedical applications. Nanoscale.

[10] Brites C.
D. S., Lima P. P., Silva N. J. O., Millan A., Amaral V. S., Palacio F., Carlos L. D. (2012). Thermometry at the nanoscale. Nanoscale.

[11] Suta M. (2025). What makes
β-NaYF_4_:Er^3+^,Yb^3+^ such a successful
luminescent thermometer?. Nanoscale.

[12] Shi R., Martinez E. D., Brites C. D. S., Carlos L. D. (2021). Thermal enhancement
of upconversion emission in nanocrystals: a comprehensive summary. Phys. Chem. Chem. Phys..

[13] Rao X., Song T., Gao J., Cui Y., Yang Y., Wu C., Chen B., Qian G. (2013). A highly sensitive mixed lanthanide
metal-organic framework self-calibrated luminescent thermometer. J. Am. Chem. Soc..

[14] Suta M., Meijerink A., Theoretical A. (2020). Framework for Ratiometric Single
Ion Luminescent Thermometers—Thermodynamic and Kinetic Guidelines
for Optimized Performance. Adv. Theory Simul..

[15] Hasegawa Y., Kitagawa Y. (2022). Luminescent lanthanide coordination
polymers with transformative
energy transfer processes for physical and chemical sensing applications. J. Photochem. Photobiol. C, Photochem. Rev..

[16] Wang L., He Q.-Q., Gao Q., Xu H., Zheng T.-F., Zhu Z.-H., Peng Y., Chen J.-L., Liu S.-J., Wen H.-R. (2023). Controllable Synthesis of Tb^III^ Metal–Organic
Frameworks with Reversible Luminescence Sensing for Benzaldehyde Vapor. Inorg. Chem..

[17] Wang K., Zhu Y.-L., Zheng T.-F., Xie X., Chen J.-L., Wu Y.-Q., Liu S.-J., Wen H.-R. (2023). Highly
pH-Responsive
Sensor Based on a Eu^III^ Metal–Organic Framework
with Efficient Recognition of Arginine and Lysine in Living Cells. Anal. Chem..

[18] Huang J., Li Z.-Y., Pan S., Zheng T.-F., Wu Y., Wen H.-R., Liu S.-J. (2026). A Stable Eu^III^ Metal–Organic
Framework Sensor for the Fluorescence Detection of Tryptophan and
Cancer Biomarker in Living Cells. Inorg. Chem..

[19] Tang H., Cheng S., Zhang Z., He M., Qian J., Li L. (2024). Tailoring Energy Transfer in Mixed
Eu/Tb Metal–Organic Frameworks
for Ratiometric Temperature Sensing. Molecules.

[20] Ivanova A. A., Polikovskiy T. A., Gontcharenko V. E., Korshunov V. M., Kiskin M. A., Taydakov I. V., Belousov Y. A. (2024). Precision across
temperatures: Eu/Tb luminescent thermometer with exceptionally high
and stable sensitivity from 180 to 320 K. Sens. Actuators, A.

[21] Bünzli J.-C., Piguet C. (2005). Taking advantage of luminescent lanthanide
ions. Chem. Soc. Rev..

[22] Malta O. L. (2008). Mechanisms
of non-radiative energy transfer involving lanthanide ions revisited. Non. Cryst. Solids.

[23] Kharcheva A. V., Bozhko A. A., Sokolovskaya Y. G., Borisova N. E., Ivanov A. V., Patsaeva S. V. (2023). Bimetallic Eu/Tb
Complexes for Ratiometric Temperature
Sensing with Unusual Enhancement of Eu Luminescence with. Temperature. Photonics.

[24] Yang H., He R., Liu S., Song W., Zhao X., Yang F., Yuan H., Wang Y. (2025). Multi-Emitting
Ratiometric Temperature
Sensing and Tunable White Light Emitting Based on Effective Energy
Transfer in a Lanthanide-Brønsted Acidic Ionic Liquid Coordination
Polymer. Inorg. Chem..

[25] Peng X.-W., Liu Q.-Y., Wang H.-H., Wang Y.-L. (2019). Eu­(III)- and Tb­(III)-coordination
polymer luminescent thermometers constructed from a π-rich aromatic
ligand exhibiting a high sensitivity. Dyes Pigm..

[26] Cui Y., Zou W., Song R., Yu J., Zhang W., Yang Y., Qian G. (2014). A ratiometric and colorimetric
luminescent thermometer over a wide
temperature range based on a lanthanide coordination polymer. Chem. Commun..

[27] Bellucci L., Bottaro G., Labella L., Marchetti F., Samaritani S., Dell’Amico D.
B., Armelao L. (2021). 1D-Zigzag
Eu^3+^/Tb^3+^ Coordination Chains as Luminescent
Ratiometric Thermometers Endowed with Multicolor Emission. Materials.

[28] Hatanaka M., Hirai Y., Kitagawa Y., Nakanishi T., Hasegawa Y., Morokuma K. (2017). Organic linkers control
the thermosensitivity
of the emission intensities from Tb­(III) and Eu­(III) in a chameleon
polymer. Chem. Sci..

[29] Hirai Y., Nakanishi T., Kitagawa Y., Fushimi K., Seki T., Ito H., Hasegawa Y. (2016). Luminescent Europium­(III) Coordination Zippers Linked
with Thiophene-Based Bridges. Angew. Chem.,
Int. Ed..

[30] Miyata K., Konno Y., Nakanishi T., Kobayashi A., Kato M., Fushimi K., Hasegawa Y. (2013). hameleon Luminophore
for Sensing Temperatures: Control of Metal-to-Metal and Energy Back
Transfer in Lanthanide Coordination Polymers. Angew. Chem., Int. Ed..

[31] Kitagawa Y., Kumagai M., da Rosa P. P. F., Fushimi K., Hasegawa Y. (2021). Long-Range
LMCT Coupling in Eu^III^ Coordination Polymers for an Effective
Molecular Luminescent Thermometer. Chem. - Eur.
J..

[32] A
Lima D., Bispo-Jr A. G., Galico D. A., Coelho S. F. N., Araujo
Neto J. H., Ellena J. A., Petiote L., Mazali I. O., Sigoli F. A. (2023). Tuning the thermometric features in 1D luminescent
Eu^III^ and Tb^III^ coordination polymers through
different bridge phosphine oxide ligands. Inorg.
Chem..

[33] Bispo-Jr A. G., Saraiva L. F., Lima S. A. M., Pires A. M., Mazali I. O., Sigoli F. A. (2025). Lanthanide coordination
polymers as luminescent thermometers:
integrating theoretical modeling with experimental analysis to tune
the thermal response. J. Mater. Chem. C.

[34] Bispo A. G., Saraiva L. F., Carneiro Neto A. N., Coelho S. F. N., Mazali I. O., Carlos L. D., Sigoli F. A. (2025). Insights
into quantum cutting and downshifting contributions to near-infrared
Yb^III^ luminescence in 1D coordination polymers. J. Mater. Chem. C.

[35] de
Andrade S. A., Bispo-Jr A. G., Simoni D. A., Ellena J., de Araujo Neto J. H., Mazali I. O., Sigoli F. A. (2025). The role of terminal
and bridge ligands in the molecular upconversion of lanthanide­(III)
1D coordination polymers. J. Mater. Chem. C.

[36] Topor A., Avram D., Dascalu R., Maxim C., Tiseanu C., Andruh M. (2021). Luminescence thermometry based on one-dimensional benzoato-bridged
coordination polymers containing lanthanide ions. Dalton Trans..

[37] Beeby A., Clarkson I. M., Dickins R. S., Faulkner S., Parker D., Royle L., de Sousa A. S., Williams J. A. G., Woods M. (1999). Non-radiative
deactivation of the excited states of europium, terbium and ytterbium
complexes by proximate energy-matched OH, NH and CH oscillators: an
improved luminescence method for establishing solution hydration states. J. Chem. Soc., Perkin Trans..

[38] Ning Y., Tang J., Liu Y.-W., Jing J., Sun Y., Zhang J.-L. (2018). Highly luminescent,
biocompatible ytterbium­(III) complexes
as nearinfrared fluorophores for living cell imaging. Chem. Sci..

[39] Eliseeva S. V., Bunzli J-C. G. (2010). Lanthanide luminescence for functional materials and
bio-sciences. Chem. Soc. Rev..

[40] Charbonnière L.
J., Balsiger C., Schenk K. J., Bünzli J.-C. (1998). Complexes
of p-tert-butylcalix[5]­arene with lanthanides: synthesis, structure
and photophysical properties. J. Chem. Soc.,
Dalton Trans..

[41] Bellucci L., Bottaro G., Labella L., Causin V., Marchetti F., Samaritani S., Dell’Amico D.
B., Armelao L. (2020). Composition-Thermometric
Properties Correlations in Homodinuclear Eu3+ Luminescent Complexes. Inorg. Chem..

[42] Canisares, F. S. M. ; Laurindo, M. G. ; Saraiva, L. F. ; Araujo-Neto, J. H. ; Ellena, J. ; Brito, H. F. ; Bispo-Jr, A. G. Elucidating thermally activated luminescence quenching in Eu^III^ β-diketonate complexes as crystals or polymeric films for application in thermometry J. Mater. Chem. C. 2026, Advance article.

[43] Carlotto A., Babetto L., Carlotto S., Miozzi M., Seraglia R., Casarin M., Bottaro G., Rancan M., Armelao L. (2020). Luminescent
Thermometers: From a Library of Europium­(III) β-Diketonates
to a General Model for Predicting the Thermometric Behaviour of Europium-Based
Coordination Systems. ChemPhotoChem..

[44] Berry M. T., May P. S., Xu H. (1996). Temperature
Dependence of the Eu^3+5^D_0_ Lifetime in Europium
Tris­(2,2,6,6-tetramethyl-3,5-heptanedionato). J. Phys. Chem. A.

[45] Tanner P. A., Zhou L., Duan C., Won K.-L. (2018). Misconceptions in
electronic energy transfer: bridging the gap between chemistry and
physics. Chem. Soc. Rev..

[46] Ananias D., Almeida Paz F. A., Yufit D. S., Carlos L. D., Rocha J. (2015). Photoluminescent
Thermometer Based on a Phase-Transition Lanthanide Silicate with Unusual
Structural Disorder. J. Am. Chem. Soc..

[47] Wang Z., Ananias D., Carné-Sánchez A., Brites C. D. S., Imaz I., Maspoch D., Rocha J., Carlos L. D. (2015). Lanthanide–Organic Framework Nanothermometers
Prepared by Spray-Drying. Adv. Funct. Mater..

[48] Duffey G. H. (1950). Tetragonal
Antiprism Bond Orbitals. J. Chem. Phys..

[49] Thor W., Carneiro Neto A. N., Moura R. T., Wong K.-L., Tanner P. A. (2024). Europium­(III) coordination chemistry: structure, spectra
and hypersensitivity. Coord. Chem. Rev..

[50] Moura J. L., Costa I. F., Santos P. R. S., Silva I. F., Moura R. T., Carneiro
Neto A. N., Faustino W. M., Brito H. F., Sabino J. R., Teotonio E. E. S. (2022). Enhancing the Luminescence of Eu­(III)
Complexes with the Ruthenocene Organometallic Unit as Ancillary Ligand. Inorg. Chem..

[51] Moura R. T., Carneiro Neto A. N., Aguiar E. C., Santos-Jr C. V., de Lima E. M., Faustino W. M., Teotonio E. E. S., Felinto H. B., Felinto M. C. F. C., Ferreira R. A. S., Carlos L. D., Longo R. L., Malta O. L. (2021). JOYSpectra:
A web platform for luminescence of lanthanides. Opt. Mater. X.

[52] Brites, C. D. S. ; Millán, A. ; Carlos, L. D. Handbook on the Physics And Chemistry of Rare Earths; Elsevier, 2016; Vol. 49, p 339.

[53] Yang Y., Chen L., Jiang F., Yu M., Wan X., Zhang B., Hong M. (2017). A family of doped lanthanide
metal–organic
frameworks for wide-range temperature sensing and tunable white light
emission. J. Mater. Chem. C.

[54] Brites C. D. S., Lima P. P., Carlos L. D. (2016). Tuning
the sensitivity of Ln^3+^-based luminescent molecular thermometers
through ligand
desig. J. Lumin..

[55] Wei Y. Q., Sa R. J., Li Q. H., Wu K. C. (2015). Highly stable and
sensitive LnMOF ratiometric thermometers constructed with mixed ligands. Dalton Trans..

[56] Ren M., Brites C. D. S., Bao S. S., Ferreira R. A. S., Zheng L. M., Carlos L. D. (2015). A cryogenic luminescent ratiometric thermometer based
on a lanthanide phosphonate dimer. J. Mater.
Chem. C.

[57] Rao X. T., Song T., Gao J. K., Cui Y. J., Yang Y., Wu C. D., Chen B. L., Qian G. D. (2013). A Highly Sensitive
Mixed Lanthanide Metal–Organic Framework Self-Calibrated Luminescent
Thermometer. J. Am. Chem. Soc..

[58] Zaręba J. K., Nyk M., Janczak J., Samoc M. (2019). Three-Photon Absorption of Coordination
Polymer Transforms UV-to-VIS Thermometry into NIR-to-VIS Thermometry. ACS Appl. Mater. Interfaces.

[59] N’Dala-Louika I., Ananias D., Latouche C., Dessapt R., Carlos L. D., Serier-Brault H. (2017). Ratiometric
mixed Eu–Tb metal–organic
framework as a new cryogenic luminescent thermometer. J. Mater. Chem. C.

[60] Feng T., Ye Y., Liu X., Cui H., Li Z., Zhang Y., Liang B., Li H., Chen B. (2020). A Robust Mixed-Lanthanide
PolyMOF Membrane for Ratiometric Temperature Sensing. Angew. Chem., Int. Ed..

