# Supplementary material for: Modulating Luminescence Thermometry of the EuIII/TbIII Pair in Coordination Polymers through Ligand Structure and Lanthanide(III) Molar Ratio
Source: ACS Omega. 2026 Jul 10;11(29):43849–62. doi: 10.1021/acsomega.6c03259 (PMC13425478; doi:10.1021/acsomega.6c03259)
Supplement: Supplementary file 1 [file ao6c03259_si_001.pdf]

Modulating Luminescence Thermometry of the Eu^{III}/Tb^{III} pair in Coordination Polymers through Ligand Structure and Lanthanide(III) Molar Ratio

Gabriel J. S. Araujo,[a] Talita C. Souza,[a] Stefano A. de Andrade,[a] Jéssica F. Rodrigues,[a] Sergio F. N. Coelho,[a] Pedro H. O. Santiago,[b] Javier Ellena,[b] Italo O. Mazali,[a] Leonardo F. Saraiva,[c] Airton G. Bispo-Jr,[d] * Fernando A. Sigoli[a] **

[a] Institute of Chemistry, State University of Campinas, Campinas, São Paulo 13083-970, Brazil.

[b] Institute of Physics, University of São Paulo, São Carlos, São Paulo 13566-590, Brazil.

[c] Department of Chemistry and Biochemistry, São Paulo State University (UNESP), School of Science and Technology, São Paulo, SP 19060-900, Brazil.

[d] Institute of Chemistry, University of São Paulo, São Paulo, São Paulo 05508-000, Brazil.

* airton.bispo.junior@iq.usp.br

** fsigoli@unicamp.br

Supplementary note S1 – Synthesis and characterization 2

Supplementary note S2 – Structural data 4

Supplementary note S3 – Steady-state photoluminescence 7

Supplementary note S4 – Time-resolved photoluminescence 9

Supplementary note S5 – Temperature-dependent photoluminescence data 12

Supplementary note S6 – Computational calculations 22

Supplementary note S7 – Luminescence thermometry 34

Supplementary references 37

Supplementary note S1 – Synthesis and characterization

Synthesis of the coordination polymers

The synthesis of the 1D coordination polymers was previously described by us elsewhere.[1,2] The $[Ln(tfa)_3(\mu\text{-dppeo})]_n$, $[Ln(hfa)_3(\mu\text{-dppeo})]_n$, $[Ln(tfa)_3(\mu\text{-dppbo})]_n$, and $[Ln(hfa)_3(\mu\text{-dppbo})]_n$ (tfa$^-$: trifluoroacetylacetonate; hfa$^-$: hexafluoroacetylacetonate; L: [(diphenylphosphoryl)R](diphenyl)phosphine oxide, R = ethyl - dppeo - or butyl - dppbo); Ln = $Tb_{0.25}Eu_{0.75}$ or $Tb_{0.75}Tb_{0.25}$) coordination polymers were synthesized by dissolving the tris-β-diketonate aqua complex - $[Ln(tfa)_3(H_2O)_2]$ or $[Ln(hfa)_3(H_2O)_2]$ - precursor (0.2 mmol) in 20 mL of ethanol, followed by the addition of the dppeo or dppbo bridge ligand (0.1 mmol). The solution was kept undisturbed for 3 days at 300 K and after that, white crystals were obtained (yield of at about 60%). The Eu^{III}-to-Tb^{III} molar ratio was kept as 0.25 Tb^{III}: 0.75 Eu^{III} or 0.75 Tb^{III} : 0.25 Eu^{III} by changing the amount of tris-β-diketonate aqua complex added in the reaction. This combination results in eight samples, as represented in Table S1. All the measurements were undertaken by employing the crashed crystals.

Table S1. Representation of the chemical composition of the synthesized samples.

Sample	Tb-to-Eu ratio
**$[Tb_{0.25}Eu_{0.75}(tfa)_3(\mu\text{-dppeo})]_n$**<br>**$[Tb_{0.25}Eu_{0.75}(tfa)_3(\mu\text{-dppbo})]_n$**<br>**$[Tb_{0.25}Eu_{0.75}(hfa)_3(\mu\text{-dppeo})]_n$**<br>**$[Tb_{0.25}Eu_{0.75}(hfa)_3(\mu\text{-dppbo})]_n$**	0.25 Tb^{III}:0.75 Eu^{III}<br>$\{Tb_{0.25}Eu_{0.75}\}_n$
**$[Tb_{0.75}Eu_{0.25}(tfa)_3(\mu\text{-dppeo})]_n$**<br>**$[Tb_{0.75}Eu_{0.25}(tfa)_3(\mu\text{-dppbo})]_n$**<br>**$[Tb_{0.75}Eu_{0.25}(hfa)_3(\mu\text{-dppeo})]_n$**<br>**$[Tb_{0.75}Eu_{0.25}(hfa)_3(\mu\text{-dppbo})]_n$**	0.75 Tb^{III}:0.25 Eu^{III}<br>$\{Tb_{0.75}Eu_{0.25}\}_n$

SC-XRD (single-crystal X-ray diffraction). The data collection of $[Tb_{0.75}Eu_{0.25}(tfa)_3(\mu\text{-dppbo})]_n$ and $[Tb_{0.75}Eu_{0.25}(hfa)_3(\mu\text{-dppeo})]_n$ was performed employing the Rigaku XtaLAB Synergy-S diffractometer, equipped with a HyPix-6000HE detector and MoK_α (λ = 0.71073 Å at 100 K) or CuK_α (1.54184 Å at 100 K) radiation with a sealed X-ray source. The crystals were selected and mounted in cryoloop with mineral oil. The collection strategies and cell refinement were carried out using the CrysAlisPro. All crystal structures were solved by intrinsic phasing method employing SHELXT (version 2014/4) and refined using the least squares minimization on F^2 from SHELXL.[3] A gaussian absorption correction was performed.

PXRD (powder X-ray diffraction). PXRD within 5 - 40° was measured using a Shimadzu XRD 7000 (Cu K_α, λ= 1.5418 Å) diffractometer operating at 40 kV and 30 mA (300 K), with scan rate of 0.5° mim^{-1}.

FTIR (Fourier-transform infrared spectroscopy). FTIR of complexes in solid state was recorded in an Agilent Cary 600 Series FTIR Spectrophotometer (660) in the range of 4,000 to 400 cm^{-1} with a resolution of 2 cm^{-1} using an attenuated total reflectance (ATR) accessory.

PL (photoluminescence). PL was carried out in a Fluorolog-3 spectrofluorometer (Horiba FL3-22-iHR320) with double-gratings (1,200 grooves mm^{-1}, 330 nm blaze) in the excitation monochromator and double-gratings (1200 grooves mm^{-1}, 500 nm blaze) in the emission monochromator. An ozone-free xenon lamp of 450 W (Ushio) was used as a radiation source. A 150 W pulsed xenon lamp was used for time-resolved measurements by employing a time-correlated single-photon counting (TCSPC) system. A photomultiplier (Hamamatsu R928P) operating at 950 V was used to collect the spectra in the ultraviolet and visible spectral regions, which were corrected according to the optical system of the emission monochromator and the photomultiplier response. The emission and excitation spectra were carried out using the front face mode. The excitation and emission slits were kept in a position to lead to a spectral bandpass of 1 nm in all experiments. To control the temperature, a Linkam accessory (THMS600), with an optical fiber set (NA = 0.22 - Horiba-FL-3000/FM4-3000) was used.

Absolute emission quantum yield. Emission quantum yield (Φ_L^{Ln}) was measured in a Quanta -ϕ F-3029 integrating sphere coupled by optic fibers to the previously mentioned fluorimeter. For reference, the empty sphere coated with Spectralon® (reflectance > 95%) was used.

Luminescence thermometry. Emission spectra from 77 K to 430 K were measured in the previously mentioned Fluorolog 3 equipment. The excitation and emission slits were kept in a position to lead to a spectral bandpass of 1 nm in all experiments. To control the temperature, a Linkam accessory (THMS600), with an optical fiber set (NA = 0.22 - Horiba-FL-3000/FM4-3000) was used. The relative thermal sensitivity of the systems was calculated from equation S1, where *LIR* (luminescence intensity ratio, I_{Tb}/I_{Eu}) is the thermometric parameter and *T* is the temperature. The temperature uncertainty (δT) is calculated by equation S2 and equation S3, where $\delta I/I$ is the relative uncertainty in the integrated area.[4] δI was calculated from the signal-to-noise ratio for each normalized spectrum measured within the 555 – 595 nm spectral range, which was close to 0.02 for all of them. Thus, equation S3 can be reduced to $\delta I/I$ = 0.02 for all spectra considering the normalized spectra since I_{max} = 1.

$$S_r = \frac{1}{\Delta}\left|\frac{d(LIR)}{dT}\right| \quad (S1)$$

$$\delta T = \frac{1}{S_r}\frac{\delta(LIR)}{LIR} \quad (S2)$$

$$\frac{\delta(LIR)}{LIR} = \sqrt{\left(\frac{\delta I_1}{I_1}\right)^2 + \left(\frac{\delta I_2}{I_2}\right)^2} = \sqrt{2}\frac{\delta I}{I} \quad (S3)$$

Supplementary note S2 – Structural data

Table S2. Ln^{III} ··· Ln^{III} inter/intramolecular distances in the coordination polymers. The values were reproduced from the crystal structures previously reported by us (Ln = Er^{III}, Yb^{III}).[2]

Composition	Intramolecular Ln ··· Ln distance (Å)	Intermolecular Ln ··· Ln distance (Å)
**$[Ln(tfa)_3(\mu\text{-dppeo})]_n$**	8.2958	11.6276
**$[Ln(tfa)_3(\mu\text{-dppbo})]_n$**	9.2853	8.1045
**$[Ln(hfa)_3(\mu\text{-dppeo})]_n$**	8.4443	12.4334
**$[Ln(hfa)_3(\mu\text{-dppbo})]_n$**	10.7704	11.7562

Table S3. Crystal data and refinement parameters for $[Tb_{0.75}Eu_{0.25}(tfa)_3(\mu\text{-dppbo})]_n$ and $[Tb_{0.75}Eu_{0.25}(hfa)_3(\mu\text{-dppeo})]_n$.

	$[Tb_{0.75}Eu_{0.25}(tfa)_3(\mu\text{-dppbo})]_n$	$[Tb_{0.75}Eu_{0.25}(hfa)_3(\mu\text{-dppeo})]_n$
**CCDC number**	2532370	2532371
**Formula**	$C_{43}H_{40}Eu_{0.25}F_9O_8P_2Tb_{0.75}$	$C_{41}H_{27}Eu_{0.25}F_{18}O_8P_2Tb_{0.75}$
**$D_{calc.}$(g cm^{-3})**	1.585	1.805
**m (mm^{-1})**	1.642	10.825
**Formula Weight**	1073.13	1207.00
**Size (mm^3)**	0.17×0.04×0.04	0.17×0.02×0.01
**T (K)**	100(2)	100(2)
**Crystal System**	Triclinic	triclinic
**Space Group**	$P\bar{1}$	$P\bar{1}$
**a (Å)**	11.5465(2)	13.0658(4)
**b (Å)**	13.5242(2)	13.3250(4)
**c (Å)**	15.2416(2)	14.2203(4)
**α (°)**	81.9250(10)	105.564(2)
**β (°)**	72.6420(10)	101.967(3)
**γ (°)**	87.9370(10)	103.478(2)
**V (Å^3)**	2249.13(6)	2220.98(12)
**Z**	2	2
**Wavelength (Å)**	0.71073 (MoK$_\alpha$)	1.54184 (CuK$_\alpha$)
**θ_{min} - θ_{max}**	2.372 -25.682	5.345 - 70.075
**Measured Refl.**	37727	28115
**Independent Refl.**	8529	8359
**Reflections with I > 2(I)**	7936	7743
**R_{int}**	0.0341	0.0601
**Parameters**	581	641
**Restraints**	3	3
**Largest Peak / deepest hole**	0.744, -0.600	1.31, -1.63
**GooF**	1.056	1.049
**wR_2, R_1 (all data)**	0.0515, 0.0247	0.1071, 0.0471
**wR_2, R_1**	0.0515, 0.0219	0.1050, 0.0428

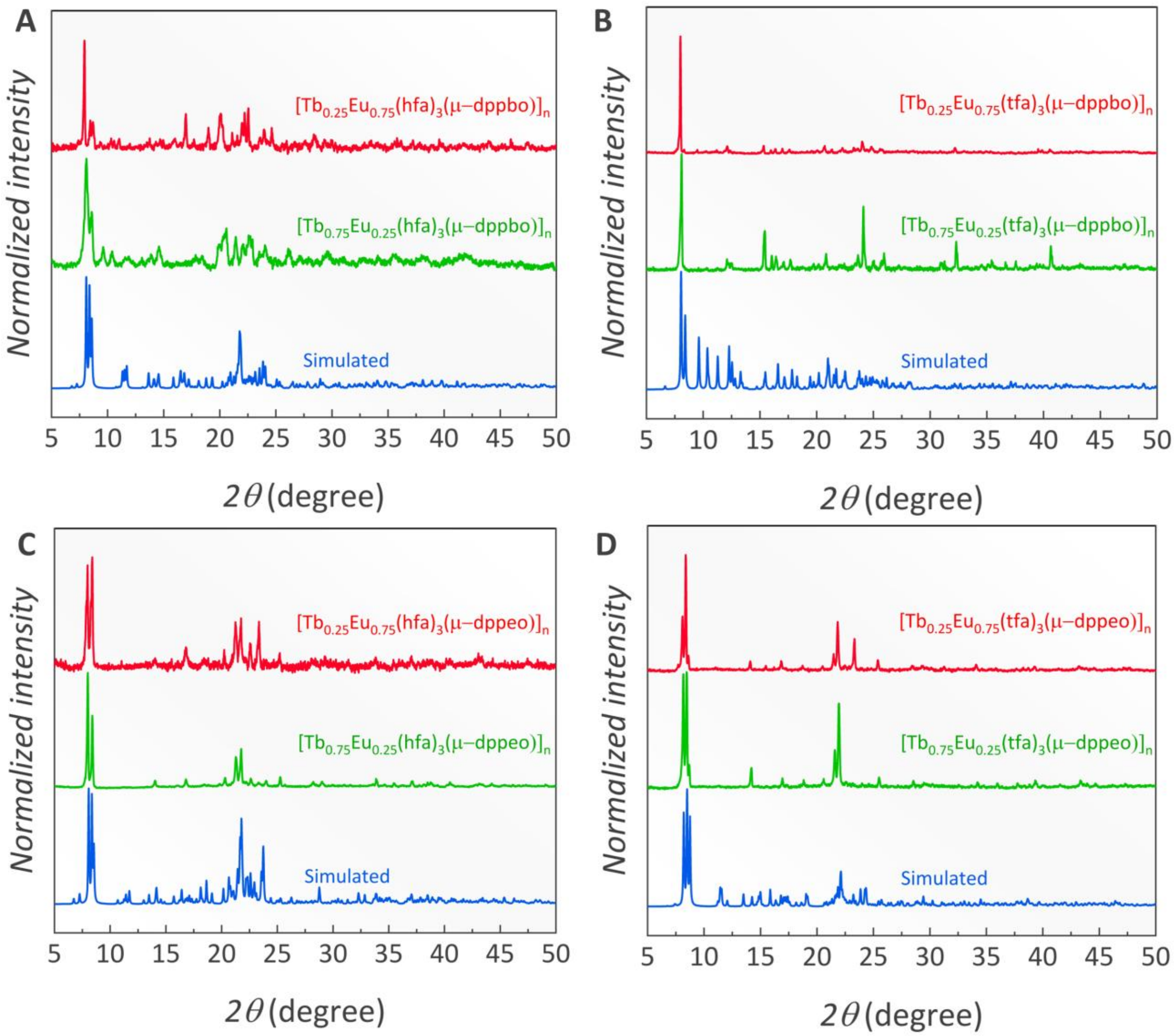

Figure S1. PXRD of (A) $[Ln(hfa)_3(\mu\text{-dppbo})]_n$ (B) $[Ln(tfa)_3(\mu\text{-dppbo})]_n$, (C) $[Ln(hfa)_3(\mu\text{-dppeo})]_n$, and (D) $[Ln(tfa)_3(\mu\text{-dppeo})]_n$ series compared to the simulated pattern from the SC-XRD measurement. The PXRD data were collected for the crashed crystals at 300 K. It should be noted that, for the $[Ln(hfa)_3(\mu\text{-dppbo})]_n$ compositions, two peaks near 9.6° and 10.4° are absent in the simulated PXRD patterns generated from the SC-XRD data, which is likely due to the presence of unreacted bridge ligand. This ligand is optically inactive and should not interfere in the photoluminescence measurements. As observed from time-resolved spectroscopy (Figures S6 and S7), all coordination polymers present a monoexponencial decay behaviour, which confirms the presence of only one luminescent-active center.

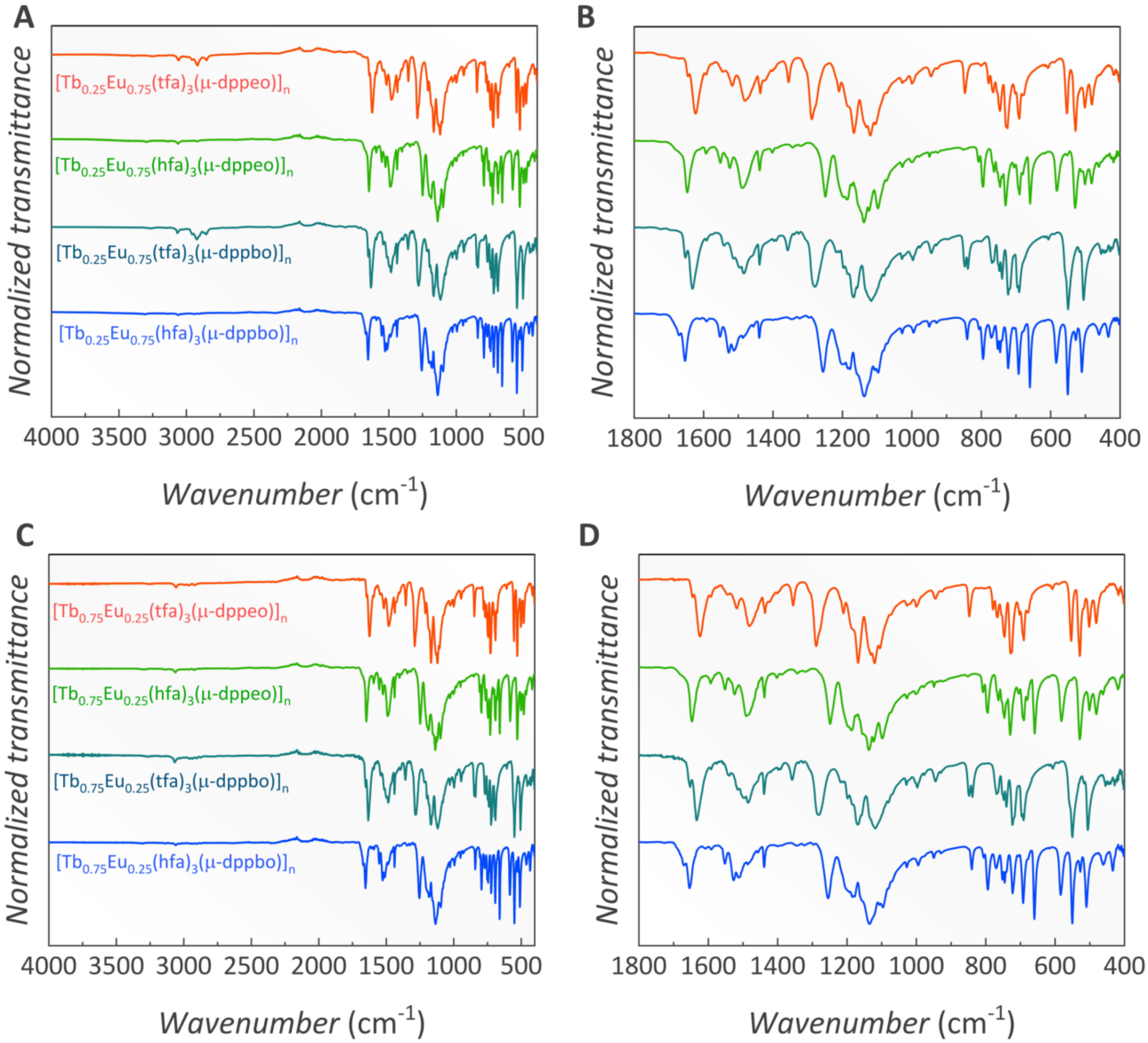

Figure S2. FTIR of (A) $\{Tb_{0.25}Eu_{0.75}\}_n$ and (B) $\{Tb_{0.75}Eu_{0.25}\}_n$. Figures (C) and (D) show the magnification within the 1800 – 400 cm^{-1} range. The spectra were collected for the crashed crystals at 300 K.

Supplementary note S3 – Steady-state photoluminescence

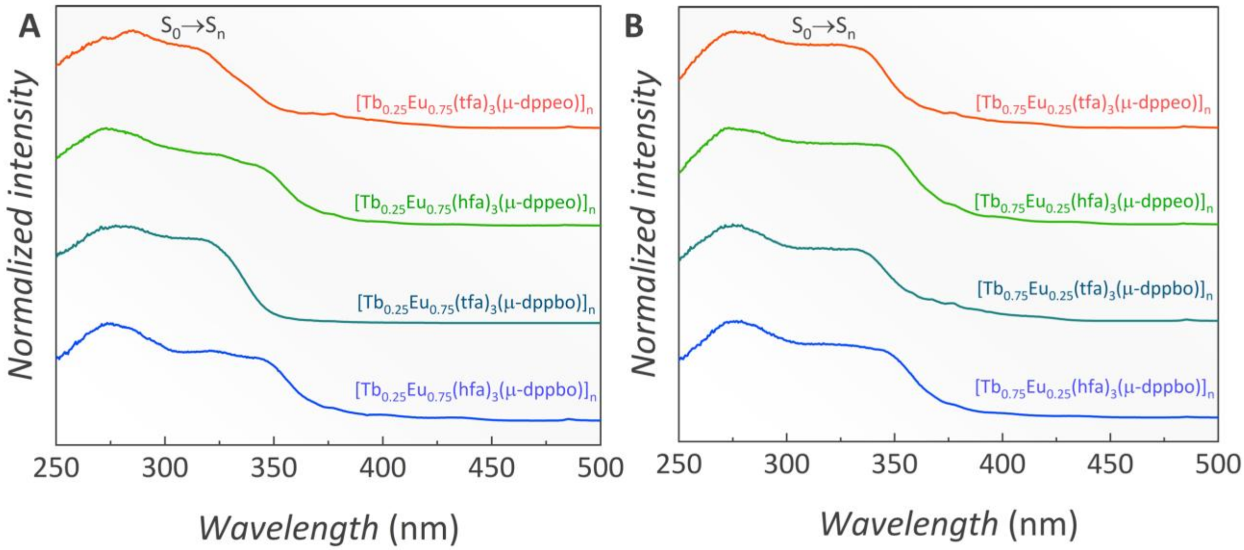

Figure S3. Excitation spectra (300 K) monitoring the Tb^{III} emission at 542 nm for (A) $\{Tb_{0.25}Eu_{0.75}\}_n$ and (B) $\{Tb_{0.75}Eu_{0.25}\}_n$.

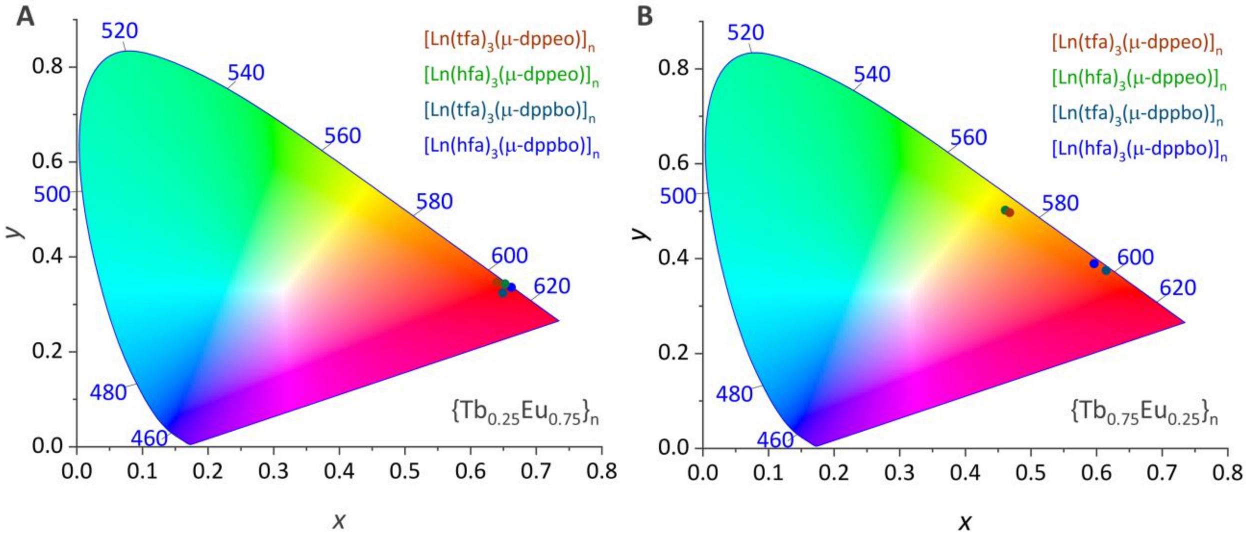

Figure S4. 1931 *Commission internationale de l'éclairage* (CIE) color coordinate diagrams illustrating the emission color coordinates of the (A) $\{Tb_{0.25}Eu_{0.75}\}_n$ and (B) $\{Tb_{0.75}Eu_{0.25}\}_n$ coordination polymers monitored at 340 nm (300 K).

Table S4. Color coordinates calculated from the CIE 1931 space for the coordination polymers monitored at 340 nm (300 K).

	(x,y)
**$[Tb_{0.25}Eu_{0.75}(hfa)_3(\mu\text{-dppbo})]_n$**	(0.66183, 0.33611)
**$[Tb_{0.75}Eu_{0.25}(hfa)_3(\mu\text{-dppbo})]_n$**	(0.46084, 0.50211)
**$[Tb_{0.25}Eu_{0.75}(tfa)_3(\mu\text{-dppbo})]_n$**	(0.64887, 0.32414)
**$[Tb_{0.75}Eu_{0.25}(tfa)_3(\mu\text{-dppbo})]_n$**	(0.61448, 0.37516)
**$[Tb_{0.25}Eu_{0.75}(hfa)_3(\mu\text{-dppeo})]_n$**	(0.65235, 0.34362)
**$[Tb_{0.75}Eu_{0.25}(hfa)_3(\mu\text{-dppeo})]_n$**	(0.59607, 0.3895)
**$[Tb_{0.25}Eu_{0.75}(tfa)_3(\mu\text{-dppeo})]_n$**	(0.63992, 0.3469)
**$[Tb_{0.75}Eu_{0.25}(tfa)_3(\mu\text{-dppeo})]_n$**	(0.4675, 0.49713)

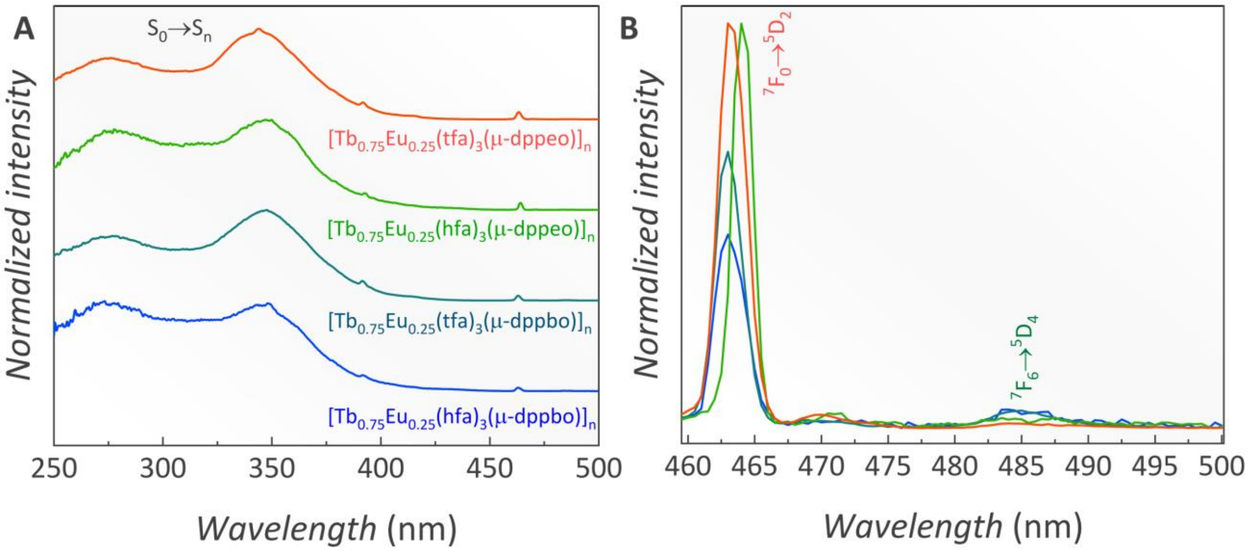

Figure S5. (A) Excitation spectra (300 K) monitoring the Eu^{III} emission at 700 nm ($^5D_0 \rightarrow {}^7F_4$ transition) for the $\{Tb_{0.75}Eu_{0.25}\}_n$ series. (B) Magnification within the 460 – 500 nm spectral range, highlighting the Tb^{III} $^7F_6 \rightarrow {}^5D_4$ excitation transition.

Supplementary note S4 – Time-resolved photoluminescence

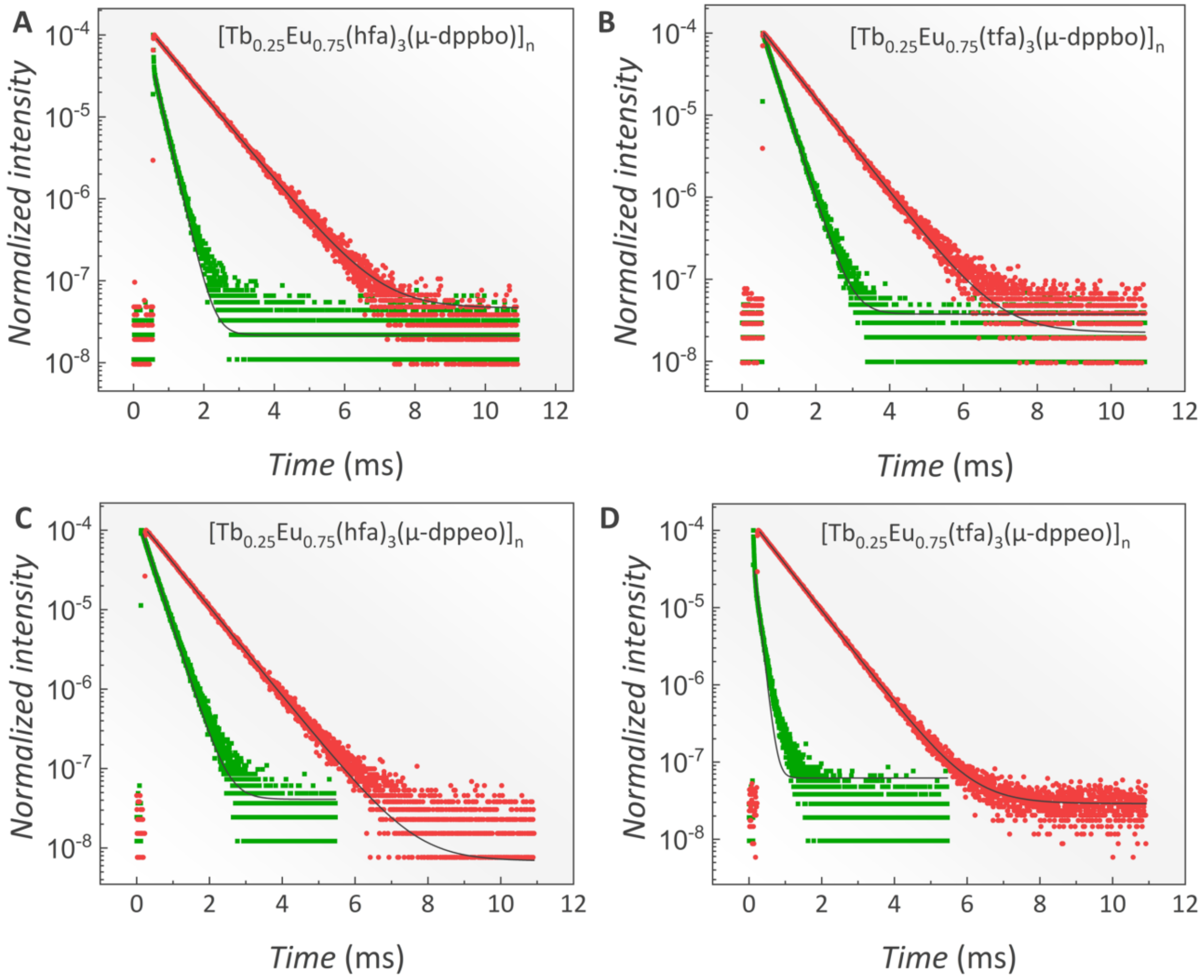

Figure S6. Emission decay curves (300 K) for the (A) $[Tb_{0.25}Eu_{0.75}(hfa)_3(\mu\text{-dppbo})]_n$, (B) $[Tb_{0.25}Eu_{0.75}(tfa)_3(\mu\text{-dppbo})]_n$, (C) $[Tb_{0.25}Eu_{0.75}(hfa)_3(\mu\text{-dppeo})]_n$, and (D) $[Tb_{0.25}Eu_{0.75}(tfa)_3(\mu\text{-dppeo})]_n$ series monitoring the excitation at 340 nm and the Eu^{III} emission at 612 nm (red) or Tb^{III} emission at 542 nm (green). The fitting to a monoexponential function is also represented in the black line ($R^2 > 0.95$).

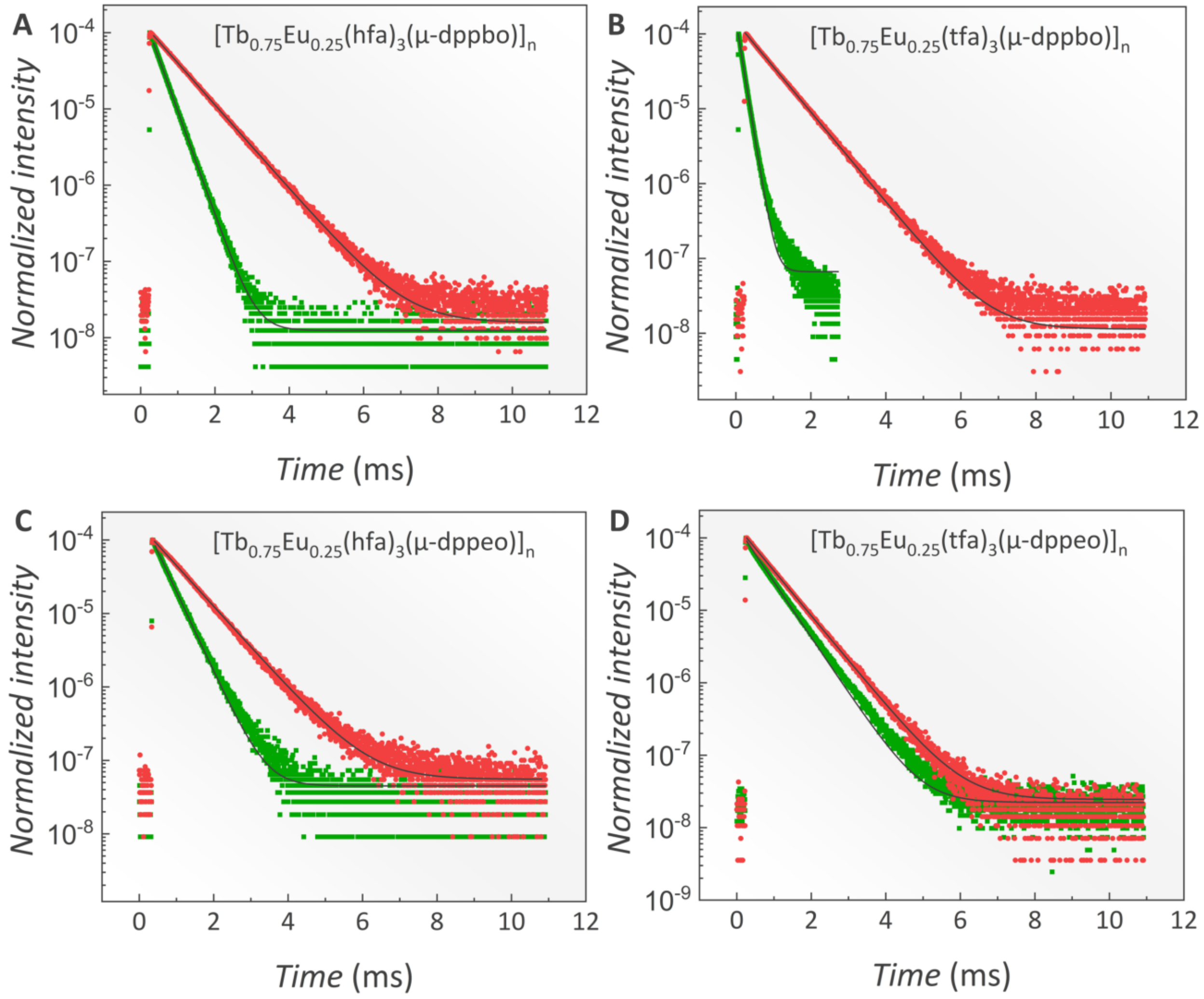

Figure S7. Emission decay curves (300 K) for the (A) $[Tb_{0.75}Eu_{0.25}(hfa)_3(\mu\text{-dppbo})]_n$, (B) $[Tb_{0.75}Eu_{0.25}(tfa)_3(\mu\text{-dppbo})]_n$, (C) $[Tb_{0.75}Eu_{0.25}(hfa)_3(\mu\text{-dppeo})]_n$, and (D) $[Tb_{0.75}Eu_{0.25}(tfa)_3(\mu\text{-dppeo})]_n$ series monitoring the excitation at 340 nm and the Eu^{III} emission at 612 nm (red) or Tb^{III} emission at 542 nm (green). The fitting to a monoexponential function is also represented in the black line ($R^2 > 0.95$).

Table S5. Eu^{III} $^5D_0$ (τ_{Eu}) and Tb^{III} $^5D_4$ (τ_{Tb}) level lifetimes and overall emission quantum yield (Φ_{Ln}^{L}) measured at 300 K.

	τ_{Eu} (ms)	τ_{Tb} (ms)	Φ_{Ln}^{L} (%)
**$[Tb_{0.25}Eu_{0.75}(hfa)_3(\mu\text{-dppbo})]_n$**	0.8463±0.0002	0.2451±0.0002	57.1±6
**$[Tb_{0.75}Eu_{0.25}(hfa)_3(\mu\text{-dppbo})]_n$**	0.8155±0.0003	0.2114±0.0001	29.5±3
**$[Tb_{0.25}Eu_{0.75}(tfa)_3(\mu\text{-dppbo})]_n$**	0.8003±0.0004	0.5400±0.0012	42.8±4
**$[Tb_{0.75}Eu_{0.25}(tfa)_3(\mu\text{-dppbo})]_n$**	0.7202±0.0005	0.1396±0.0006	27.6±3
**$[Tb_{0.25}Eu_{0.75}(hfa)_3(\mu\text{-dppeo})]_n$**	0.7612±0.0005	0.3092±0.0004	51.5±5
**$[Tb_{0.75}Eu_{0.25}(hfa)_3(\mu\text{-dppeo})]_n$**	0.7778±0.0005	0.3973±0.0004	32.5±3
**$[Tb_{0.25}Eu_{0.75}(tfa)_3(\mu\text{-dppeo})]_n$**	0.7244±0.0002	0.1047±0.0006	36.5±4
**$[Tb_{0.75}Eu_{0.25}(tfa)_3(\mu\text{-dppeo})]_n$**	0.7005±0.0004	0.5657±0.0008	48.2±4

Supplementary note S5 – Temperature-dependent photoluminescence data

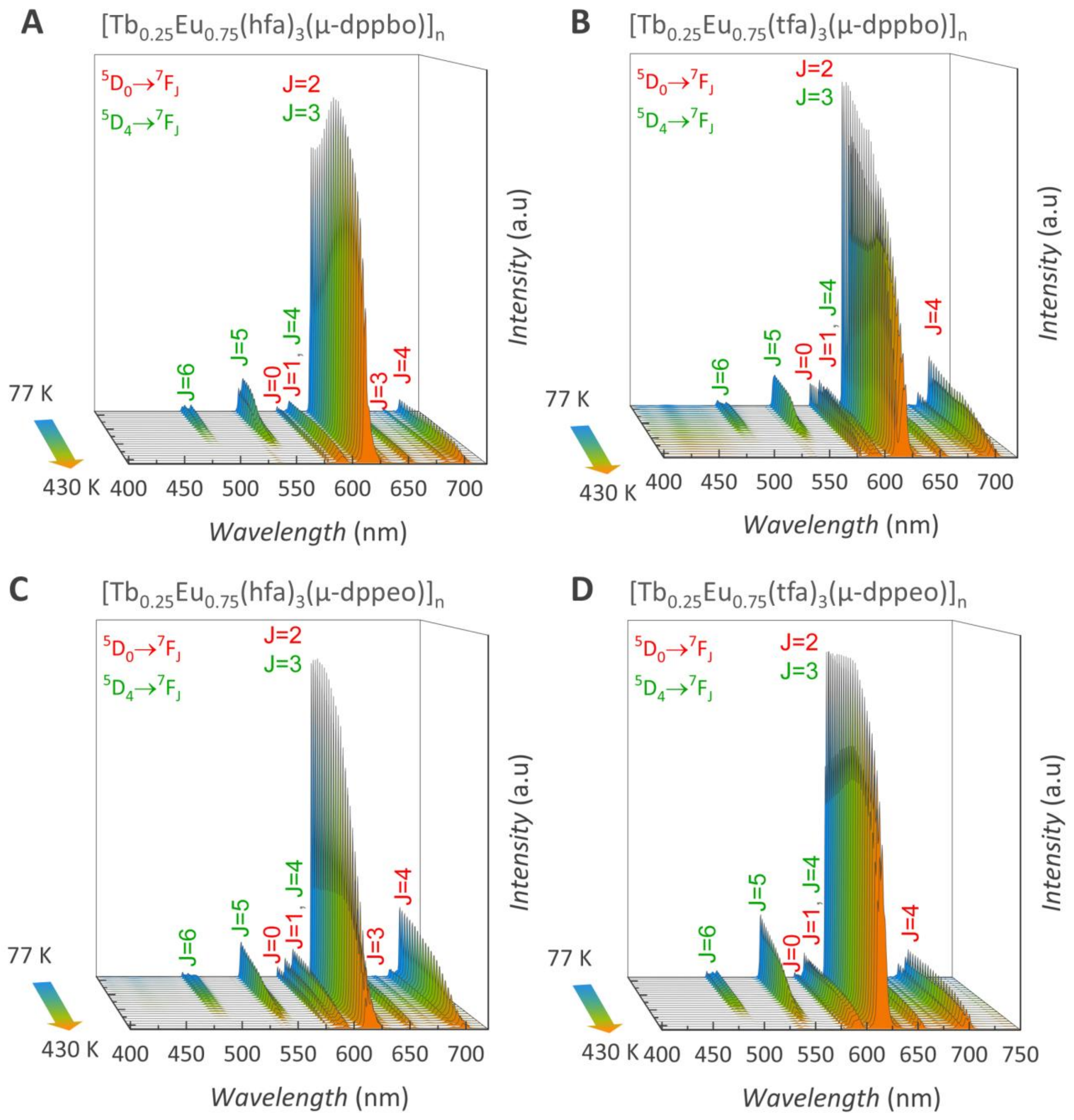

Figure S8. Temperature-dependent (77 – 430 K) emission spectra (λ_{exc} = 340 nm) for (A) $[Tb_{0.25}Eu_{0.75}(hfa)_3(\mu\text{-dppbo})]_n$, (B) $[Tb_{0.25}Eu_{0.75}(tfa)_3(\mu\text{-dppbo})]_n$, (C) $[Tb_{0.25}Eu_{0.75}(hfa)_3(\mu\text{-dppeo})]_n$, and (D) $[Tb_{0.25}Eu_{0.75}(tfa)_3(\mu\text{-dppeo})]_n$. The emission spectra for the compositions $\{Tb_{0.75}Eu_{0.25}\}_n$ are shown in Figure 3. Eu^{III} and Tb^{III} transitions are represented in red and green, respectively.

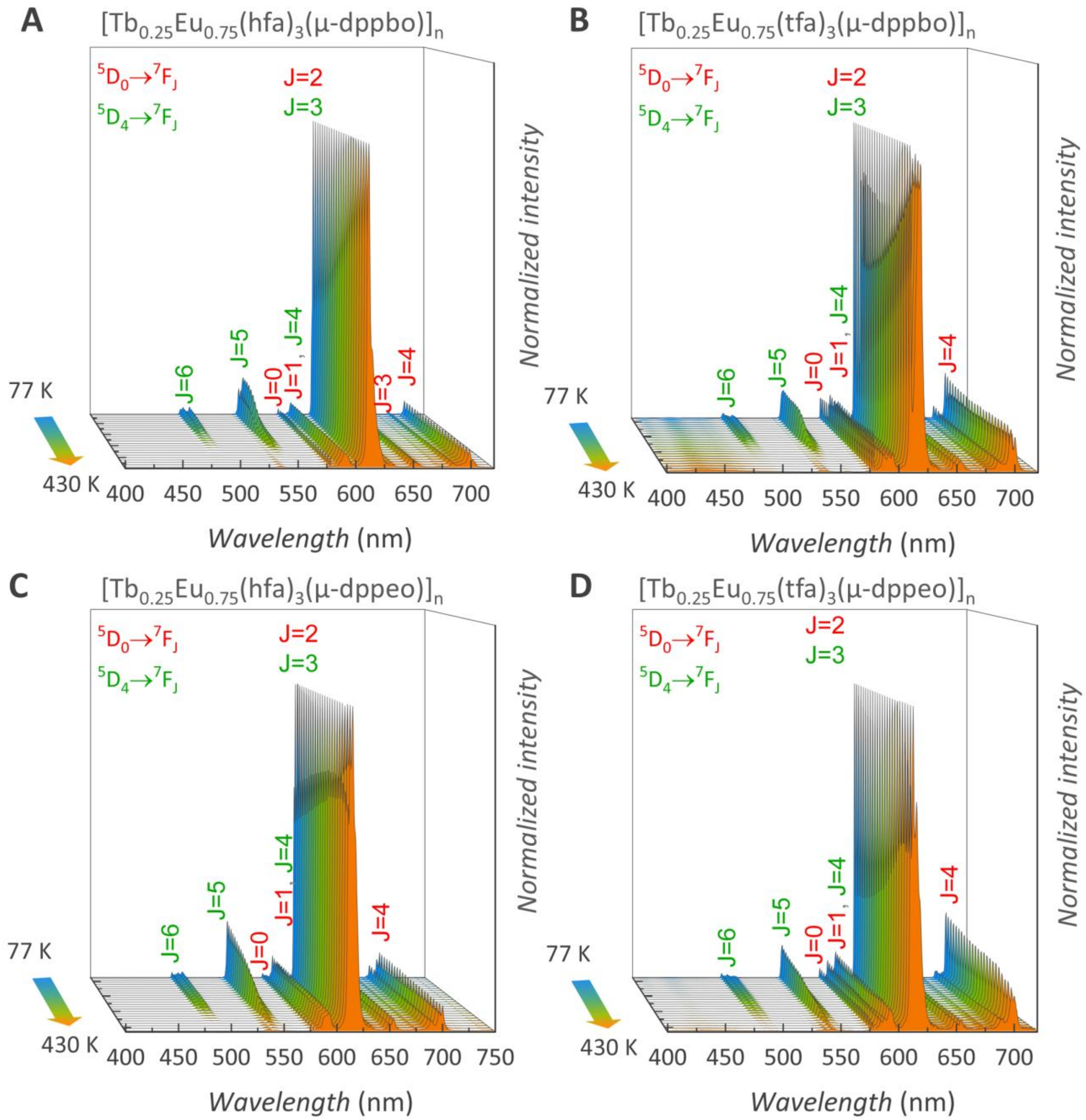

Figure S9. Normalized temperature-dependent (77 – 430 K) emission spectra (λ_{exc} = 340 nm) for (A) $[Tb_{0.25}Eu_{0.75}(hfa)_3(\mu\text{-dppbo})]_n$, (B) $[Tb_{0.25}Eu_{0.75}(tfa)_3(\mu\text{-dppbo})]_n$, (C) $[Tb_{0.25}Eu_{0.75}(hfa)_3(\mu\text{-dppeo})]_n$, and (D) $[Tb_{0.25}Eu_{0.75}(tfa)_3(\mu\text{-dppeo})]_n$. Eu^{III} and Tb^{III} transitions are represented in red and green, respectively.

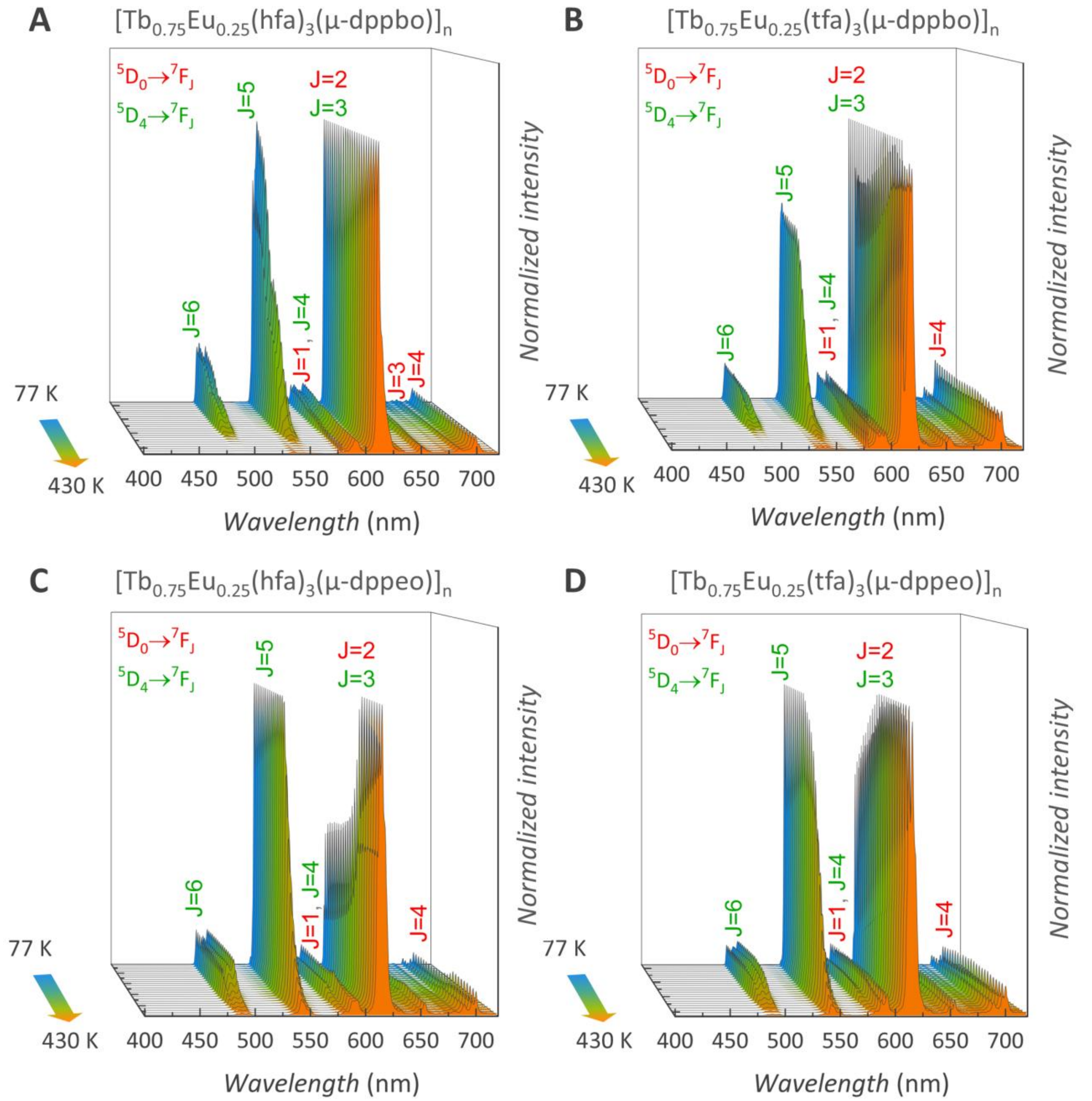

Figure S10. Normalized temperature-dependent (77 – 430 K) emission spectra (λ_{exc} = 340 nm) for (A) $[Tb_{0.75}Eu_{0.25}(hfa)_3(\mu\text{-dppbo})]_n$, (B) $[Tb_{0.75}Eu_{0.25}(tfa)_3(\mu\text{-dppbo})]_n$, (C) $[Tb_{0.75}Eu_{0.25}(hfa)_3(\mu\text{-dppeo})]_n$, and (D) $[Tb_{0.75}Eu_{0.25}(tfa)_3(\mu\text{-dppeo})]_n$. Eu^{III} and Tb^{III} transitions are represented in red and green, respectively.

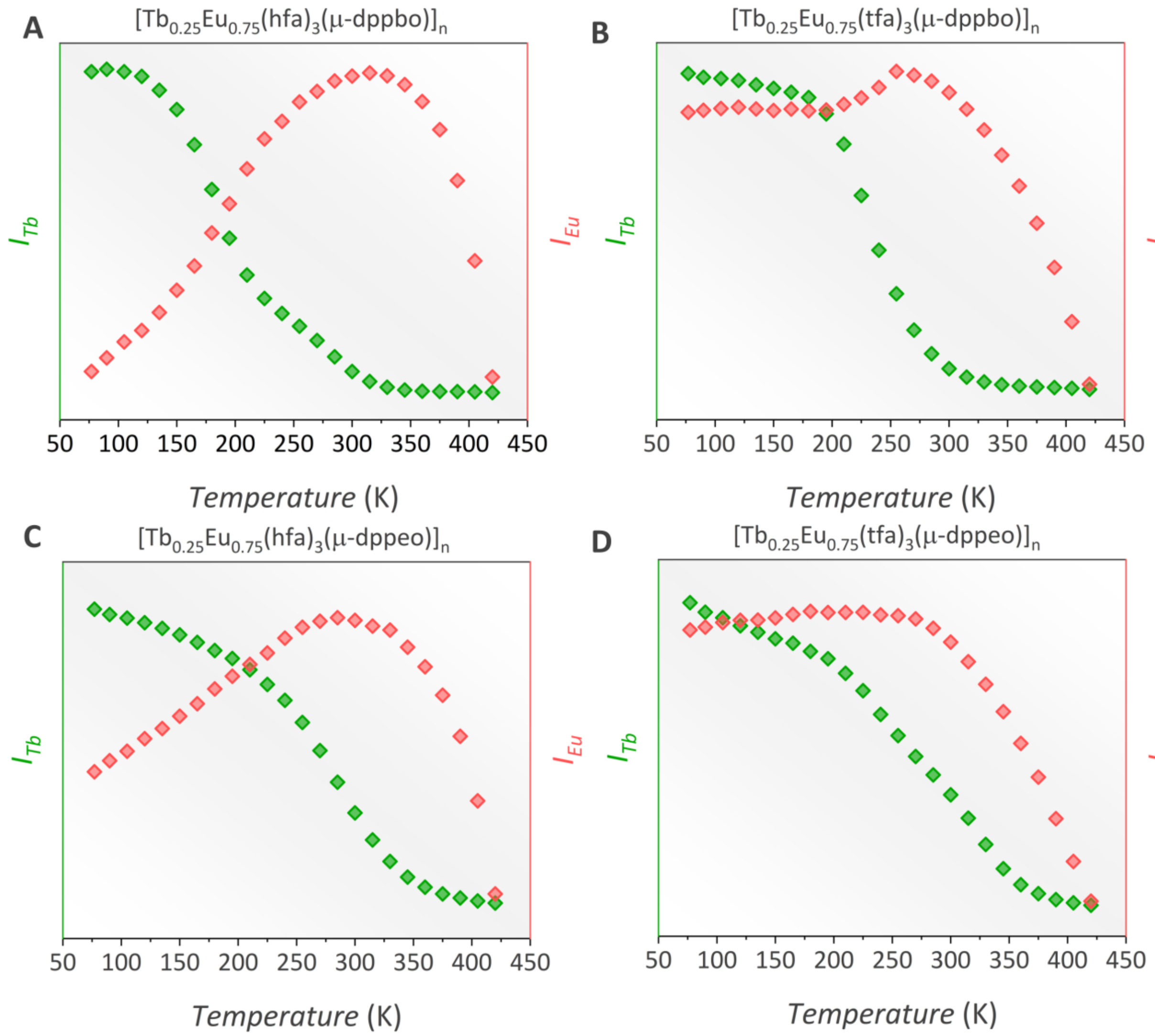

Figure S11. Temperature dependence of the Tb^{III} $^5D_4 \rightarrow {}^7F_5$ (I_{Tb}, 535–565 nm) and Eu^{III} $^5D_0 \rightarrow {}^7F_2$ (I_{Eu}, 605–635 nm, with some contribution of overlapped Tb^{III} $^5D_4 \rightarrow {}^7F_3$ transition) band intensities for (A) $[Tb_{0.25}Eu_{0.75}(hfa)_3(\mu\text{-dppbo})]_n$, (B) $[Tb_{0.25}Eu_{0.75}(tfa)_3(\mu\text{-dppbo})]_n$, (C) $[Tb_{0.25}Eu_{0.75}(hfa)_3(\mu\text{-dppeo})]_n$, and (D) $[Tb_{0.25}Eu_{0.75}(tfa)_3(\mu\text{-dppeo})]_n$.

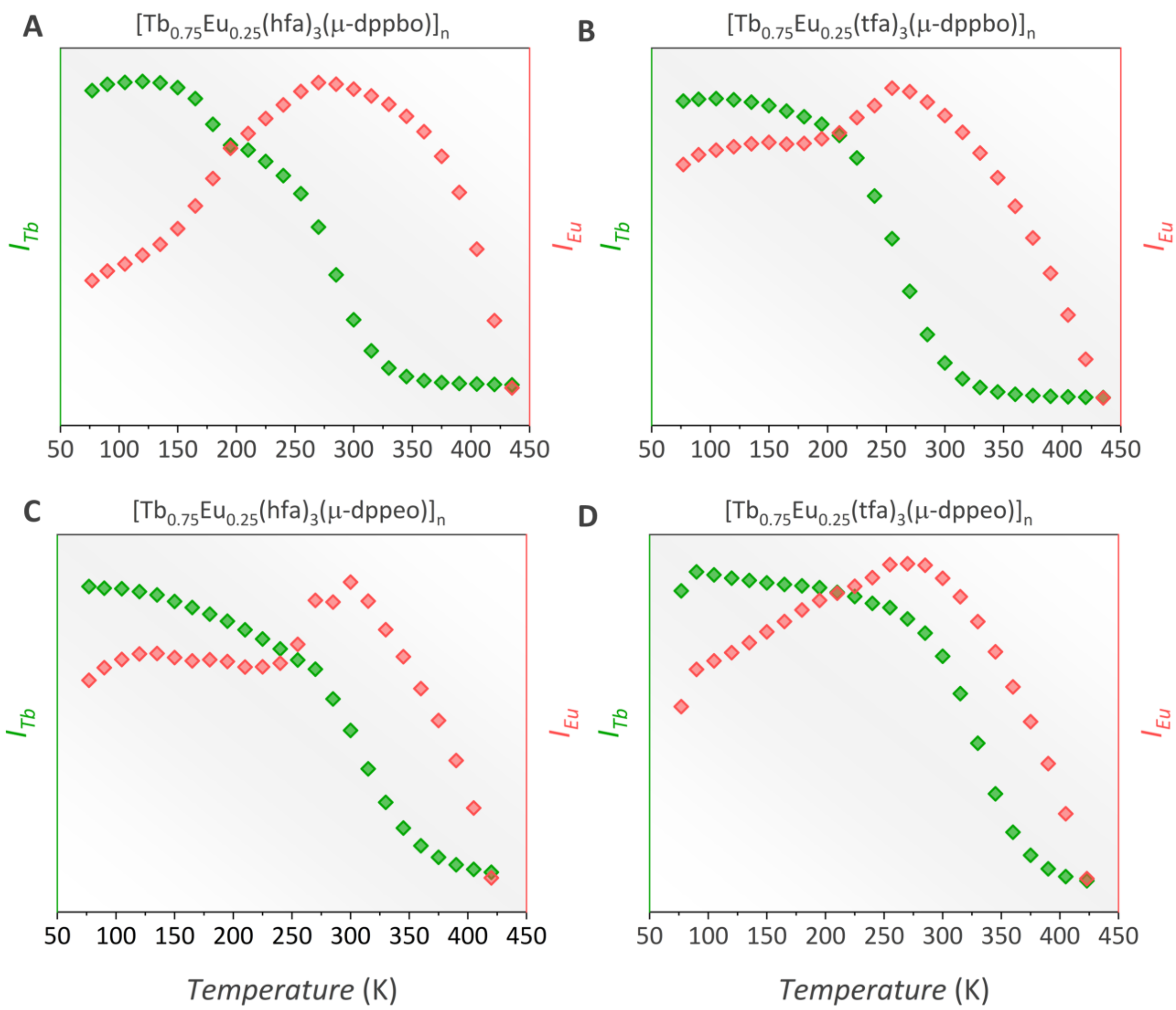

Figure S12. Temperature dependence of the Tb^{III} $^5D_4 \rightarrow {}^7F_5$ (I_{Tb}, 535−565 nm) and Eu^{III} $^5D_0 \rightarrow {}^7F_2$ (I_{Eu}, 605−635 nm, with some contribution of overlapped Tb^{III} $^5D_4 \rightarrow {}^7F_3$ transition) band intensities for (A) [$Tb_{0.75}Eu_{0.25}$(hfa)$_3$(μ-dppbo)]$_n$, (B) [$Tb_{0.75}Eu_{0.25}$(tfa)$_3$(μ-dppbo)]$_n$, (C) [$Tb_{0.75}Eu_{0.25}$(hfa)$_3$(μ-dppeo)]$_n$, and (D) [$Tb_{0.75}Eu_{0.25}$(tfa)$_3$(μ-dppeo)]$_n$.

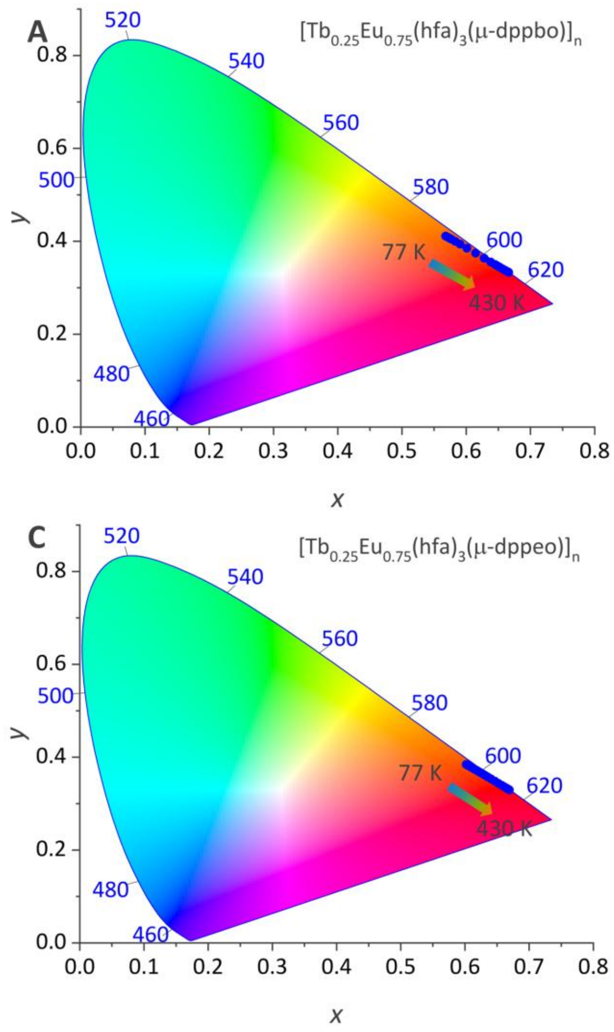

Figure S13. 1931 *Commission internationale de l'éclairage* (CIE) color coordinate diagrams illustrating the emission color coordinates depending on the temperature for (A) $[Tb_{0.25}Eu_{0.75}(hfa)_3(\mu\text{-dppbo})]_n$, (B) $[Tb_{0.25}Eu_{0.75}(tfa)_3(\mu\text{-dppbo})]_n$, (C) $[Tb_{0.25}Eu_{0.75}(hfa)_3(\mu\text{-dppeo})]_n$, and (D) $[Tb_{0.25}Eu_{0.75}(tfa)_3(\mu\text{-dppeo})]_n$. The emission spectra were collected upon 340 nm excitation.

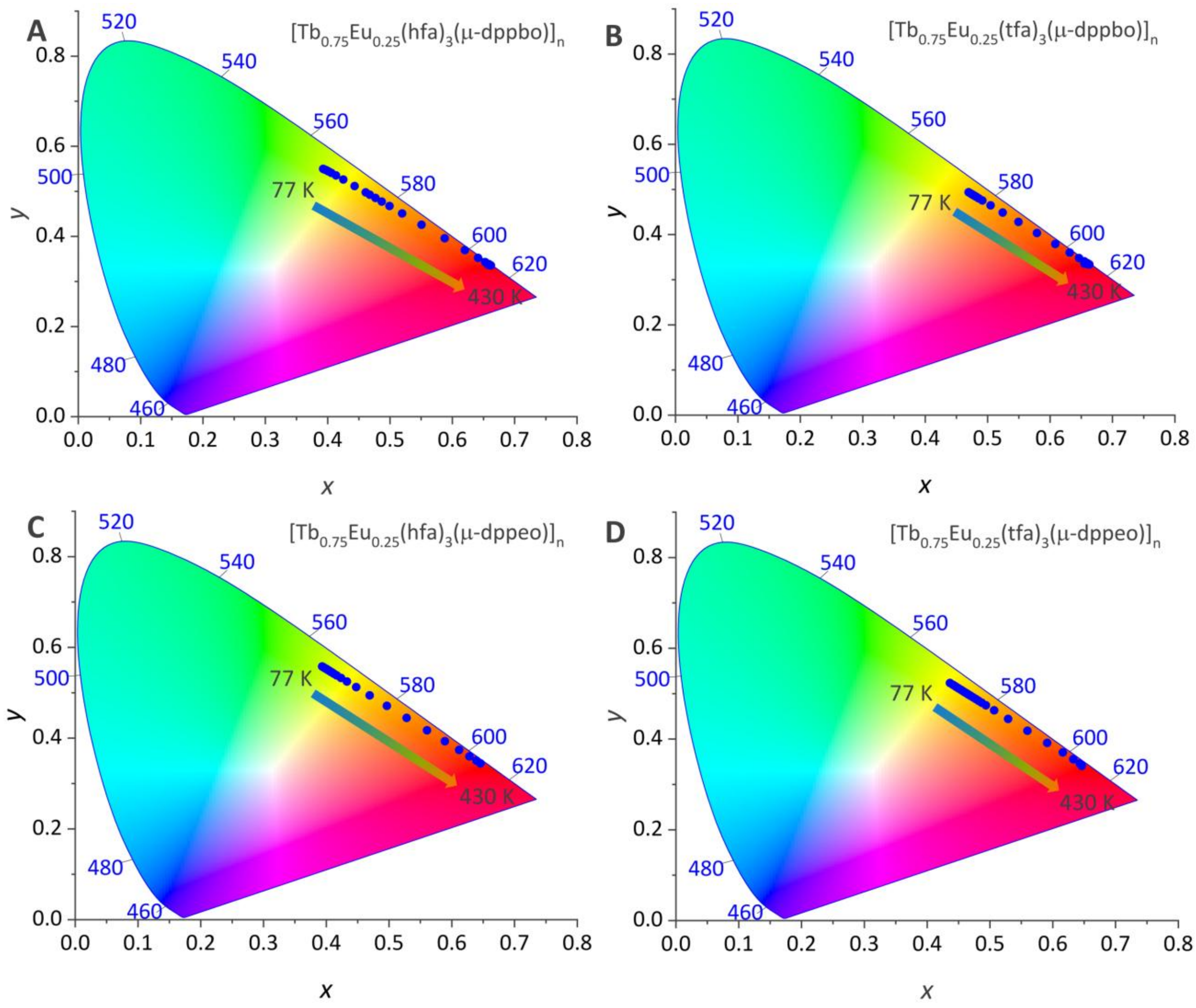

Figure S14. 1931 *Commission internationale de l'éclairage* (CIE) color coordinate diagrams illustrating the emission color coordinates depending on the temperature for (A) $[Tb_{0.75}Eu_{0.25}(hfa)_3(\mu\text{-dppbo})]_n$, (B) $[Tb_{0.75}Eu_{0.25}(tfa)_3(\mu\text{-dppbo})]_n$, (C) $[Tb_{0.75}Eu_{0.25}(hfa)_3(\mu\text{-dppeo})]_n$, and (D) $[Tb_{0.75}Eu_{0.25}(tfa)_3(\mu\text{-dppeo})]_n$. The emission spectra were collected upon 340 nm excitation.

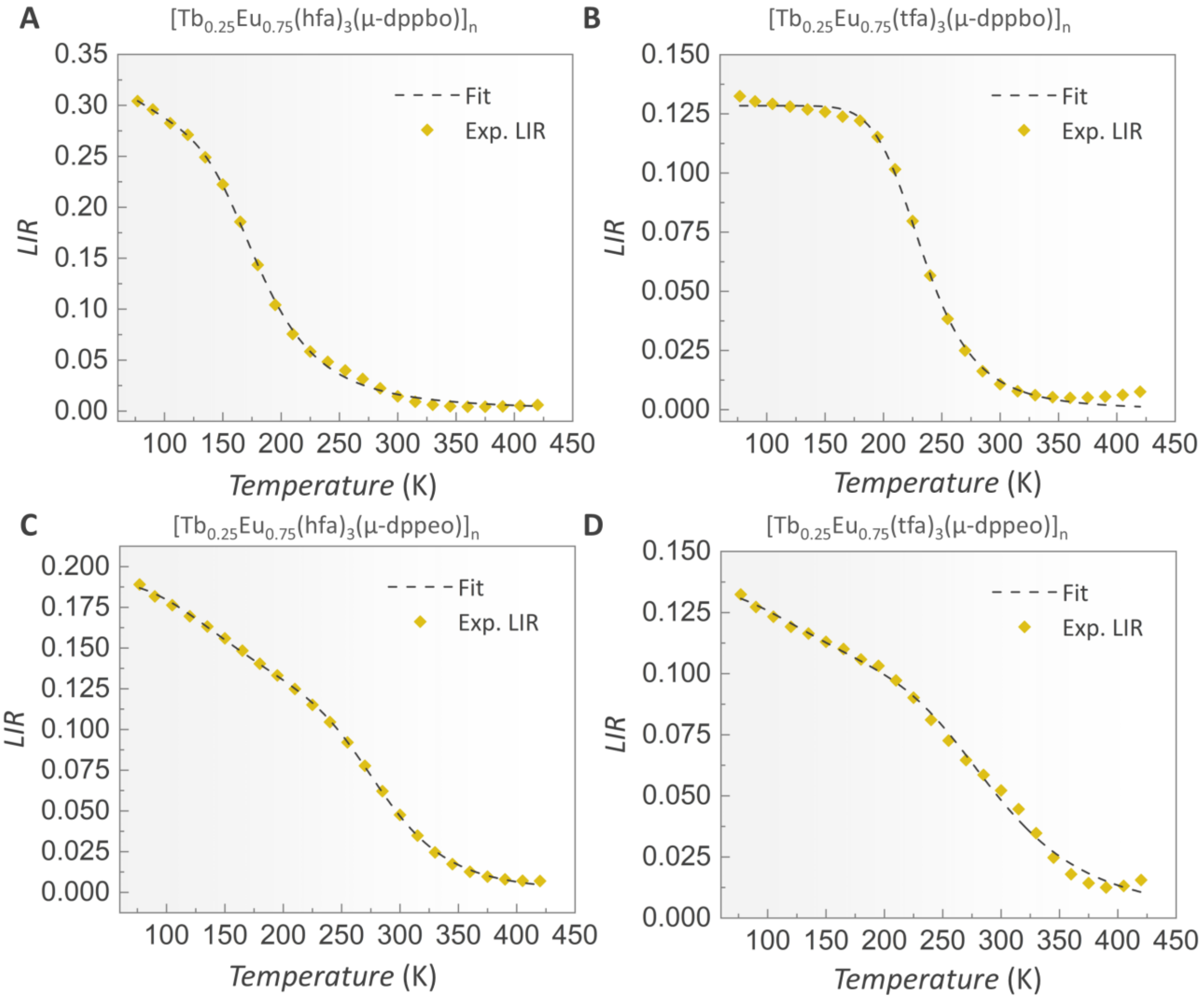

Figure S15. Dependence of the thermometric parameter (LIR = I_{Tb}/I_{Eu}) on the temperature for (A) $[Tb_{0.25}Eu_{0.75}(hfa)_3(\mu\text{-dppbo})]_n$, (B) $[Tb_{0.25}Eu_{0.75}(tfa)_3(\mu\text{-dppbo})]_n$, (C) $[Tb_{0.25}Eu_{0.75}(hfa)_3(\mu\text{-dppeo})]_n$, and (D) $[Tb_{0.25}Eu_{0.75}(tfa)_3(\mu\text{-dppeo})]_n$. The fitting of delta on the temperature following equation 1 is also represented in the dashed black line.

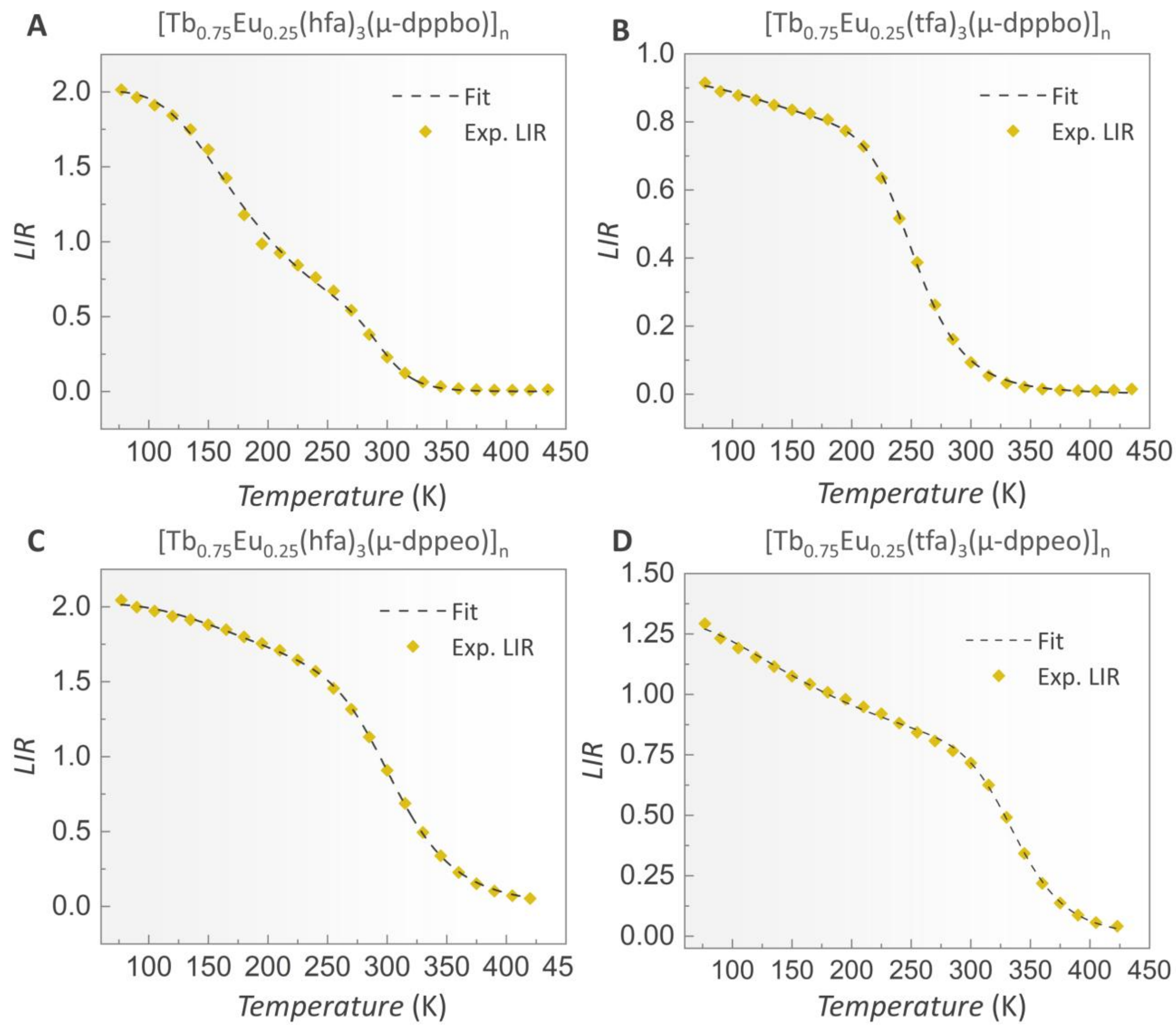

Figure S16. Dependence of the thermometric parameter (LIR = I_{Tb}/I_{Eu}) on the temperature for (A) $[Tb_{0.75}Eu_{0.25}(hfa)_3(\mu\text{-dppbo})]_n$, (B) $[Tb_{0.75}Eu_{0.25}(tfa)_3(\mu\text{-dppbo})]_n$, (C) $[Tb_{0.75}Eu_{0.25}(hfa)_3(\mu\text{-dppeo})]_n$, and (D) $[Tb_{0.75}Eu_{0.25}(tfa)_3(\mu\text{-dppeo})]_n$. The fitting of delta on the temperature following equation 1 is also represented in the dashed black line.

Table S6. Fitting parameters obtained from the thermometric parameter dependence on the temperature using equation 1.

	Δ_0	α_1	ΔE_1 (cm^{-1})	α_2	ΔE_2 (cm^{-1})
**[$Tb_{0.25}Eu_{0.75}(hfa)_3(\mu$-dppbo)]$_n$**	0.325	1.02	144	1.60 10^3	924
**[$Tb_{0.75}Eu_{0.25}(hfa)_3(\mu$-dppbo)]$_n$**	2.00	35.6	500	2.69 10^{10}	4670
**[$Tb_{0.25}Eu_{0.75}(tfa)_3(\mu$-dppbo)]$_n$**	0.128	-	-	3.48 10^4	1707
**[$Tb_{0.75}Eu_{0.25}(tfa)_3(\mu$-dppbo)]$_n$**	0.917	0.835	219	3.75 10^5	2226
**[$Tb_{0.25}Eu_{0.75}(hfa)_3(\mu$-dppeo)]$_n$**	0.190	3.42	281	5.23 10^4	2087
**[$Tb_{0.75}Eu_{0.25}(hfa)_3(\mu$-dppeo)]$_n$**	2.02	1.98	341	3.14 10^5	2652
**[$Tb_{0.25}Eu_{0.75}(tfa)_3(\mu$-dppeo)]$_n$**	0.134	1.53	216	2.481 10^3	1572
**[$Tb_{0.75}Eu_{0.25}(tfa)_3(\mu$-dppeo)]$_n$**	1.30	1.92	229	1.95 10^7	3809

Supplementary note S6 – Computational calculations

Computational methodology. In this work, only the positions of the hydrogen atoms were optimized through density functional theory (DFT) starting from the single-crystal structures. The optimization was performed with the PBE0 functional[5] along with the D4 dispersion correction[6]. Aiming at an accurate optimization, we employed the Def2-TZVP basis set[7] with no auxiliary resolution of identity (RI) approximation. After this step, a time-dependent DFT (TD-DFT) calculation with the ωB97x-D4 functional[8] was employed to obtain the energies of the singlet and triplet excited states, allowing to estimate the donor-acceptor distances involved in the ligand-to-Ln^{III} energy transfer. The TD-DFT calculation was carried out with the same Def2-TZVP basis set for all atoms except Eu^{III} and Tb^{III}, as they were described by the adapted Stuttgart-Cologne MWB52 and MWB54 effective core potentials (ECP, pseudopotentials)[9,10], respectively. We highlight that these ECPs are widely used for such calculations, as they allow us to consider the systems' multiplicity as singlet due to the buried nature of the 4*f* orbitals. It is noticeable that all calculations were conducted in Orca 6.0.1[11].

Intramolecular energy transfer. With the TD-DFT results, the rates of intramolecular energy transfer (IET) from the excited state (S_1 and T_1) of the ligand to the Tb^{III} and Eu^{III} ions were calculated in the JOYSpectra software[12]. The calculation considered three main mechanisms: the dipole-dipole (W_{d-d}), dipole-multipole (W_{m-d}), and exchange mechanism (W_{ex}), as written in Eqn S4 – S6[13,14,15]. In this case, the overall rates are obtained as a sum among each mechanism, i.e., $W = W_{d-d} + W_{m-d} + W_{ex}$.

$$W_{d-d} = \frac{S_L(1-\sigma_1)^2}{(2J+1)G}\frac{4\pi}{\hbar}\frac{e^2}{R_L^6}\sum_\lambda \Omega_\lambda^{FED}\left\langle\psi'J'\middle\|U^{(\lambda)}\middle\|\psi J\right\rangle^2 F \qquad \text{(S4)}$$

$$W_{m-d} = \frac{S_L}{(2J+1)G}\frac{2\pi e^2}{\hbar}\sum_\lambda(\lambda+1)\frac{\langle r^\lambda\rangle^2}{\left(R_L^{\lambda+2}\right)^2}\left\langle f\middle\|C^{(\lambda)}\middle\|f\right\rangle^2(1 - \sigma_1)^2\left\langle\psi'J'\middle\|U^{(\lambda)}\middle\|\psi J\right\rangle^2 F \qquad \text{(S5)}$$

$$W_{ex} = \frac{(1-\sigma_0)^2}{(2J+1)G}\frac{8\pi}{\hbar}\frac{e^2}{R_L^4}\langle\psi'J'||S||\psi J\rangle^2\sum_m\left\langle\phi\middle\|\textstyle\sum_j\mu_z(j)s_m(j)\middle\|\phi^*\right\rangle^2 F \qquad \text{(S6)}$$

In all equations, R_L represents the distance between the donor and acceptor states, which is obtained from the excited state calculation (Eqn S7). It is noteworthy that R_L considers the individual distance between the atomic center and the Ln^{III}. In this case, c_i is the *i*-th atomic orbital coefficient included in the ligand donor excited states (S_1 or T_1), $R_L(i)$ is the distance between the c_i center to the Ln^{III}, and a_j is the weight of the *j*-th orbital that composes the excited state[16].

$$R_L = \frac{\sum_{i,j} a_j^2 c_i^2 R_L(i)}{\sum_{i,j} a_j^2 c_i^2} \tag{S7}$$

It should be noted that only the FED contribution is considered in Eqn S4[17]. The values of the squared reduced matrix elements, $\langle \psi' J' || U^{(\lambda)} || \psi J \rangle^2$, were taken from Carnall et al[18]. S_L represents the dipole strength of the ligand transition involved in the IET process, with values of 10^{-36} and 10^{-40} esu^2 cm^2 for S_1 and T_1, respectively. $\langle r^{\lambda} \rangle$ represents the 4*f* radial integrals, *G* the ligand degeneracy (G = 1 or 3 for S_1 or T_1, respectively). The reduced matrix elements of Racah's tensor operator are considered in Eqn S5, $\langle f || C^{(\lambda)} || f \rangle$, as well as the shielding factors in Eqn S4 and S6, $(1 - \sigma_k)$, which relies on the overlap integrals between the valence orbitals of the pair Ln-O[19,20]. In the exchange expression, s_m is the spin operator in the ligand and μ_z is the dipole operator (its *z*-component)[13]. $\langle \psi' J' || S || \psi J \rangle$ represents the reduced matrix elements of the spin operator, which were calculated using free ion wavefunctions in the intermediate coupling scheme.

Because of the non-resonant character of the IET, an energy mismatch (*F*) condition between the donor and acceptor states is needed, taking the form of Eqn S8 for the ligand-to-metal energy transfer.

$$F = \frac{G(\delta,T)}{\hbar\gamma_L}\sqrt{\frac{\ln(2)}{\pi}}\, e^{-\left(\frac{\delta}{\hbar\gamma_L}\right)^2 \ln(2)} \quad \text{, with } (\delta,T) = \begin{cases} 1 \; if \; \delta \geq 0 \\ \exp\left(\frac{\delta}{k_B T}\right) if \; \delta < 0 \end{cases} \tag{S8}$$

In this expression, δ represents the energy difference between donor and acceptor states, γ_L is the full width at half maximum of the donor state, assumed here to have a typical value of 4000 cm^{-1} for S_1 and T_1 states. A special case is when $\delta < 0$ because the energy mismatch factor is multiplied by the Boltzmann factor, where k_B is the Boltzmann constant, with value of 0.695 cm^{-1} K^{-1} and *T* the temperature.

Pairwise Tb-Eu energy transfer. The energy transfer rates between a pair of lanthanide ions can be calculated considering the dipole-dipole (W_{d-d}), dipole-quadrupole (W_{d-q}), quadrupole-quadrupole (W_{q-q}) and exchange (W_{ex}) mechanisms[21,22], as shown in Eqn S9 – S12.

$$W_{d-d} = \frac{(1-\sigma_1^D)^2(1-\sigma_1^A)^2}{(2J_D^*+1)(2J_A+1)} \frac{4\pi}{3\hbar} \frac{e^4}{R_{DA}^6} \left(\sum_\lambda \Omega_\lambda^D \langle \psi_D J_D || U^{(\lambda)} || \psi_D^* J_D^* \rangle^2 \right) \times \left(\sum_\lambda \Omega_\lambda^A \langle \psi_A^* J_A^* || U^{(\lambda)} || \psi_A J_A \rangle^2 \right) F \quad \text{(S9)}$$

$$W_{d-q} = \frac{\left(1-\sigma_1^{D,A}\right)^2 \left(1-\sigma_2^{A,D}\right)^2}{(2J_D^*+1)(2J_A+1)} \frac{\pi}{\hbar} \frac{e^4}{R_{DA}^8} \langle f || C^{(\lambda)} || f \rangle^2 \times \left[\left(\sum_\lambda \Omega_\lambda^D \langle \psi_D J_D || U^{(\lambda)} || \psi_D^* J_D^* \rangle^2 \right) \langle r^2 \rangle_A^2 \langle \psi_A^* J_A^* || U^{(\lambda)} || \psi_A J_A \rangle^2 + \left(\sum_\lambda \Omega_\lambda^A \langle \psi_A J_A || U^{(\lambda)} || \psi_A^* J_A^* \rangle^2 \right) \langle r^2 \rangle_D^2 \langle \psi_D^* J_D^* || U^{(\lambda)} || \psi_D J_D \rangle^2 \right] F \quad \text{(S10)}$$

$$W_{q-q} = \frac{(1-\sigma_2^D)^2(1-\sigma_2^A)^2}{(2J_D^*+1)(2J_A+1)} \frac{28\pi}{5\hbar} \frac{e^4}{R_{DA}^{10}} \langle r^2 \rangle_D^2 \langle r^2 \rangle_A^2 \langle f || C^{(\lambda)} || f \rangle^2 \langle \psi_D J_D || U^{(\lambda)} || \psi_D^* J_D^* \rangle^2 \times \langle \psi_A^* J_A^* || U^{(\lambda)} || \psi_A J_A \rangle^2 F \quad \text{(S11)}$$

$$W_{ex} = \frac{2\pi}{\hbar} \left[\left(\frac{e^2}{R_{DA}} \right) \rho_{f-f}^2 \right]^2 F \quad \text{(S12)}$$

In all equations the letters A and D stand for the acceptor (Eu^{III}) and donor (Tb^{III}) ions, respectively. As with the IET case, only the FED mechanism is considered in the intensity parameters for each ion in Eqn S9 – 10, whereas W_{q-q} and W_{ex} are independent of the Ω_λ parameters. ρ_{f-f} is the overlap integral between the 4*f* subshells of the donor (Tb^{III}) and acceptor (Eu^{III}). This quantity is highly reliant on the distance between the ions and can be calculated through the parametric expression in reference [20]. In all mechanisms but the exchange, the shielding factors are crucial, which are determined by Eqn S13, k = 1, 2 and $\beta_{D,A} = \left(1 \pm \rho_{D,A}\right)^{-1}$.

$$\left(1-\sigma_k^{D,A}\right) = \rho_{D,A}\left(2\beta_{D,A}\right)^{k+1} \quad \text{(S13)}$$

As with the IET case, the pairwise Ln-Ln energy transfer is also non resonant, demanding an energy mismatch factor represented in Eqn S14.

$$F = \frac{\ln(2)}{\sqrt{\pi}} \frac{1}{\hbar^2 \gamma_D \gamma_A} \left\{ \left[\left(\frac{1}{\hbar \gamma_D} \right)^2 + \left(\frac{1}{\hbar \gamma_A} \right)^2 \right] \ln(2) \right\}^{-\frac{1}{2}} \times \exp \left[\frac{1}{4} \frac{\left(\frac{2\delta}{(\hbar \gamma_D)^2} \ln(2) \right)^2}{\left[\left(\frac{1}{\hbar \gamma_D} \right)^2 + \left(\frac{1}{\hbar \gamma_A} \right)^2 \right] \ln(2)} - \left(\frac{\delta}{\hbar \gamma_D} \right)^2 \ln(2) \right] \quad \text{(S14)}$$

In this equation, $\hbar\gamma_D$ and $\hbar\gamma_A$ corresponds to the bandwidth at half-maximum of the donor and acceptor transitions, respectively. Since Ln^{III} presents narrow transitions, we considered a value of 350 cm^{-1} and 350 cm^{-1} for the donor and acceptor, respectively.

Tb^{III}/Eu^{III} distribution. After the calculation of Tb^{III}-Eu^{III} pairwise ET, a Monte Carlo type distribution of the coordinates of Tb^{III} and Eu^{III} ions is simulated via a homemade program written in C language. The program was developed for reference [23] by Carneiro Neto, *et al.*, and it is available under request. The unit cell of the compounds, retrieved from the single-crystal X-ray data was expanded to a supercell (25 × 25 × 25) containing 16,002 host sites, each of which can be randomly replaced by Tb^{III} or Eu^{III} until reaching the desired fraction (in %). In this sense, the occurrence of Tb-Eu pair as a function of the distance and concentration $1 - x$ of Eu^{III} across the compound is obtained, enabling to average the forward ($\langle W \rangle^f$) energy transfer rates (Eqn S15).

$$\langle W \rangle^f = \sum_i \langle W \rangle_i^f = (1-x)x \left(\sum_i O_i(1-x) W_i^f \right) \quad \text{(S15)}$$

In these equations, $\langle W \rangle_i^f$ represents the forward and backward energy transfer for the ith Tb−Eu distance. The acceptor Tb^{III} and the donor Eu^{III} fractions are represented by $1 - x$ and x, respectively. The occurrence coefficients (O_i) relate to the formation of a Tb^{III}/Eu^{III} pair at distance *R(i)*, regarding the acceptor in the case of forward ET ($\langle W \rangle^f$) and for the donor in the case of backward ET ($\langle W \rangle^b$), whose definitions lie in Eqn S16 – S17.

$$O_i(1-x) = \frac{\mathcal{N}(i)}{s(1-x)} \quad (S16)$$

$$O_i(x) = \frac{\mathcal{N}(i)}{s(x)} \quad (S17)$$

For these coefficients, $\mathcal{N}(i)$ is the counting of Nd−Yb pairs at distance R(i) and *s* is the number of host sites in the primary compound (equals 16,200 possibilities in the 25 × 25 × 25 supercell), Table S7.

Table S7. Occurrence coefficients and individual rates from the pairwise Tb^{III}-to-Eu^{III} ET calculated for different distances found in a cutoff of 13 Å.

$R_{Tb\text{-}Eu}$ *(i) / Å*	W_i	$O_i(x)$	$O_i(1-x)$
	**[$Eu_{0.75}Tb_{0.25}(tfa)_3$(μ-dppeo)]**		
8.2960	9.312×10^{2}	0.706	0.235
8.5030	7.279×10^{2}	0.670	0.223
11.628	3.190×10^{1}	0.687	0.229
12.283	2.002×10^{1}	0.694	0.216
	**[$Eu_{0.25}Tb_{0.75}(tfa)_3$(μ-dppeo)]**		
8.2960	9.312×10^{2}	0.242	0.725
8.5030	7.279×10^{2}	0.226	0.678
11.628	3.190×10^{1}	0.230	0.691
12.283	2.002×10^{1}	0.210	0.631

Table S8. Singlet (S_1) and triplet (T_1) state energies calculated via the method described in the computational procedure, as well as the distance (R_L) between the centroid donor (ligand) and the acceptor (Ln^{III}).

	**[$Ln(tfa)_3$(μ-dppeo)]**	**[$Ln(hfa)_3$(μ-dppeo)]**	**[$Ln(tfa)_3$(μ-dppbo)]**	**[$Ln(hfa)_3$(μ-dppbo)]**
**E(S_1) / cm^{-1}**	36,737	37,725	36,637	37,435
**E(T_1) / cm^{-1}**	23,628	23,815	22,455	23,655
**R_L(S_1) / Å**	4.38	4.41	4.28	4.31
**R_L(T_1) / Å**	4.12	4.18	4.01	4.15

Table S9. Forward ligand→Eu^{III} IET rates (in units of s^{-1}) for the $[Eu(tfa)_3(\mu\text{-dppeo})]_n$ as example calculated at 300 K. W^S and W^T are the total energy transfer via S_1 and T_1 states. W_{d-d}, W_{d-q}, W_{q-q}, W_{ex}, and W_{md-md} are the dipole-dipole, dipole-quadrupole, quadrupole-quadrupole, exchange, and magnetic dipole-magnetic dipole mechanism contributions.

Pathway	Donor	Acceptor	W (%)	δ	W	W_{d-d}	W_{d-m}	W_{ex}
7	$S_1 \to S_0$	$^7F_0 \to {}^5D_1$	0.456	17710	1.512×10^3	0	0	$1.512\ 10^3$
10	$S_1 \to S_0$	$^7F_0 \to {}^5L_6$	5.717	11412	1.897×10^4	1.888×10^4	9.232×10^1	0
15	$S_1 \to S_0$	$^7F_0 \to {}^5G_6$	5.190	9985	1.722×10^4	1.713×10^4	8.381×10^1	0
18	$S_1 \to S_0$	$^7F_0 \to {}^5D_4$	3.559	9151	1.181×10^4	5.822×10^3	5.987×10^3	0
26	$S_1 \to S_0$	$^7F_1 \to {}^5D_0$	0.004	19816	1.403×10^1	0	0	1.403×10^1
27	$S_1 \to S_0$	$^7F_1 \to {}^5D_1$	0.004	18082	1.388×10^1	4.624×10^{-4}	1.382×10^1	5.805×10^{-2}
28	$S_1 \to S_0$	$^7F_1 \to {}^5D_2$	0.103	15626	3.416×10^2	0	0	3.416×10^2
29	$S_1 \to S_0$	$^7F_1 \to {}^5D_3$	0.831	12754	2.756×10^3	1.390×10^1	2.742×10^3	0
30	$S_1 \to S_0$	$^7F_1 \to {}^5L_6$	0.155	11784	5.155×10^2	5.129×10^2	2.509	0
31	$S_1 \to S_0$	$^7F_1 \to {}^5L_7$	0.846	10752	2.808×10^3	2.794×10^3	1.367×10^1	0
32	$S_1 \to S_0$	$^7F_1 \to {}^5G_2$	78.004	10717	2.588×10^5	0	0	2.588×10^5
33	$S_1 \to S_0$	$^7F_1 \to {}^5G_3$	4.112	10487	1.364×10^4	1.358×10^2	1.351×10^4	0
35	$S_1 \to S_0$	$^7F_1 \to {}^5G_6$	0.329	10357	1.091×10^3	1.086×10^3	5.310	0
36	$S_1 \to S_0$	$^7F_1 \to {}^5G_5$	0.689	10346	2.285×10^3	2.222×10^3	6.330×10^1	0
				W^S	**2.890×10^5**			
7	$T_1 \to S_0$	$^7F_0 \to {}^5D_1$	89.074	4601	3.142×10^8	0	0	3.142×10^8
26	$T_1 \to S_0$	$^7F_1 \to {}^5D_0$	9.034	6707	3.187×10^7	0	0	3.187×10^7
27	$T_1 \to S_0$	$^7F_1 \to {}^5D_1$	0.005	4973	1.877×10^4	1.080×10^{-2}	3.648×10^2	1.841×10^4
28	$T_1 \to S_0$	$^7F_1 \to {}^5D_2$	1.887	2517	6.656×10^6	0	0	6.656×10^6
				W^T	**3.555×10^8**			

Table S10. Backward Ligand→Eu^{III} IET rates (in units of s^{-1}) for the $[Eu(tfa)_3(\mu\text{-dppeo})]_n$ as example calculated at 300 K. W^S and W^T are the total energy transfer via S_1 and T_1 states. W_{d-d}, W_{d-q}, W_{q-q}, W_{ex}, and W_{md-md} are the dipole-dipole, dipole-quadrupole, quadrupole-quadrupole, exchange, and magnetic dipole-magnetic dipole mechanism contributions.

Pathway	Donor	Acceptor	W (%)	δ	W	W_{d-d}	W_{d-m}	W_{ex}
10	$^5L_6 \rightarrow ^7F_0$	$S_0 \rightarrow S_1$	0.001	-11412	2.123×10^{-21}	2.112×10^{-21}	1.033×10^{-23}	0
15	$^5G_6 \rightarrow ^7F_0$	$S_0 \rightarrow S_1$	1.312	-9985	1.885×10^{-18}	1.876×10^{-18}	9.177×10^{-21}	0
18	$^5D_4 \rightarrow ^7F_0$	$S_0 \rightarrow S_1$	72.715	-9151	1.045×10^{-16}	5.153×10^{-17}	5.298×10^{-17}	0
31	$^5L_7 \rightarrow ^7F_1$	$S_0 \rightarrow S_1$	0.069	-10752	9.910×10^{-20}	9.862×10^{-20}	4.824×10^{-22}	0
32	$^5G_2 \rightarrow ^7F_1$	$S_0 \rightarrow S_1$	22.574	-10717	3.244×10^{-17}	0	0	3.244×10^{-17}
33	$^5G_3 \rightarrow ^7F_1$	$S_0 \rightarrow S_1$	2.579	-10487	3.706×10^{-18}	3.689×10^{-20}	3.669×10^{-18}	0
35	$^5G_6 \rightarrow ^7F_1$	$S_0 \rightarrow S_1$	0.208	-10357	2.988×10^{-19}	2.974×10^{-19}	1.455×10^{-21}	0
36	$^5G_5 \rightarrow ^7F_1$	$S_0 \rightarrow S_1$	0.543	-10346	7.800×10^{-19}	7.584×10^{-19}	2.161×10^{-20}	0
				W_b^S	**1.223×10^{-16}**			
26	$^5D_0 \rightarrow ^7F_1$	$S_0 \rightarrow T_1$	0.001	-6707	5.061×10^{-6}	0	0	5.061×10^{-6}
29	$^5D_3 \rightarrow ^7F_1$	$S_0 \rightarrow T_1$	0.001	355	4.421×10^{2}	1.972	4.401×10^{2}	0
32	$^5G_2 \rightarrow ^7F_1$	$S_0 \rightarrow T_1$	99.98	2392	6.900×10^{7}	0	0	6.900×10^{7}
				W_b^T	**6.959×10^{7}**			

Table S11. Forward Ligand→Tb^{III} IET rates (in units of s^{-1}) for the $[Eu(tfa)_3(\mu\text{-dppeo})]_n$ as example calculated at 300 K. W^S and W^T are the total energy transfer via S_1 and T_1 states. W_{d-d}, W_{d-q}, W_{q-q}, W_{ex}, and W_{md-md} are the dipole-dipole, dipole-quadrupole, quadrupole-quadrupole, exchange, and magnetic dipole-magnetic dipole mechanism contributions.

Pathway	Donor	Acceptor	W (%)	δ	W	W_{d-d}	W_{d-m}	W_{ex}
8	S_1→S_0	$^7F_6$→$^5G_6$	0.614	10314	2.668×10^7	2.340×10^4	1.721×10^5	2.648×10^7
10	S_1→S_0	$^7F_6$→$^5G_5$	0.138	8970	5.992×10^6	8.596×10^3	3.959×10^5	5.587×10^6
12	S_1→S_0	$^7F_6$→$^5G_4$	0.001	8450	5.693×10^4	7.971×10^3	4.896×10^4	0
13	S_1→S_0	$^7F_6$→$^5L_9$	0.001	8329	4.709×10^4	4.570×10^4	1.389×10^3	0
15	S_1→S_0	$^7F_6$→$^5L_8$	0.001	7547	3.866×10^4	3.837×10^4	2.810×10^2	0
16	S_1→S_0	$^7F_6$→$^5L_7$	0.016	7280	6.740×10^5	2.425×10^4	6.495×10^5	1.879×10^2
18	S_1→S_0	$^7F_6$→$^5L_6$	0.113	7067	4.924×10^6	0	0	4.924×10^6
21	S_1→S_0	$^7F_6$→$^5H_7$	0.317	5358	1.378×10^7	0	0	1.378×10^7
22	S_1→S_0	$^7F_6$→$^5H_6$	4.671	3846	2.031×10^8	0	0	2.031×10^8
25	S_1→S_0	$^7F_6$→$^5F_5$	15.783	1803	6.862×10^8	0	0	0
30	S_1→S_0	$^7F_6$→$^5I_7$	0.803	148	3.492×10^7	0	0	0
33	S_1→S_0	$^7F_6$→$^5I_6$	0.177	-861	7.710×10^6	0	0	0
35	S_1→S_0	$^7F_6$→$^5I_5$	0.002	-1249	9.640×10^4	0	0	0
47	S_1→S_0	$^7F_5$→$^5G_6$	0.014	12362	6.179×10^5	3.432e+02	6.780×10^4	5.498×10^5
49	S_1→S_0	$^7F_5$→$^5G_5$	0.165	11018	7.157×10^6	8.053e+02	6.626×10^3	7.149×10^6
51	S_1→S_0	$^7F_5$→$^5G_4$	0.035	10498	1.525×10^6	4.164e+02	5.364×10^4	1.471×10^6
55	S_1→S_0	$^7F_5$→$^5L_7$	0.001	9328	6.238×10^4	3.658e+03	5.872×10^4	0
57	S_1→S_0	$^7F_5$→$^5L_6$	0.004	9115	1.887×10^5	0	0	1.887×10^5
61	S_1→S_0	$^7F_5$→$^5H_6$	0.301	5894	1.306×10^7	0	0	1.306×10^7
62	S_1→S_0	$^7F_5$→$^5H_5$	5.683	5018	2.471×10^8	0	0	2.471×10^8
63	S_1→S_0	$^7F_5$→$^5H_4$	16.416	4446	7.137×10^8	0	0	7.137×10^8
64	S_1→S_0	$^7F_5$→$^5F_5$	11.774	3851	5.119×10^8	0	0	5.119×10^8
67	S_1→S_0	$^7F_5$→$^5F_4$	40.881	3411	1.777×10^9	0	0	1.777×10^9
72	S_1→S_0	$^7F_5$→$^5I_6$	1.947	1187	8.463×10^7	0	0	8.463×10^7
73	S_1→S_0	$^7F_5$→$^5I_4$	0.002	1177	8.525×10^4	0	0	8.525×10^4
74	S_1→S_0	$^7F_5$→$^5I_5$	0.138	799	5.999×10^6	0	0	5.999×10^6
				W^S	**4.346×10^9**			
6	T_1→S_0	$^7F_6$→$^5D_4$	0.001	3184	3.405×10^2	0	3.394×10^2	0
8	T_1→S_0	$^7F_6$→$^5G_6$	0.003	-2795	1.717×10^3	1.118×10^{-4}	9.301×10^{-4}	1.717×10^3
45	T_1→S_0	$^7F_5$→$^5D_4$	88.925	5232	5.767×10^7	1.096	2.996×10^3	5.767×10^7
47	T_1→S_0	$^7F_5$→$^5G_6$	11.024	-747	7.150×10^6	3.288e-01	7.341e+01	7.150×10^6
				W^T	**6.499×10^7**			

Table S12. Backward Ligand→Tb^{III} IET rates (in units of s^{-1}) for the [Eu(tfa)$_3$(μ-dppeo)]$_n$ as example calculated at 300 K. W^S and W^T are the total energy transfer via S$_1$ and T$_1$ states. W_{d-d}, W_{d-q}, W_{q-q}, W_{ex}, and W_{md-md} are the dipole-dipole, dipole-quadrupole, quadrupole-quadrupole, exchange, and magnetic dipole-magnetic dipole mechanism contributions.

Pathway	Donor	Acceptor	W (%)	δ	W	W_{d-d}	W_{d-m}	W_{ex}
25	$^5F_5 \to ^7F_6$	$S_0 \to S_1$	0.024	-1803	1.350e+05	0	0	1.350e+05
30	$^5I_5 \to ^7F_6$	$S_0 \to S_1$	2.663	-148	1.482e+07	0	0	1.482e+07
33	$^5I_6 \to ^7F_6$	$S_0 \to S_1$	88.338	861	4.914e+08	0	0	4.914e+08
38	$^5K_5 \to ^7F_6$	$S_0 \to S_1$	0.187	4597	1.042×10^6	0	0	1.042×10^6
				W_b^S	**5.563×10^8**			
8	$^5G_6 \to ^7F_6$	$S_0 \to T_1$	48.887	2795	1.237×10^9	8.052×10^1	6.699×10^2	1.237×10^9
10	$^5G_5 \to ^7F_6$	$S_0 \to T_1$	2.649	4139	6.701×10^7	7.595	3.955×10^2	6.701×10^7
18	$^5L_6 \to ^7F_6$	$S_0 \to T_1$	0.227	6042	5.754×10^6	0	0	5.754×10^6
21	$^5H_7 \to ^7F_6$	$S_0 \to T_1$	0.079	7751	2.003×10^6	0	0	2.003×10^6
22	$^5H_6 \to ^7F_6$	$S_0 \to T_1$	0.242	9263	6.116×10^6	0	0	6.116×10^6
25	$^5F_5 \to ^7F_6$	$S_0 \to T_1$	0.095	11306	2.399×10^6	0	0	2.399×10^6
30	$^5I_7 \to ^7F_6$	$S_0 \to T_1$	0.001	12961	1.366×10^4	0	0	1.366×10^4
33	$^5I_6 \to ^7F_6$	$S_0 \to T_1$	0.003	13970	7.054×10^4	0	0	7.054×10^4
47	$^5G_6 \to ^7F_5$	$S_0 \to T_1$	8.793	747	2.224×10^8	1.023×10^1	2.284×10^3	2.224×10^8
49	$^5G_5 \to ^7F_5$	$S_0 \to T_1$	29.361	2091	7.428×10^8	6.164	5.736×10^1	7.428×10^8
57	$^5L_6 \to ^7F_5$	$S_0 \to T_1$	0.076	3994	1.911×10^6	0	0	1.911×10^6
61	$^5H_6 \to ^7F_5$	$S_0 \to T_1$	0.135	7215	3.409×10^6	0	0	3.409×10^6
62	$^5H_5 \to ^7F_5$	$S_0 \to T_1$	1.114	8091	2.817×10^7	0	0	2.817×10^7
63	$^5H_4 \to ^7F_5$	$S_0 \to T_1$	2.053	8663	5.194×10^7	0	0	5.194×10^7
64	$^5F_5 \to ^7F_5$	$S_0 \to T_1$	0.613	9258	1.551×10^7	0	0	1.551×10^7
				W_b^T	**2.529×10^9**			

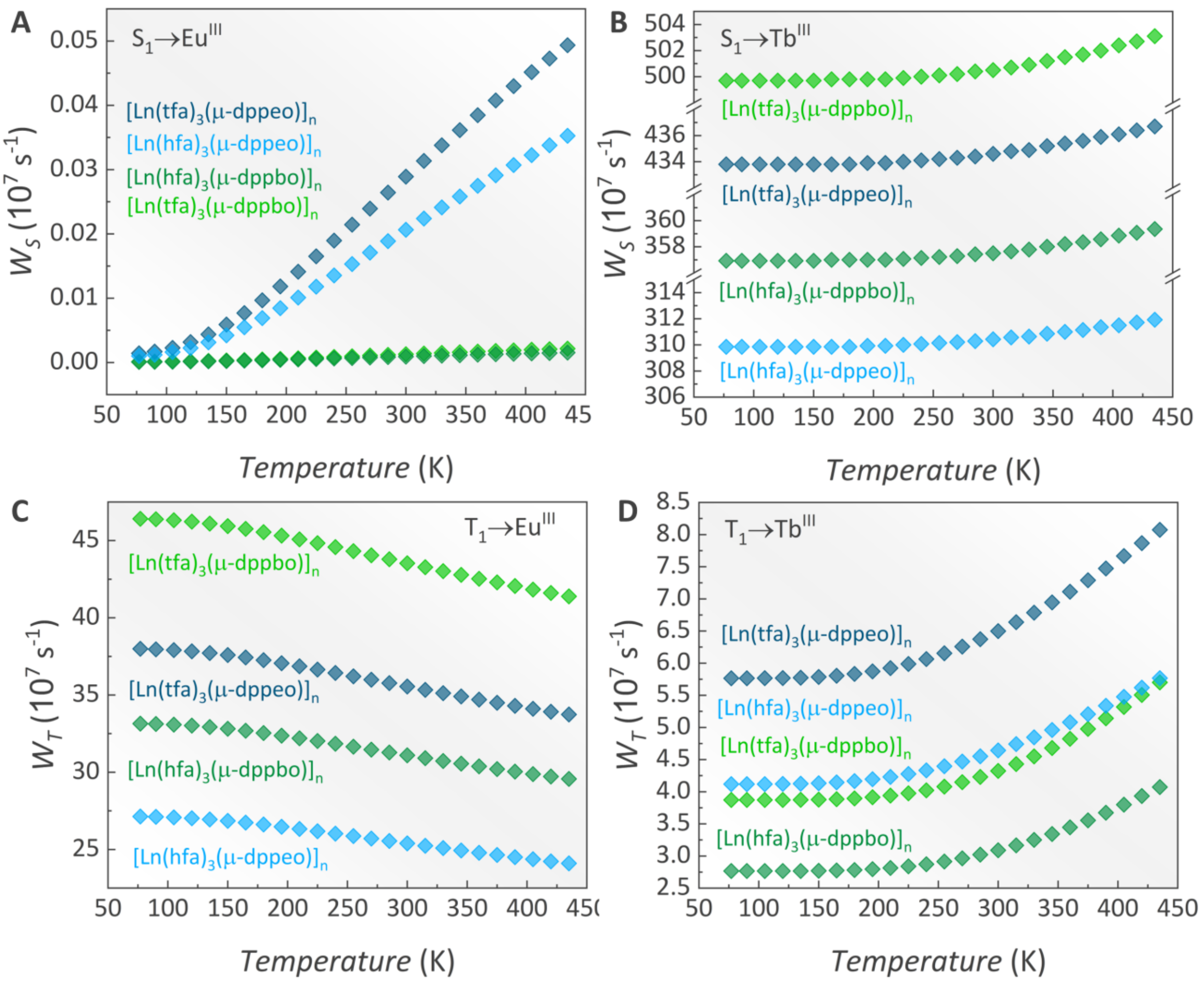

Figure S17. Temperature dependent ligand-to-Ln^{III} forward-IET for the coordination polymer focusing on (A) S_1→Eu^{III}, (B) S_1→Tb^{III}, (C) T_1→Eu^{III} and T_1→Tb^{III} (D) processes.

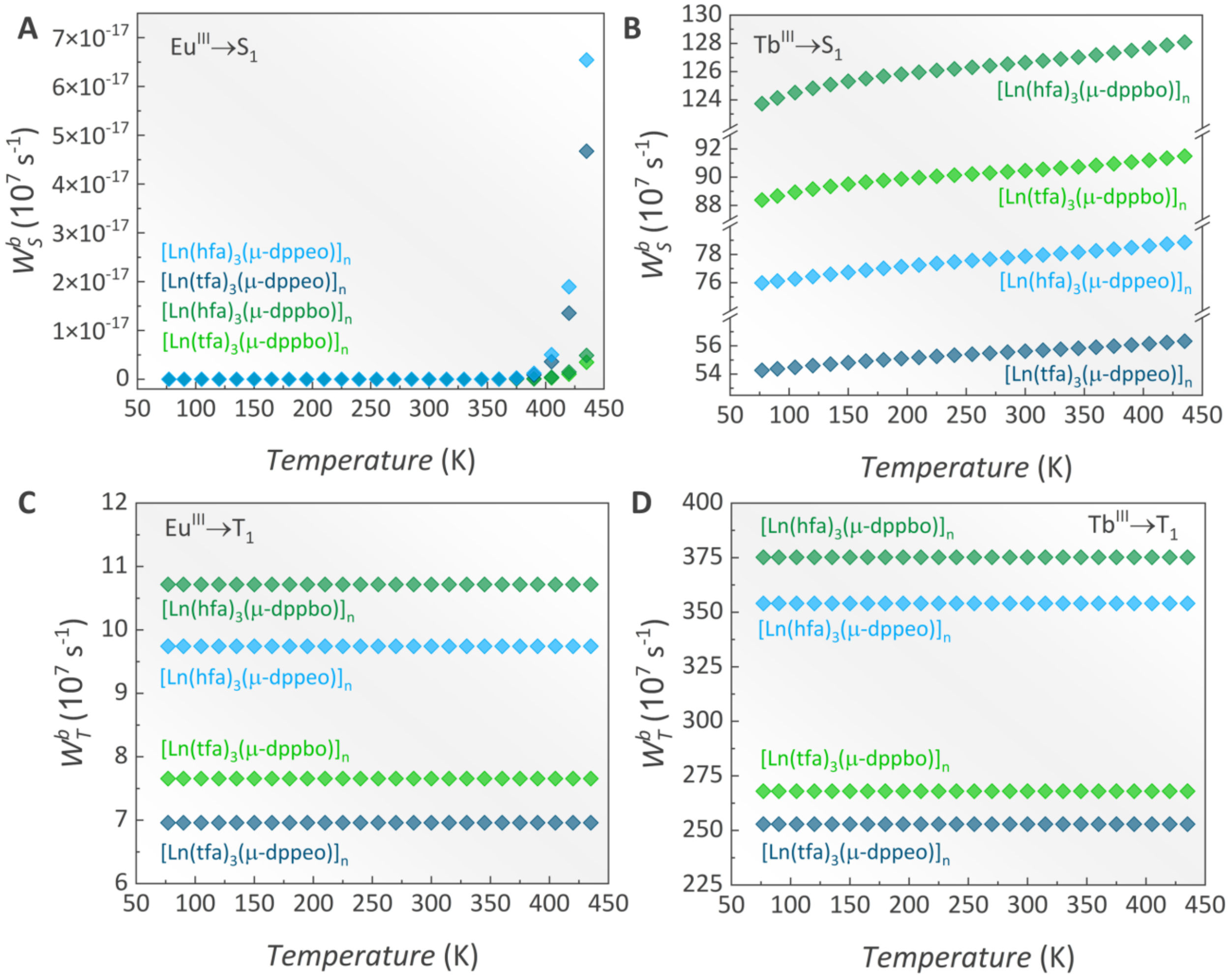

Figure S18. Temperature dependent Ln^{III}-to-ligand back-IET rates for the coordination polymer focusing on (A) $Eu^{III} \rightarrow S_1$, (B) $Tb^{III} \rightarrow S_1$, (C) $Eu^{III} \rightarrow T_1$ and $Tb^{III} \rightarrow T_1$ (D) processes.

Table S13. Tb^{III}-to-Eu^{III} energy transfer rates at 300 K considering the intramolecular distance ($R_{Tb–Eu}$ = 8.296 Å) for $[Ln(tfa)_3(\mu\text{-dppeo})]_n$. W_{d-d}, W_{d-q}, W_{q-q}, W_{ex}, and W_{md-md} are the dipole-dipole, dipole-quadrupole, quadrupole-quadrupole, exchange, and magnetic dipole-magnetic dipole mechanism contributions. All rates in s^{-1}, print threshold of 0.0005%

Path	Donor	Acceptor	W (%)	δ / cm^{-1}	W_{dd}	W_{dq}	W_{qq}	W_{ex}	W
**17**	$^5D_4 \rightarrow ^7F_6$	$^7F_1 \rightarrow ^5D_1$	0.0014	1789	3.153×10^{-9}	3.825×10^{-5}	4.013×10^{-2}	6.662×10^{-32}	1.354×10^{-2}
**53**	$^5D_3 \rightarrow ^7F_6$	$^7F_1 \rightarrow ^5G_3$	0.0009	-14	1.285×10^{-4}	2.55×10^{-2}	0	5.329×10^{-28}	8.637×10^{-3}
**65**	$^5G_6 \rightarrow ^7F_6$	$^7F_0 \rightarrow ^5L_6$	0.0044	1098	1.27×10^{-2}	5.004×10^{-2}	0	1.883×10^{-29}	4.179×10^{-2}
**70**	$^5G_6 \rightarrow ^7F_6$	$^7F_0 \rightarrow ^5G_6$	0.0049	-329	1.414×10^{-2}	5.571×10^{-2}	0	8.666×10^{-29}	4.652×10^{-2}
**116**	$^5L_{10} \rightarrow ^7F_6$	$^7F_1 \rightarrow ^5G_5$	0.0026	580	7.465×10^{-2}	0	0	2.202×10^{-28}	2.516×10^{-2}
**130**	$^5G_5 \rightarrow ^7F_6$	$^7F_0 \rightarrow ^5G_6$	0.0013	1015	7.163×10^{-4}	1.762×10^{-2}	0	3.092×10^{-29}	1.221×10^{-2}
**133**	$^5G_5 \rightarrow ^7F_6$	$^7F_0 \rightarrow ^5D_4$	0.0037	181	2.027×10^{-3}	4.987×10^{-2}	0	5.198×10^{-28}	3.456×10^{-2}
**143**	$^5G_5 \rightarrow ^7F_6$	$^7F_1 \rightarrow ^5G_3$	0.0016	1517	1.223×10^{-6}	2.729×10^{-4}	4.459×10^{-2}	8.482×10^{-31}	1.512×10^{-2}
**1021**	$^5D_4 \rightarrow ^7F_5$	$^7F_0 \rightarrow ^5D_0$	47.50	1103	7.597×10^{-6}	9.85×10^{-2}	6.672×10^{2}	1.825×10^{-29}	4.436×10^{2}
**1037**	$^5D_4 \rightarrow ^7F_5$	$^7F_1 \rightarrow ^5D_1$	46.72	-259	1.477×10^{-4}	1.915×10^{-1}	1.294×10^{3}	1.362×10^{-28}	4.363×10^{2}
**1069**	$^5D_3 \rightarrow ^7F_5$	$^7F_1 \rightarrow ^5D_3$	1.263	205	2.69×10^{-4}	1.170×10^{-1}	3.486×10^{1}	5.063×10^{-28}	1.179×10^{1}
**1099**	$^5G_6 \rightarrow ^7F_5$	$^7F_1 \rightarrow ^5D_3$	3.958	392	3.63×10^{-4}	1.793×10^{-1}	1.095×10^{2}	3.692×10^{-28}	3.696×10^{2}
**1100**	$^5G_6 \rightarrow ^7F_5$	$^7F_1 \rightarrow ^5L_6$	0.0006	-578	1.765×10^{-4}	1.826×10^{-2}	0	1.386×10^{-29}	6.213×10^{-3}
**1223**	$^5G_4 \rightarrow ^7F_5$	$^7F_1 \rightarrow ^5G_3$	0.5239	-11	1.433×10^{-4}	3.822×10^{-2}	1.448×10^{1}	5.408×10^{-28}	4.892
**1226**	$^5G_4 \rightarrow ^7F_5$	$^7F_1 \rightarrow ^5G_5$	0.0024	-152	9.811×10^{-4}	6.687×10^{-2}	0	2.577×10^{-28}	2.287×10^{-2}
**1253**	$^5L_9 \rightarrow ^7F_5$	$^7F_1 \rightarrow ^5G_3$	0.0032	110	4.52×10^{-4}	8.97×10^{-2}	0	5.511×10^{-28}	3.038×10^{-2}
				W_i	9.312×10^{2}				

Supplementary note S7 – Luminescence thermometry

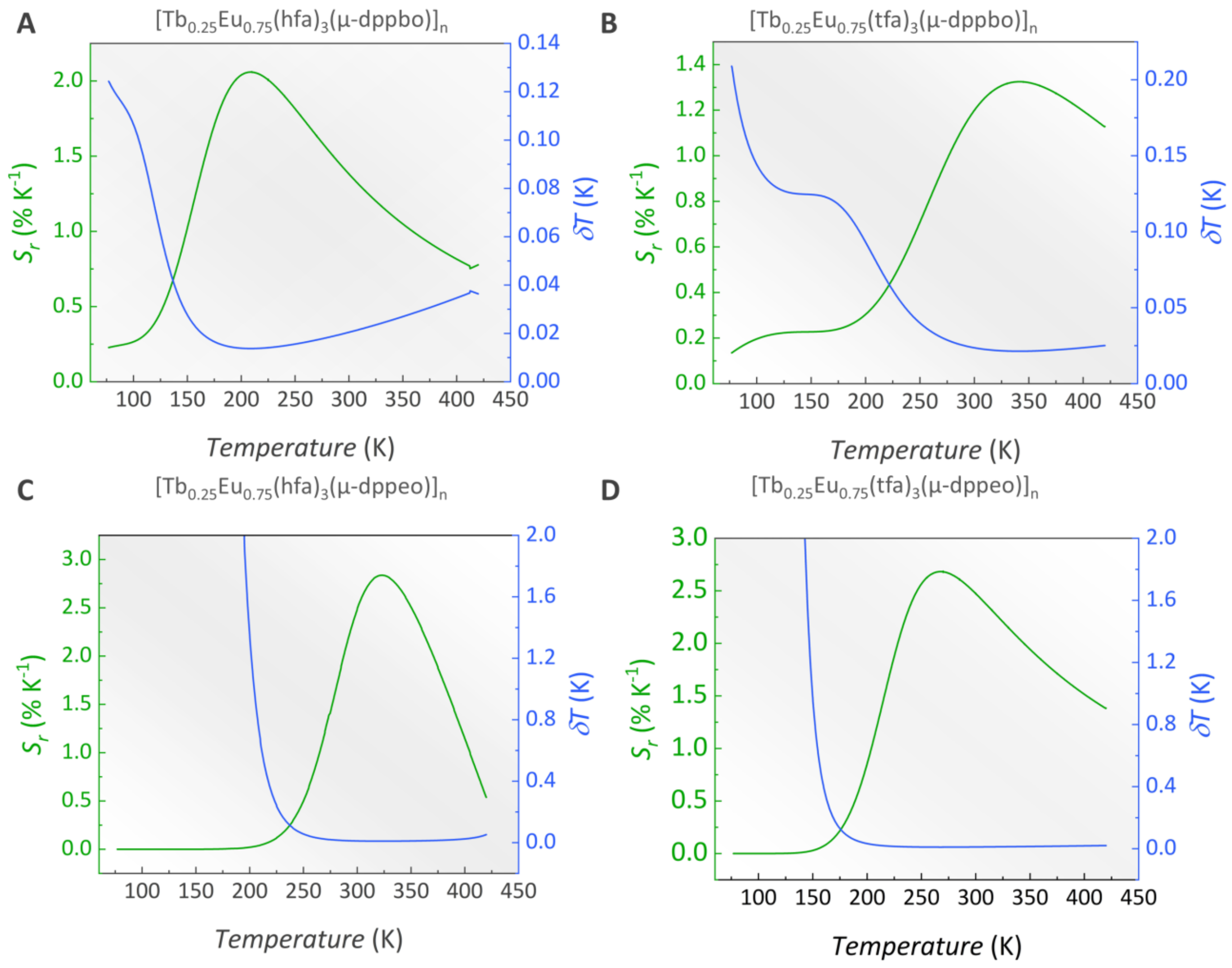

Figure S19. Dependence of the relative thermal sensitivity (S_r) and uncertainty (δT) on the temperature for (A) $[Tb_{0.25}Eu_{0.75}(hfa)_3(\mu\text{-dppbo})]_n$, (B) $[Tb_{0.25}Eu_{0.75}(tfa)_3(\mu\text{-dppbo})]_n$, (C) $[Tb_{0.25}Eu_{0.75}(hfa)_3(\mu\text{-dppeo})]_n$, and (D) $[Tb_{0.25}Eu_{0.75}(tfa)_3(\mu\text{-dppeo})]_n$.

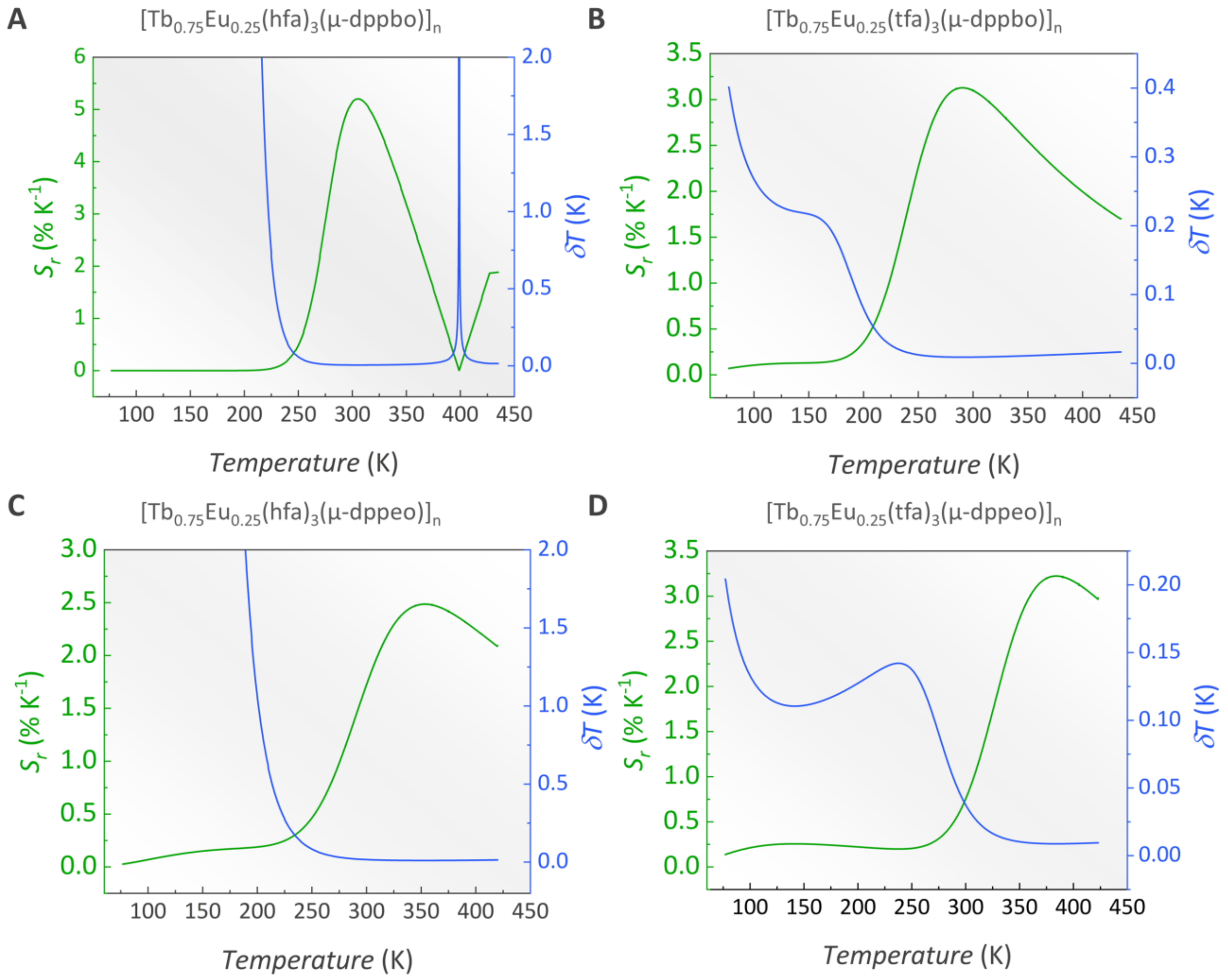

Figure S20. Dependence of the relative thermal sensitivity (S_r) and uncertainty (δT) on the temperature for (A) $[Tb_{0.75}Eu_{0.25}(hfa)_3(\mu\text{-}dppbo)]_n$, (B) $[Tb_{0.75}Eu_{0.25}(tfa)_3(\mu\text{-}dppbo)]_n$, (C) $[Tb_{0.75}Eu_{0.25}(hfa)_3(\mu\text{-}dppeo)]_n$, and (D) $[Tb_{0.75}Eu_{0.25}(tfa)_3(\mu\text{-}dppeo)]_n$.

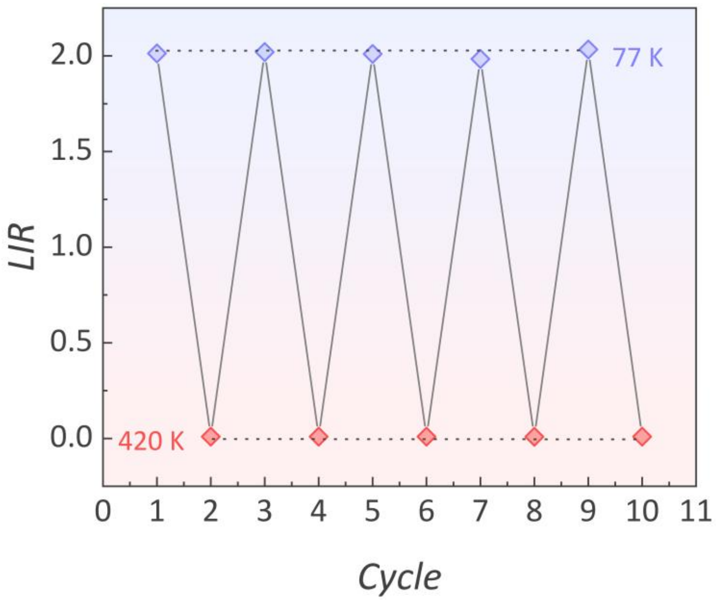

Figure S21. LIR recorded in 10 heating–cooling temperature cycles between 77 and 420 K for $[Tb_{0.75}Eu_{0.25}(hfa)_3(\mu\text{-dppbo})]_n$.

Supplementary references

[1] Lima, D. A.; Bispo-Jr, A. G.; Galico, D. A.; Coelho, S. F. N.; Araujo Neto, J. H.; Ellena, J. A.; Petiote, L.; Mazali, I. O.; Sigoli, F. A.; Tuning the thermometric features in 1D luminescent Eu^{III} and Tb^{III} coordination polymers through different bridge phosphine oxide ligands. *Inorg. Chem.* **2023**, 62, 17, 6808–6816.

[2] de Andrade, S. A.; Bispo-Jr, A. G.; Simoni, D. A.; Ellena, J.; de Araujo Neto, J. H.; Mazali, I. O.; Sigoli, F. A.; The role of terminal and bridge ligands in the molecular upconversion of lanthanide(III) 1D coordination polymers. *J. Mater. Chem. C* **2025**, 13, 1207-1214.

[3] G.M. Sheldrick, SHELXT-Integrated Space-Group and Crystal-Structure Determination. *Acta Cryst.* 2025, A71, 3-8.

[4] Brites, C. D. S.; Millán, A.; Carlos, L. D.; Chapter 281 - Lanthanides in Luminescent Thermometry. In: Handbook on the Physics and Chemistry of Rare Earths; Bunzli, J-C.; Pecharsky, V. Eds.; Elsevier: Amsterdam, 2016; 49, 339-427.

[5] Adamo, C.; Cossi, M.; Barone, V. An accurate density functional method for the study of magnetic properties: the PBE0 model. *J. Mol. Struc. Theochem.* **1999**, 493, 145 – 157.

[6] Caldeweyher, E.; Mewes, J-M.; Ehlert, S.; Grimme, S. Extension and evaluation of the D4 London-dispersion model for periodic systems. *Phys. Chem. Chem. Phys.* **2020**, 22, 8499 – 8512.

[7] Weigend, F.; Ahlrichs, R. Balanced basis set of split valence, triple zeta, valence and quadruple zeta valence quality for H to Rn: Design and assessment accuracy. *Phys. Chem. Chem. Phys.* **2005**, 7, 3297 – 3305.

[8] Yang, J.; Falleta, S.; Pasquarello, A. Range-separated hybrid functionals for accurate prediction of band gaps of extended systems. *npj Comput. Mat.* **2023**, 9, 108.

[9] Dolg, M.; Stoll, H.; Savin, A.; Preuss, H. Energy-adjusted pseudopotentials for the rare earth elements. *Theor. Chim. Acta* **1989**, 75, 173 – 194.

[10] Dolg, M.; Stoll, H.; Savin, A.; Preuss, H. A combination of quasirelativistic pseudopotential and ligand field calculations for lanthanoid compounds. *Theor. Chim. Acta* **1993**, 85, 441 – 450.

[11] Neese, F. A perspective on the future of quantum chemical software: the example of the ORCA program package. *Faraday Discuss.* **2024**, 254, 295 – 314.

[12] Moura Jr., R. T.; Carneiro Neto, A. N.; Aguiar, E. C.; Santos-Jr., C. V.; de Lima, E. M.; Faustino, W. M.; Teotonio, E. E. S.; Felinto, H. B.; Felinto, M. C. F. C.; Ferreira, R. A. S.; Carlos, L. D.; Longo, R. L.; Malta, O. L. JOYSpectra: A web platform for luminescence of lanthanides, *Opt. Mater. X* **2021**, 11, 100080.

[13] Malta, O. L. Ligand-rare-earth ion energy transfer in coordination compounds. A theoretical approach. *J. Lumin.* **1997**, 71, 229.

[14] Malta, O. L.; Gonçalves e Silva, F. R. A theoretical approach to intramolecular energy transfer and emission quantum yields in coordination compounds of rare earth ions. *Spectrochim. Acta Part A Mol. Biomol. Spectrosc.* **1998**, 54, 1593.

[15] Longo, R.; Gonçalves e Silva, F. R.; Malta, O. L. A theoretical study of the energy-transfer process in $[Eu\subset bpy.bpy.bpy]^{3+}$ cryptates: a ligand-to-metal charge-transfer state? *Chem. Phys. Lett.* **2000**, 328, 67.

[16] Moura Jr, R. T.; Quintano, M.; Santos-Jr, C. V.; Albuquerque, V. A. C. A.; Aguar, E. C.; Kraka, E.; Carneiro Neto, A. N. Featuring a new computational protocol for the estimation of intensity and overall quantum yield in lanthanide chelates with applications to Eu(III) mercapto-triazole Schiff base ligands. *Opt. Mat. X* **2022**, 16, 100216.
[17] Kushida, T. Energy Transfer and Cooperative Optical Transitions in Rare-Earth Doped Inorganic Materials. I. Transition Probability Calculation. *J. Phys. Soc. Jpn.* **1973**, 34, 1318 – 1326.
[18] W. T. Carnall, H. Crosswhite, H. M. Crosswhite, Energy level structure and transition probabilities in the spectra of the trivalent lanthanides in LaF3" Argonne, IL, United States, **1978**, p. 38.
[19] Edvardsson, S.; Klintenberg, M. Role of the electrostatic model in calculating rare-earth crystal-field parameters. *J. Alloys and Compd.* **1998**, 257 – 277, 230 – 233.
[20] Carneiro Neto, A. N.; Moura Jr, R. T. Overlap integrals and excitation energies calculations in trivalent lanthanides 4*f* orbitals in pairs Ln-L (L = Ln, N, O, F, P, S, Cl, Se, Br, and I). *Chem. Phys. Lett.* **2020**, 757, 137884.
[21] Malta, O. L. Mechanisms of non-radiative energy transfer involving lanthanide ions revisited. *J. Non-Crystal. Sol.* **2008**, 354, 4770.
[22] Carneiro Neto, A. N.; Moura Jr, R. T.; Shyichuk, A.; Paterlini, V.; Piccinelli, F.; Bettinelli, M.; Malta, O. L. Theoretical and Experimental Investigation of the $Tb^{3+} \rightarrow Eu^{3+}$ Energy Transfer Mechanisms in Cubic $A_3Tb_{0.90}Eu_{0.10}(PO_4)_3$ (A = Sr, Ba) Materials, *J. Phys. Chem. C* **2020**, 124, 10105.
[23] Carneiro Neto, A. N.; Moura Jr., R. T.; Coelho, J. A. A.; Silva-Jr, M. E.; Costa, J. L.; Malta, O. L.; Longo, R. L. A Tutorial Review on the Nonradiative Energy Transfer Rates between Lanthanide Ions, *Chinese J. Lumin.* **2022**, 43, 1871.
